# In silico-guided sequence modifications of K-ras epitopes improve immunological outcome against G12V and G13D mutant *KRAS* antigens

**DOI:** 10.7717/peerj.5056

**Published:** 2018-07-20

**Authors:** Allan Wee Ren Ng, Pei Jun Tan, Winfrey Pui Yee Hoo, Dek Shen Liew, Michelle Yee Mun Teo, Pui Yan Siak, Sze Man Ng, Ee Wern Tan, Raha Abdul Rahim, Renee Lay Hong Lim, Adelene Ai Lian Song, Lionel Lian Aun In

**Affiliations:** 1 Department of Biotechnology, Faculty of Applied Sciences, UCSI University, Cheras, Wilayah Persekutuan Kuala Lumpur, Malaysia; 2 Department of Cell and Molecular Biology, Faculty of Biotechnology and Biomolecular Sciences, Universiti Putra Malaysia, UPM Serdang, Selangor, Malaysia; 3 Department of Microbiology, Faculty of Biotechnology and Biomolecular Sciences, Universiti Putra Malaysia, UPM Serdang, Selangor, Malaysia

**Keywords:** Mimotope, *KRAS*, In silico prediction, Immunogen, IEDB, Peptide vaccine

## Abstract

**Background:**

Somatic point substitution mutations in the *KRAS* proto-oncogene primarily affect codons 12/13 where glycine is converted into other amino acids, and are highly prevalent in pancreatic, colorectal, and non-small cell lung cancers. These cohorts are non-responsive to anti-EGFR treatments, and are left with non-specific chemotherapy regimens as their sole treatment options. In the past, the development of peptide vaccines for cancer treatment was reported to have poor AT properties when inducing immune responses. Utilization of bioinformatics tools have since become an interesting approach in improving the design of peptide vaccines based on T- and B-cell epitope predictions.

**Methods:**

In this study, the region spanning exon 2 from the 4th to 18th codon within the peptide sequence of wt*KRAS* was chosen for sequence manipulation. Mutated G12V and G13D K-ras controls were generated in silico, along with additional single amino acid substitutions flanking the original codon 12/13 mutations. IEDB was used for assessing human and mouse MHC class I/II epitope predictions, as well as linear B-cell epitopes predictions, while RNA secondary structure prediction was performed via CENTROIDFOLD. A scoring and ranking system was established in order to shortlist top mimotopes whereby normalized and reducing weighted scores were assigned to peptide sequences based on seven immunological parameters. Among the top 20 ranked peptide sequences, peptides of three mimotopes were synthesized and subjected to in vitro and in vivo immunoassays. Mice PBMCs were treated in vitro and subjected to cytokine assessment using CBA assay. Thereafter, mice were immunized and sera were subjected to IgG-based ELISA.

**Results:**

In silico immunogenicity prediction using IEDB tools shortlisted one G12V mimotope (68-V) and two G13D mimotopes (164-D, 224-D) from a total of 1,680 candidates. Shortlisted mimotopes were predicted to promote high MHC-II and -I affinities with optimized B-cell epitopes. CBA assay indicated that: 224-D induced secretions of IL-4, IL-5, IL-10, IL-12p70, and IL-21; 164-D triggered IL-10 and TNF-α; while 68-V showed no immunological responses. Specific-IgG sera titers against mutated K-ras antigens from 164-D immunized Balb/c mice were also elevated post first and second boosters compared to wild-type and G12/G13 controls.

**Discussion:**

In silico-guided predictions of mutated K-ras T- and B-cell epitopes were successful in identifying two immunogens with high predictive scores, Th-bias cytokine induction and IgG-specific stimulation. Developments of such immunogens are potentially useful for future immunotherapeutic and diagnostic applications against *KRAS*(+) malignancies, monoclonal antibody production, and various other research and development initiatives.

## Introduction

Among the Ras oncogene superfamily, K-Ras is the most frequently mutated isoform harboring somatic point substitution mutations at codons 12/13 in the *KRAS* proto-oncogene in most cancers, with prevalence of 90% in PDAC patients, 40% in CRC patients, and 25% in NSCLC patients (Bhattacharya, Socinski & Burns, 2015; Yoon et al., 2014; Prior, Lewis & Mattos, 2012). *KRAS* is the human homologue to *KRAS*-2 viral oncogene which encodes K-ras, a GTP-binding protein, involved upstream of RAS/RAF/MAPK and PI3K/AKT pathways. The protein cycles from a GDP-bound (“off”) state to a GTP-bound (“on”) state in response to activation by various RTKs (Friday & Adjei, 2005). Generally, these Ras proteins regulate cell growth, differentiation, and survival of cells through a series of cellular signaling cascades (Lièvre et al., 2013). Once mutated, *KRAS* often leads to constant activation of the cell cycle and downstream proliferative pathways. Such mutations have also been linked with resistance to cetuximab and panitumumab for CRC patients, and erlotinib and gefitinib for NSCLC patients, rendering anti-EGFR treatments relatively ineffective among *KRAS*(+) patients (Bournet et al., 2016; Tan & Du, 2012; Pao et al., 2005). Following this, genetic testing as an effort to classify cancer patients between *EGFR*(+) and *KRAS*(+) before prescribing anti-EGFR mAbs have been emphasized worldwide (French et al., 2011).

Past studies have reported that vaccines arising from self-epitopes of tumor-specific or -associated antigens (TSAs or TAAs) were able to stimulate both Th and CTL responses, and may potentially serve as either prophylactic or therapeutic vaccines (Nezafat et al., 2014; Buonaguro et al., 2011). This provides hope for the development of safer alternative treatments for *KRAS*(+) cancer patients owing to the specific-targeting nature of vaccines mediated by the immune system (Tol et al., 2009). However, the development of self-epitope vaccines for clinical use is still in its infancy, and is often perturbed by weak immune responses which are incapable of mounting sufficient clearing of malignancies. This is further aggravated during clinical trials where HLA diversity also seems to affect the selective applicability of such therapies (Carreno et al., 2015).

Mimotopes, which are modified mimics of natural epitopes, have been hypothesized to potentially trigger enhanced cellular or humoral responses, being capable of secreting response-specific cytokines, stimulating production of antibodies, having superior MHC-restriction capabilities and potentially developing immunological memory against targeted TAAs/TSAs, thus potentially preventing cancer occurrence or relapse (Oyarzun et al., 2015). Through bioinformatics tools, design and development of mimotopic sequences have allowed the predictive discrimination of epitopes that may be potentially immunogenic from epitopes that are not. Examples of common immunological parameters assessed includes MHC class I/II binding, HY, SA, AT, epitope linearity, and RNA secondary structure predictions (Soria-Guerra et al., 2015; Patronov & Doytchinova, 2013). While the use of various immunological predictors and tools have been shown to ease the selection of potential mimotopes, their multifactorial immuno-properties and complexity also raises the question of which parameter is arguably superior. Therefore, this study reports on a proof-of-concept highlighting a proposed ranking system comprised of priority-weighted immunological parameters, followed by in vitro and in vivo immunoassay validation data.

## Materials and Methods

### K-ras protein sequence and in silico amino acid substitutions

The peptide sequence of wt*KRAS* (accession number AGC09594) was obtained from Genbank, NCBI. From a full length sequence (189 amino acids), a region spanning exon 2 from the 4th to 18th codon (YKLVVVGAGGVGKSA) was chosen for sequence manipulation. Mutated K-ras controls harboring substitutions of G12V or G13D were generated in silico. Additional single amino acid substitutions flanking the original codon 12/13 mutations were generated from 19 different amino acid possibilities, thus yielding a total of 1,680 query sequences for subsequent T- and B-cell epitope predictions.

### In silico immunogenicity predictions

Immune Epitope Database and Analysis Resource (http://www.iedb.org/) was used for assessing MHC class I/II epitope predictions. Human MHC class I epitope predictions covered HLA-A/B/C alleles, while HLA supertypes (34 allelic subtypes) covered highly polymorphic HLA-DR/DP/DQ MHC class II alleles. On the other hand, mouse MHC class I epitope predictions covered H-2D, H-2K and H-2L alleles, while predictions for MHC class II cover H-2A (I-A) and H-2E (I-E) alleles. Since Balb/c mice were used in this study, only *d* haplotypes: H-2D^d^, H-2K^d^, H-2L^d^, H-2A (I-A^d^), and H-2E (I-E^d^) subclasses were assessed during in silico predictions. Predictions of linear B-cell epitopes and HY stretches were done using the BepiPred method, which is a combination of a HMM and two amino acid propensity scales: the Parker’s HY scale and Levitt’s secondary structure scale (Brown et al., 1993; Parker, Guo & Hodges, 1986; Levitt, 1978). Kolaskar and Tongaonkar AT scale was used to score mimotope sequences for APC recognition via the IEDB server. Prediction of hexapeptide mimotope regions for SA was based on Emini’s SA scale. Normalized scoring ranges for all prediction parameters are shown in Table 1. RNA secondary structure prediction was performed via CENTROIDFOLD (http://www.ncrna.org/centroidfold/) using mimotope RNA transcriptional sequences upon incorporation into a pNZ8048 lactococcal plasmid, which is the vector used for protein expression.

**Table 1:** Predicted in silico raw score conversion scheme for each immunological parameter.

Parameter (max. weightage)	Raw score range for conversion (max. weightage (%))		
	High	Intermediate	Low
MHC class II (58%)	0.01–4.65 nM, (58%)	4.66–9.28 nM, (39%)	≥9.29 nM, (20%)
MHC class I (42%)	0–50 nM, (42%)	51–500 nM, (28%)	501–5,000 nM, (14%)
SA^a^ (34%)	2.531–3.149, (34%)	1.913–2.530, (23%)	1.294–1.912, (12%)
HY^b^ (29%)	3.444–4.614, (29%)	2.615–3.443, (19%)	0–2.614, (9%)
AT^c^ (19%)	≥1.0, (19%)	<1.0, (13%)	*N/A*
LE^d^ (12%)	≥5.82, (12%)	0.36–5.81, (8%)	0–0.35, (4%)
RNA 2°^e^ (6%)	Does not form hairpin loop, (6%)	*N/A*	Forms hairpin loop, (4%)

**Notes:**

*N/A*, Not applicable.

a Surface accessibility.

b Hydrophilicity.

c Antigenicity.

d Linear epitope.

e RNA secondary structure.

### Establishment of a scoring and ranking system

A normalized scoring and ranking system was established after obtaining raw scores in each prediction in order to shortlist top mimotopes. MHC class II epitope prediction was selected as the highest priority parameter, followed by decreasing prioritized predictions of MHC class I epitope, LE prediction, HY prediction, AT prediction, accessibility prediction, and RNA secondary structure prediction respectively. All seven parameters were assigned with various score ranges, whereby the normalized weighted scores assigned to peptide sequences were reduced in accordance with a decrease in priority: peptides having high MHC class II restriction > MHC class I restriction > surface accessibility > HY > AT > epitope linearity > RNA secondary structure. Summing up the scores from all categories yielded a total normalized percentile score, thus creating a series of ranked mimotopes in descending order of immunogenicity.

### Synthesis of peptide mimotopes

Three mimotope sequences as highlighted in Tables 2–4 representing various codon 12/13 mutations were selected based on scores obtained from in silico predictions and were synthesized by Mimotopes International, Australia. An additional three mimotopes harboring G12V or G13D mutations, and wild-type K-ras were also synthesized as controls for subsequent immunoassays.

**Table 2:** Top 20 shortlisted peptide sequence candidates with scores generated from human MHC class I and II epitope prediction parameters.

Sequence ID	Full sequence	*KRAS* mutation	Modified residue	MHC class II^a^	MHC class I^b^	Total score^c^
68-D	YKLDVVGADGVGKSA	G12D	V7D	58	42	100
68-A	YKLDVVGAAGVGKSA	G12A	V7D	58	42	100
68-S	YKLDVVGASGVGKSA	G12S	V7D	58	42	100
68-V*	YKLDVVGAVGVGKSA	G12V	V7D	58	42	100
93-V	YKLVLVGAVGVGKSA	G12V	V8L	58	42	100
139-A	YKLVVVPAAGVGKSA	G12A	G10P	58	42	100
64-D	YKLYVVGADGVGKSA	G12D	V7Y	58	42	100
103-R	YKLVVAGARGVGKSA	G12R	V9A	58	42	100
116-R	YKLVVHGARGVGKSA	G12R	V9H	58	42	100
135-D	YKLVVVIADGVGKSA	G12D	G10I	58	42	100
138-S	YKLVVVKASGVGKSA	G12S	G10K	58	42	100
112-A	YKLVVNGAAGVGKSA	G12A	V9N	58	42	100
164-D*	YKLVVVGAGDVYKSA	G13D	G15Y	58	42	100
133-S	YKLVVVLASGVGKSA	G12S	G10L	58	42	100
135-A	YKLVVVIAAGVGKSA	G12A	G10I	58	42	100
84-S	YKLVYVGASGVGKSA	G12S	V8Y	58	42	100
78-D	YKLDVVGAVDVGKSA	G13D	V7D	58	42	100
224-D*	YKLVVVGAGDVGKSY	G13D	A18Y	58	42	100
67-D	YKLTVVGAGDVGKSA	G13D	V7T	58	42	100
194-D	YKLVVVGAGDVGQSA	G13D	K16Q	58	42	100
Control 1*	YKLVVVGAVGVGKSA	G12V	–	58	42	100
Control 2*	YKLVVVGAGDVGKSA	G13D	–	58	42	100
wtKRAS*	YKLVVVGAGGVGKSA	–	–	58	42	100

**Notes:**

Sequence ID denoted (*) were selected for immunogenicity assessment.

a Total score converted (as referred to Table 1) from the percentile score of human MHC class II epitope prediction generated from IEDB.

b Total score converted (as referred to Table 1) from the IC_50_ score of human MHC class I epitope prediction generated from IEDB.

c Total score of ^*a*^ and ^*b*^.

**Table 3:** Top 20 shortlisted peptide sequence candidates with scores generated from mouse MHC class I and II epitope prediction parameters.

Sequence ID	Full sequence	*KRAS* mutation	Modified residue	MHC class II^a^	MHC class I^b^	Total score^c^
138-S	YKLVVVKASGVGKSA	G12S	G10K	58	28	86
84-S	YKLVYVGASGVGKSA	G12S	V8Y	39	42	81
135-A	YKLVVVIAAGVGKSA	G12A	G10I	39	28	67
139-A	YKLVVVPAAGVGKSA	G12A	G10P	39	28	67
93-V	YKLVLVGAVGVGKSA	G12V	V8L	39	28	67
78-D	YKLDVVGAVDVGKSA	G13D	V7D	39	28	67
164-D*	YKLVVVGAGDVYKSA	G13D	G15Y	39	28	67
68-A	YKLDVVGAAGVGKSA	G12A	V7D	20	42	62
68-V*	YKLDVVGAVGVGKSA	G12V	V7D	20	42	62
64-D	YKLYVVGADGVGKSA	G12D	V7Y	20	42	62
68-S	YKLDVVGASGVGKSA	G12S	V7D	20	42	62
103-R	YKLVVAGARGVGKSA	G12R	V9A	20	42	62
112-A	YKLVVNGAAGVGKSA	G12A	V9N	20	42	62
67-D	YKLTVVGAGDVGKSA	G13D	V7T	20	42	62
68-D	YKLDVVGADGVGKSA	G12D	V7D	20	28	48
133-S	YKLVVVLASGVGKSA	G12S	G10L	20	28	48
224-D*	YKLVVVGAGDVGKSY	G13D	A18Y	20	28	48
135-D	YKLVVVIADGVGKSA	G12D	G10I	20	28	48
116-R	YKLVVHGARGVGKSA	G12R	V9H	20	28	48
194-D	YKLVVVGAGDVGQSA	G13D	K16Q	20	28	48
Control 1*	YKLVVVGAVGVGKSA	G12V	–	20	28	48
Control 2*	YKLVVVGAGDVGKSA	G13D	–	20	28	48
wtKRAS*	YKLVVVGAGGVGKSA	–	–	20	28	48

**Notes:**

Sequence ID denoted (*) were selected for immunogenicity assessment.

a Total score converted (as referred to Table 1) from the percentile score of mouse MHC class II epitope prediction generated from IEDB.

b Total score converted (as referred to Table 1) from the IC_50_ score of mouse MHC class I epitope prediction generated from IEDB.

c Total score of ^*a*^ and ^*b*^.

**Table 4:** Top 20 shortlisted peptide sequence candidates with scores generated from each B-cell epitope prediction parameter in human and mouse genomes.

Sequence ID	Full sequence	*KRAS* mutation	Modified residue	SA^a^	HY^b^	AT^c^	LE^d^	RNA 2°^e^	Total score^f^
135-D	YKLVVVIADGVGKSA	G12D	G10I	34	29	19	8	6	96
224-D*	YKLVVVGAGDVGKSY	G13D	A18Y	34	29	13	12	6	94
164-D*	YKLVVVGAGDVYKSA	G13D	G15Y	34	19	19	8	6	86
133-S	YKLVVVLASGVGKSA	G12S	G10L	23	29	19	8	6	85
68-D	YKLDVVGADGVGKSA	G12D	V7D	23	29	13	12	6	83
67-D	YKLTVVGAGDVGKSA	G13D	V7T	23	29	13	12	6	83
64-D	YKLYVVGADGVGKSA	G12D	V7Y	23	29	13	12	6	83
194-D	YKLVVVGAGDVGQSA	G13D	K16Q	23	29	13	12	6	83
Control 2*	YKLVVVGAGDVGKSA	G13D	–	23	29	13	12	6	83
wtKRAS*	YKLVVVGAGGVGKSA	–	–	23	29	13	12	6	83
68-S	YKLDVVGASGVGKSA	G12S	V7D	12	29	19	12	6	78
84-S	YKLVYVGASGVGKSA	G12S	V8Y	12	29	19	12	6	78
135-A	YKLVVVIAAGVGKSA	G12A	G10I	23	19	19	8	6	75
138-S	YKLVVVKASGVGKSA	G12S	G10K	12	29	19	8	6	74
103-R	YKLVVAGARGVGKSA	G12R	V9A	23	19	13	12	6	73
116-R	YKLVVHGARGVGKSA	G12R	V9H	23	19	13	12	6	73
78-D	YKLDVVGAGDVGKSA	G13D	V7D	12	29	13	12	6	72
68-A	YKLDVVGAAGVGKSA	G12A	V7D	12	19	19	12	6	68
139-A	YKLVVVPAAGVGKSA	G12A	G10P	12	19	19	12	6	68
112-A	YKLVVNGAAGVGKSA	G12A	V9N	12	29	13	12	6	72
68-V*	YKLDVVGAVGVGKSA	G12V	V7D	12	9	19	8	6	54
93-V	YKLVLVGAVGVGKSA	G12V	V8L	12	9	19	8	6	54
Control 1*	YKLVVVGAVGVGKSA	G12V	–	12	9	19	8	6	54

**Notes:**

Sequence ID denoted (*) were selected for immunogenicity assessment.

a Surface accessibility of the hexapeptide mimotope regions was predicted based on Emini’s surface accessibility scale. Total score was converted (as referred to Table 1) from the raw probability score prediction generated from IEDB server.

b, d Hydrophilicity and B-cell linear epitope were predicted using BepiPred method based on the Parker’s hydrophilicity scale and Levitt’s secondary structure scale. Total score was converted (as referred to Table 1) from the raw score prediction generated from IEDB server.

c Antigenicity was predicted based on Kolaskar and Tongaonkar antigenicity scale to score mimotope sequences for APC recognition. Total score was converted (as referred to Table 1) from the raw score prediction generated from IEDB server.

e RNA secondary structure was predicted using mimotope RNA transcriptional sequences. Total score was assigned (as referred to Table 1) in accordance to the prediction generated from CENTROIDFOLD.

f Total score of ^*a*^, ^*b*^, ^*c*^, ^*d*^ and ^*e*^.

### Cytokine assessment based on cytometric bead array analysis

Peripheral blood mononuclear cells were harvested from 8 to 10 weeks old female Balb/c mice (Universiti Putra Malaysia, Malaysia) through anesthesia with 100 mg/kg of ketamine and 10 mg/kg of xylazil cocktail followed by cardiac puncture and isolation using Ficoll-Paque Plus (GE Healthcare, London, UK). Cell counts were performed using a hemacytometer and 5.0 × 10^4^ cells/well in DMEM were seeded in 96-well plates. PBMCs were incubated at 37 °C overnight and treated with one μg/ml of each mimotope or controls for 24 and 48 h. Treated samples were subjected to BD CBA Mouse Flex Set (BD Biosciences, Franklin Lakes, NJ, USA) according to manufacturer’s protocol for cytokine assessment. Capture beads coated with antibodies against nine different cytokines: IL-2, IL-4, IL-5, IL-6, IL-10, IL-21, IL-12p70, TNF-α, and IFN-γ were incubated in each PBMC culture supernatant sample. PE-conjugated antibodies were added for detection of cytokines, while cytokine quantification was based on a standard curve plot. Fluorescence from PE-conjugated antibodies were detected using BD Accuri™ C6 Plus flow cytometer (BD Biosciences, Franklin Lakes, NJ, USA) and NovoCyte™ flow cytometer (ACEA Biosciences, San Diego, CA, USA) during sample data acquisition, while data analyses were performed using FCAP Array Software v3.0 (BD Biosciences, Franklin Lakes, NJ, USA) and NovoExpress^®^ Software v1.2.4 (ACEA Biosciences, San Diego, CA, USA) respectively. Data from all CBA experiments were presented as mean ± standard deviation of three biological replicates. Differences between samples and controls were considered statistically significant with *p*-values ≤ 0.05(*) or ≤ 0.01(**) as calculated using paired Student’s *t*-test.

### Immunization schedule

Groups of six female 6–8 weeks old Balb/c mice were immunized intraperitoneally with various peptide controls (G13D, G12V), mimotopes (164-D, 224-D, and 68-V) and negative control (saline). Primary immunization on day 1 was performed using 100 μl of peptide mimotopes (one μg/μL) or saline emulsified with complete Freund’s adjuvant (Sigma-Aldrich, St. Louis, MO, USA) in a 1:1 concentration ratio. Boosters of 100 μl of peptide mimotopes (one μg/μL) or saline emulsified with incomplete Freund’s adjuvant (Sigma-Aldrich, St. Louis, MO, USA) in a 1:1 concentration ratio were then administered at two-week intervals (days 14 and 28). Mice sera were collected by retro-orbital bleeding prior to the primary immunization (day 1) and 7 days after each booster (days 21 and 35). Sera were separated from blood by centrifugation at 1,000*g* for 15 minutes at 4 °C for IgG detection. All husbandry and care of animals were carried out in compliance with the ARRIVE guidelines under institutional IACUC ethics approval (Ref. No. 2017-200309/UCSI/R/LILA).

### Detection of mimotope-specific IgG responses by ELISA

Cross-reactivity assessment was validated prior to measuring levels of mimotope-specific IgG by indirect ELISA. MaxiBinding microtiter plates (SPL Life Sciences, Pocheon, Gyeonggi, Korea) coated with 100 μl of each mimotope (five μg/ml) in PBS, pH 7.4 were incubated overnight at 4 °C. After blocking, 100 μl of diluted serum samples from each mouse (1:250) in 1% (w/v) BSA were added, followed by detection using HRP-labeled goat anti-mouse IgG (Vivantis, Santa Cruz Biotechnology, Dallas, Texas, USA) (1:12,000) and TMB substrate solution (Elabscience, Bethesda, MD, USA). The reaction was inhibited by the addition of 50 μl H_2_SO_4_ (1 N) per well, and absorbance values at 450 nm were determined using FLUOstar^®^ Omega (BMG LABTECH GmbH, Ortenberg, Hesse, Germany). IgG titer levels were determined by coating ELISA plates with the same antigens used during each respective immunization. Non-coated wells consist of mimotope-treated sera without antigen coating, while blank controls consist of antigen-coated wells without addition of mimotope-treated sera. Both non-coated wells and blank controls were used as negative controls to identify background signals. All samples and controls were assayed in three to six replicates, and all relative mean data were expressed as net absorbance by subtracting the mean absorbance for wells without the primary antibody assuming each peptide was coated consistently between plates. IgG concentrations were calculated by interpolation from a standard curve and expressed as mean relative IgG concentration ± standard deviation. Differences between pre-immunizations and boosters were analyzed using paired Student’s *t*-test where *p*-values ≤ 0.05(*) or ≤ 0.01(**) were considered statistically significant.

## Results

### Single amino acid substitutions enhance predicted immunogenicity and scoring of mimotopes compared to unmodified K-ras epitopes

Out of 1,680 mimotope candidates harboring point mutations G12V and G13D used in predictions, the top 20 sequences were shortlisted using a normalized scoring and ranking system based on seven immunological parameters. Scores used in this system were converted from raw scores generated by IEDB, and divided into two or three ranges of high, intermediate and low as shown in Table 1. Converted scores for each peptide candidate were summed as the final cumulative score. Codons 4–18 were chosen because MHC class II restriction involves an oligopeptide length of 13–17 residues (De Oliveira Lopes et al., 2013; Ellerin, Rubin & Weinblatt, 2003), while single amino acid substitutions were limited only to MHC groove binding regions flanking codons 12/13. Prediction results indicated that top 20 peptide candidates had substitutions between codons 7–10 or 15–18, suggesting that peptides with MHC binding groove substitutions of at least two residues downstream or upstream from the mutation of interest would perform immunologically better, while modifications further than five residues away were less influential towards the improvement of MHC presentation (Tables 2 and 3). It was also noteworthy that B-cell epitope accessibility scores were improved when flanking regions contained acidic amino acid substitutions such as aspartate, while AT scores were improved when aliphatic amino acids such as leucine or isoleucine were substituted in. Due to the relatively short length of mimotopes, most of the peptides were predicted as hydrophilic with the absence of RNA hairpin loop formation (Table 4) (Nezafat et al., 2014). Despite having a 98.9% similarity in full length K-ras protein sequence and a 100% similarity between codons 4 and 18 between human and mouse (Eddy, 1996), slight variations for T-cell epitope predictions in both species were represented by higher MHC binding affinities in humans compared to mice (Tables 2 and 3). Meanwhile, B-cell epitope predictions showed no variation between both species (Table 4). Three mimotope sequences with point mutations G12V or G13D were then subjected to peptide synthesis for further immunoassay validations.

### Mimotopes 224-D and 164-D trigger Th2 inducer and effector cytokines in mice PBMC cultures

**Figure 1:**
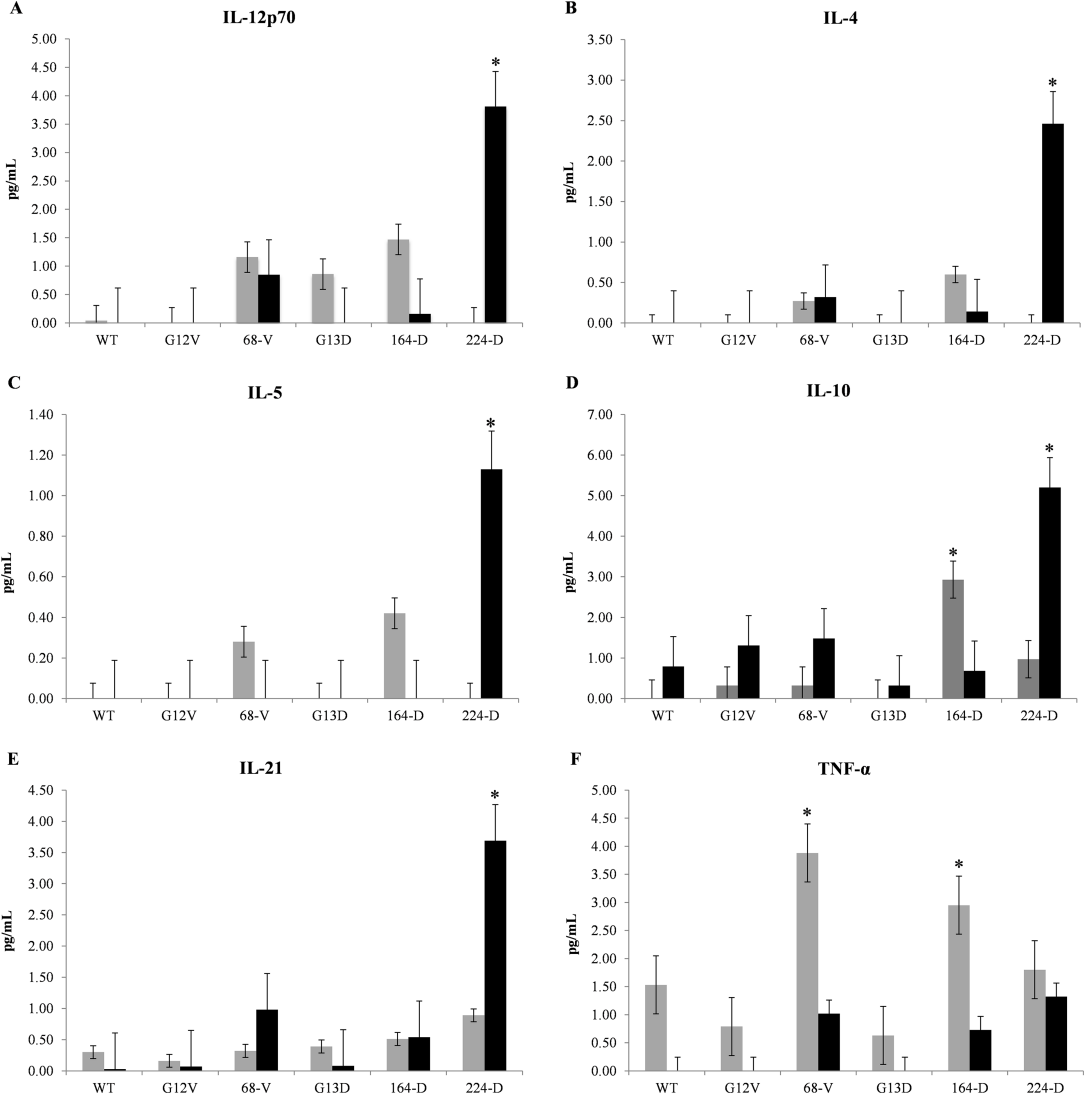
Representative cytometric bead array multiplex immunoassay data of Balb/c mice. Mice PBMCs were treated with one μg/ml of wildtype K-ras (WT), control mimotopes (G12V, G13D) and therapeutic mimotopes (68-V, 164-D, 224-D). Cytokines were assessed by CBA assay and concentrations of: (A) IL-12p70, (B) IL-4, (C) IL-5, (D) IL-10, (E) IL-21, and (F) TNF-α; were shown. Gray bars indicate 24 h exposure while black bars indicate 48 h exposure of PBMCs to respective mimotopes. All values are presented as mean ± standard deviation (*n* = 3) with (*) denoting significant difference (*p* ≤ 0.05) between therapeutic mimotopes with their corresponding controls.

To determine the effectiveness of mimotopes in eliciting an immune response, CBA assays were carried out to evaluate cytokine release profiles from mice PBMCs. Nine representative cytokines were selected to gauge the type of immune response elicited between Th1 and Th2 cell responses, as well as the induction of inflammatory cytokines. Based on Fig. 1, mimotopes 224-D and 164-D significantly promote Th2 immune properties consistent with in silico predictions where elevated levels of IL-4, IL-5, and IL-10 were observed. While exposure to mimotope 224-D did also show elevated levels of IL-12p70 after 48 h of exposure, IL-2 and IFN-γ levels were not significantly affected (Supplementary File S9), suggesting that these mimotopes were unlikely to promote a Th1 immune response. Additionally, production of IL-21 by 224-D was also consistent with a Treg inhibitory cytokine profile (Zhang & An, 2009), thus suggesting an added advantageous property as potential immunotherapeutic candidate. TNF-α, a pro-inflammatory cytokine normally secreted by macrophages (Ellerin, Rubin & Weinblatt, 2003) was found to be stimulated primarily by mimotope 68-V and 164-D. It was also observed that Th-1/2 cytokines generally demonstrated relatively delayed response kinetics compared to TNF-α, with levels spiking at 48 h in the former compared to 24 h in the latter.

### Mimotope 164-D elicits G13D K-ras specific IgG levels in Balb/c mice sera

**Figure 2:**
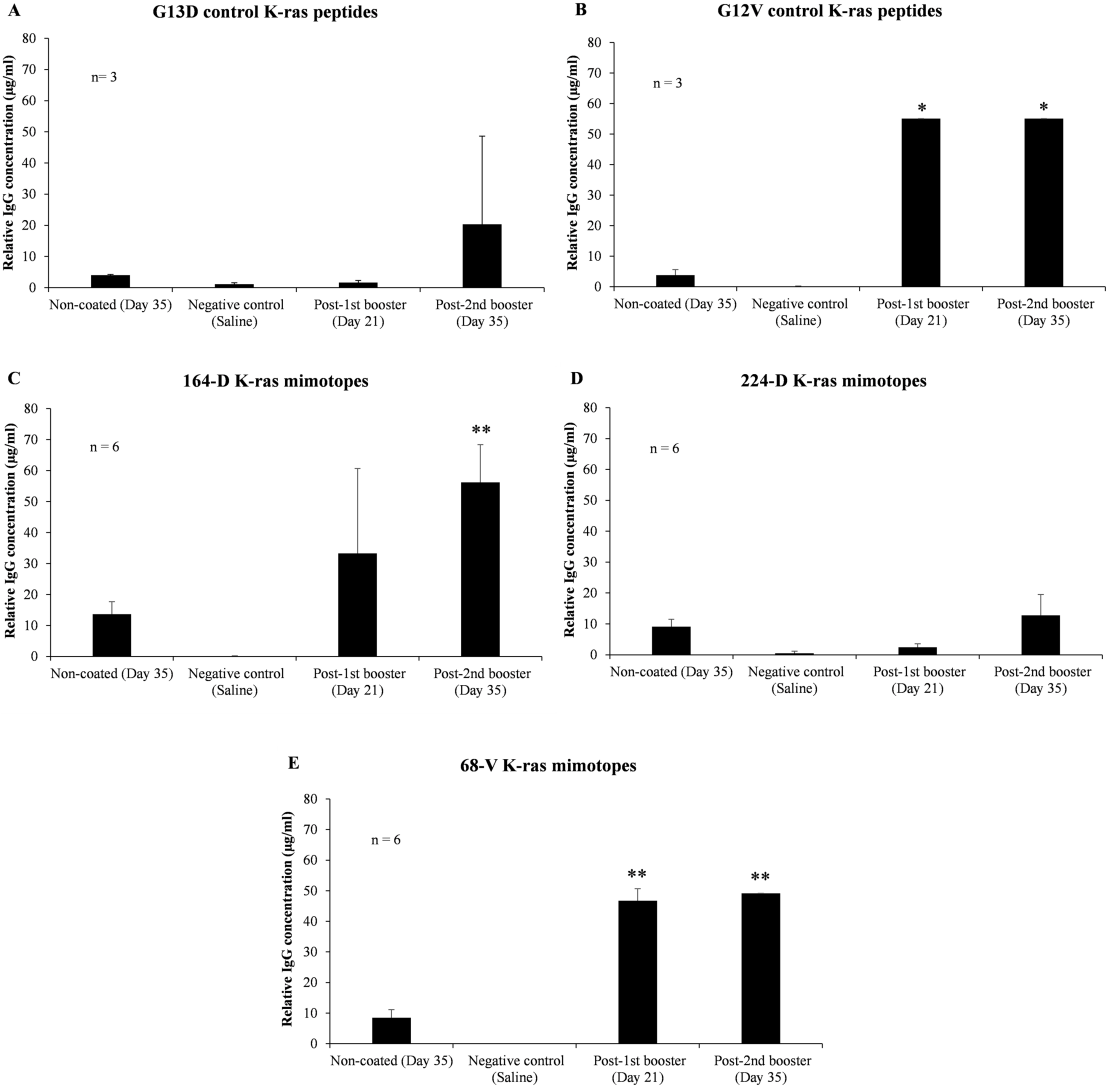
Indirect ELISA on sera level of mutated K-ras specific IgG from mice immunized with control K-ras peptides (G13D, G12V) and K-ras mimotopes (164-D, 224-D, and 68-V). Charts displayed relative IgG concentrations of: (A) G13D control K-ras peptides, (B) G12V control K-ras peptides, (C) 164-D K-ras mimotopes, (D) 224-D K-ras mimotopes, and (E) 68-V K-ras mimotopes. Negative control group is comprised of mice injected with saline, and all mice groups were intraperitoneally administered with mimotopes with complete and incomplete Freund’s adjuvant. Sera samples were collected at day 0 (pre-immunization), 21 (first booster) and 35 (second booster). Sera collected at day 35 were added into non-coated wells as controls to determine the background signals. All data are presented as relative mean values ± standard deviation against background signals from plates without the addition of sera. Significant differences were denoted as *p*-values ≤ 0.05 (*) and *p*-values ≤ 0.01 (**) when compared against negative control.

To verify the effectiveness of 164-D in stimulating a humoral response compared to 224-D and 68-V, mutated K-ras specific IgG levels in mice sera between pre-immunization, first booster and second booster time intervals were assessed. ELISA results indicated that both 164-D and 68-V were found to induce significant elevations in mutated K-ras antigen-specific IgG (*p*-value ≤ 0.01) (Fig. 2). Mimotope 224-D on the other hand, failed to elicit a significant IgG response (*p*-value ≥ 0.05) but could possibly induce cell-mediated activation as a preferred response. After the second booster of G13D control peptide, insignificant levels of IgG (*p*-value ≥ 0.05) were detected compared to pre-immunization levels. On the contrary, G12V control peptide significantly boosted the concentration of IgG (*p*-value ≤ 0.05) to levels comparable of 68-V mimotope, presumably due to the presence of polyclonal population. Therefore, elevation of IgG levels by 68-V was insignificant compared to its G12V control and was unable to induce an antigen-specific humoral immune response. There was also little to no detectable IgG response for the negative control group (saline). Therefore, of the three mimotopes evaluated, 164-D was the only mimotope capable of eliciting an improved immune response compared to its naturally mutated G13D counterpart.

## Discussion

At present, the IMGT/HLA database lists 1,679 HLA class II alleles associated with three classical loci (1,267 DR, 223 DQ and 189 DP alleles), rendering it the most polymorphic gene within the human genome. This vast assortment poses a big obstacle during prediction of potent cancer vaccines because individuals displaying different sets of alleles are likely to respond differently for a given antigen. Therefore, performing T-cell epitope predictions against both human and mouse libraries are important as they provide predictive values of each modified peptide sequence when assessing MHC binding affinities in vivo. Ideally, mimotopes with predicted high MHC-I affinities could subsequently be used to predict MHC-II affinities; however, no apparent good binders to both MHC classes were found. Nevertheless, this study did provide compelling data indicating that modifications on epitope flanking residues of mutated K-ras antigens can influence MHC-epitope binding, thus potentially triggering superior innate or adaptive responses through the relative permissiveness of MHC molecule binding grooves (Soria-Guerra et al., 2015). While IEDB provides a rather comprehensive array of prediction tools and algorithms, it was important to note that an even higher level of T-cell prediction accuracies may be achieved through the use of multiple combinatory prediction software (Levitt, 1978). Unfortunately, subsequent systematic assessment or meta-servers to merge and shortlist dataset scores prior to in vivo evaluation still appears to be lacking at present. Nevertheless, accuracy of T-cell epitope prediction data can be improved through the use of more prediction programs that employ different algorithms. Several other predictions involving TAP binding and proteasomal cleavage sites can also be carried out to enhance the analysis and quality of prediction outcomes.

In B-cell prediction, HMM describes a probability distribution over an infinite number of possible sequences (Huang, Honda & Kanehisa, 2007) and previous studies confirmed that Parker’s HY and Levitt’s index are the best two indices for sequence profiling-based B-cell epitope predictions (Kavitha, Saritha & Vinod, 2013). SA, on the other hand, measures the probability of a hydrophilic peptide being exposed on the surface of MHCs, thus increasing the likelihood for T-cell receptor (TCR) recognition (Srinivasan et al., 2011). Another aspect of B-cell prediction was the use of Kalkaska and Tongaonkar AT scale to predict antigenic determinants within a query sequence (Chang et al., 2006). Both G12/G13 residues of K-ras were also required to appear as part of the LE region, and are crucial for target specific IgG-dependent responses (Baradaran et al., 2013). Besides HMM, Parker’s, and Levitt’s predictors used in this study, combined use of other B-cell predictors such as PREDITOP, BEPITOPE, and BcePred with increased accuracy has also been reported (Kavitha, Saritha & Vinod, 2013).

The efficacy of mimotope vaccines depends on numerous factors such as MHC class I and II restrictions, and B-cell antibody prediction based on a few immunological properties such as SA, epitope linearity, AT, HY, and RNA secondary structure. Each prediction has a specific range of scores assigned to mimotope sequences which will then determine AT of each peptide. The scores given from the IEDB server serve as a rough guideline to determine whether a peptide can be a good binder for both T- and B-cell receptors, which are based on the binding scale adapted from past cohort studies. In MHC class I prediction, peptide sequences scoring less than 500 nM are predicted to have high binding affinities while peptides with scores of 5,000 nM and above would have low binding affinities. As for MHC class II restriction, any peptide scoring from 0.01 to 4.64 nM is predicted to bind into the MHC pocket with higher binding affinity. B-cell epitope predictions such as SA uses a scale that suggests peptide scores of more than one will increase the probability of the epitope being bound on the surface of APCs. Similarly, epitope linearity, which predicts and scores peptide mimotopes containing stretches of residues that could form a part of an antibody epitope utilizes a scale where score of more than 0.35 nM is considered optimum. In this newly established scoring and ranking system, a maximum score of 100% was each assigned to both T- and B-cell epitope predictions. Weightage were divided among the parameters that fall in each epitope prediction, at the same time, making sure that scores of each immunological parameter do not weigh similarly. The order of immunological parameter priority was suggested since highly specific mimotopes would have higher performance in MHC class II restriction rather than MHC class I restriction due to the potential for long term memory immune responses between MHC class II molecules with Th cells when used against a specific cancer subtype. Peptides 68-V, 164-D and 224-D displayed the highest scores among the mutant *KRAS* G12/13 codons when both T- and B-cell prediction scores were evaluated to give an improved immune response, thus explaining the choice of peptide selection for in vitro immunogenicity study.

Vaccines may promote immune responses involving cytotoxic CD8^+^ T-cell, helper CD4^+^ T-cell, or antibody (B-cell) (Li et al., 2014). The induction of CD4^+^ T-cell responses is critical to stimulate CD8^+^ T-cell or antibody responses with regards to the nature of the protective immune response required (Li et al., 2014). In terms of eliciting a humoral response, the induction of IL-4 has been shown to potentially direct the differentiation of naïve T-cells to Th2 cells (Zhang & An, 2009; Jiménez et al., 2005). In response to IL-4 production, IL-5, IL-6, and IL-10 produced by Th2 cells can further promote a strong antibody production, eosinophil activation, and inhibition of several macrophage functions. IL-10 also plays an important anti-inflammatory role as it has been shown to be able to down regulate the production of pro-inflammatory cytokines such as TNF-α (Wang & Lin, 2008), which may explain the inhibition of TNF kinetics as seen in this study between 24 and 48 h of antigen exposure. With regards to cancer, TNF-α level could swing either way to stimulate the growth, proliferation, invasion and metastasis of cancers, or inversely be beneficial through the induction of cell death (Barbacid, 1987). Present CBA data indicated that mimotopes 224-D and 164-D were able to stimulate possible humoral immune responses, where such indications would lead to potential development of CD4^+^ T-cell cancer vaccines with possible inclusion of B-cell epitopes. Therefore, the incorporation of MHC class II restricted epitopes to activate CD4^+^ T-cells and/or B-cell epitopes can be exploited to promote adaptive immune response which can be a more effective and robust way to eliminate tumors through activation of Th and antibody-mediated responses (Comber & Philip, 2014; LeBien & Tedder, 2008).

While most cancer vaccines in the past typically employs a cell-mediated response, the detection of mutated K-ras specific IgG titers in immunized mice sera following first and second boosters in one of the three mimotopes tested thus far were an interesting find. The conventional paradigm of using antibodies to target only surface antigens is slowly changing, with several reports recently indicating that immunogens capable of eliciting antibodies or intrabodies targeting inner membrane-bound antigens as well as other intracellular TSAs can have therapeutic use against various cancer types (Amici et al., 2016; Marschall & Dübel, 2016; Weidle et al., 2013; Noguchi et al., 2012). Recent clinical trial reports highlighting the success of GI-4000 and TG01 as K-ras vaccines in NSCLC and PDAC respectively have also altered the conventional presumption dictating the inability of immune responses to target inner-membrane bound cancer antigens such as K-ras (Eriksen et al., 2017; Bournet et al., 2016; Chaft et al., 2014). GI-4000 *KRAS* vaccine completed Phase II study in 2014 involving patients with stage I–III NSCLC (Chaft et al., 2014). The vaccine developed by Globeimmune was engineered to activate CD4^+^ helper T-cells and CD8^+^ killer T-cells through stimulation of targeted molecular immunogens intact with heat-inactivated yeast containing modified human RAS protein (Hartley et al., 2015). Targovax’s TG01 has also completed Phase I/II study in PDAC, whereby mutant RAS specific T-cell responses were enhanced by co-administration of GM-CSF to increase survival, and improve safety and tolerability (Palmer et al., 2017).

Immunoglobulins G were highly elevated post first and second boosters of 164-D when compared against G13D control; meanwhile the IgG elevation by G12V control was higher when compared with 68-V. IgG response by 164-D could indicate that sequence modifications at G15Y could improve B-cell epitope AT. Moreover, we also found that IgGs raised when immunized with 164-D were extremely specific towards G13D K-ras antigens respectively, without showing any signs of binding towards other G12/G13 K-ras mutant variants. This degree of specificity can potentially open the floodgates of utilizing such antibodies in numerous downstream applications including the production of mAbs via humanization and hybridoma technologies, scFv or intrabody technology, clinical diagnostics of *KRAS* mutational status in *EGFR*(+) cancer patients, as well as research and developmental efforts worldwide.

Despite promising findings in this study, several concerns have been raised over the limitations of T- and B-cell epitope predictions. Among these limitations is the use of only 34 HLA class II alleles when predicting CD4^+^ T-cell epitopes. This poses a big limitation because different alleles are expressed at different frequencies among ethnicities, thus reducing its coverage between individuals. Scaling methods employing a combination of individually scored properties in this study during B-cell epitope prediction also pose another concern. Evidence against the reliability of most prediction methods using amino acid propensity profiling to identify linear B-cell epitope locations have been reported by several studies (Potocnakova, Bhide & Pulzova, 2016; Blythe & Flower, 2005); suggesting for other sophisticated methods and algorithms covering more features to characterize a continuous epitope. Immune selection pressures may also prevail after an effective immunotherapy due to the ability of the host immune cells to recognize both self-peptides and foreign-peptides; therefore it is important to further assess K-ras mimotopes in vivo if epitope variant immune cells will be subjected to negative selection.

## Conclusion

Current immunoassay validations have highlighted the design and potential development of mimotope 164-D against G13D *KRAS*(+) variants as a cancer vaccine candidate. These validations were, to a certain extent, in line with in silico predictions of T- and B-cell epitopes when assessing in vitro and in vivo immune responses. Nevertheless, more comprehensive in silico approaches and predictions of other parameters and scoring methods can be included to further improve the accuracy and precision of T- and B-cell epitope predictions. Further pre-clinical developmental efforts such as the cloning and expression of these mimotopes using a lactococcal vector for potential oral administration, T-cell specific activation assays, delayed-type hypersensitivity reactions, dosage optimization, toxoids-fused mimotopes for improved immunogenicity, and various pharmacological studies are currently underway as we continue to develop 164-D mimotope into a potential cancer vaccine against G13D *KRAS*(+) malignancies.

## Supplemental Information

10.7717/peerj.5056/supp-1 Supplemental Information 1 Mutant K-Ras G12A peptide sequences. All 240 peptide sequences listed in the file are mutant K-Ras G12A peptides having regions spanning exon 2 from the fourth to eighteenth codon (YKLVVVGAGGVGKSA). Additional single amino acid substitutions flanking the original codon 12 mutations were generated from nineteen different amino acid possibilities.

10.7717/peerj.5056/supp-2 Supplemental Information 2 Mutant K-Ras G12C peptide sequences. All 240 peptide sequences listed in the file are mutant K-Ras G12C peptides having regions spanning exon 2 from the fourth to eighteenth codon (YKLVVVGAGGVGKSA). Additional single amino acid substitutions flanking the original codon 12 mutations were generated from nineteen different amino acid possibilities.

10.7717/peerj.5056/supp-3 Supplemental Information 3 Mutant K-Ras G12D peptide sequences. All 240 peptide sequences listed in the file are mutant K-Ras G12D peptides having regions spanning exon 2 from the fourth to eighteenth codon (YKLVVVGAGGVGKSA). Additional single amino acid substitutions flanking the original codon 12 mutations were generated from nineteen different amino acid possibilities.

10.7717/peerj.5056/supp-4 Supplemental Information 4 Mutant K-Ras G12R peptide sequences. All 240 peptide sequences listed in the file are mutant K-Ras G12R peptides having regions spanning exon 2 from the fourth to eighteenth codon (YKLVVVGAGGVGKSA). Additional single amino acid substitutions flanking the original codon 12 mutations were generated from nineteen different amino acid possibilities.

10.7717/peerj.5056/supp-5 Supplemental Information 5 Mutant K-Ras G12S peptide sequences. All 240 peptide sequences listed in the file are mutant K-Ras G12S peptides having regions spanning exon 2 from the fourth to eighteenth codon (YKLVVVGAGGVGKSA). Additional single amino acid substitutions flanking the original codon 12 mutations were generated from nineteen different amino acid possibilities.

10.7717/peerj.5056/supp-6 Supplemental Information 6 Mutant K-Ras G12V peptide sequences. All 240 peptide sequences listed in the file are mutant K-Ras G12V peptides having regions spanning exon 2 from the fourth to eighteenth codon (YKLVVVGAGGVGKSA). Additional single amino acid substitutions flanking the original codon 12 mutations were generated from nineteen different amino acid possibilities.

10.7717/peerj.5056/supp-7 Supplemental Information 7 Mutant K-Ras G13D peptide sequences. All 240 peptide sequences listed in the file are mutant K-Ras G13D peptides having regions spanning exon 2 from the fourth to eighteenth codon (YKLVVVGAGGVGKSA). Additional single amino acid substitutions flanking the original codon 13 mutations were generated from nineteen different amino acid possibilities.

10.7717/peerj.5056/supp-8 Supplemental Information 8 T- and B-cell epitope in silico prediction. Raw and normalized scores of the epitope were assessed according to the seven-parameter predictions.

10.7717/peerj.5056/supp-9 Supplemental Information 9 Raw data of cytometric bead array assay on mimotopes. All mimotopes and their respective controls were subjected to in vitro assessment of cytokines using cytometric bead array assay. Raw data were displayed as mean values and graphs.

10.7717/peerj.5056/supp-10 Supplemental Information 10 BD Accuri C6 fcs. Report 1. A file generated by BD Bioscience reporting mouse IL-21 component on PBMC samples treated with synthetic peptide mimotopes 68-V, 164-D and 224-D. Untreated PBMC samples and standard curve of IL-21 were also included. All data duplicates are reported in number of events, median fluorescence intensity (MFI), nominal concentration (pg/mL), fitted concentration (%), and percentage of recovery (%).

10.7717/peerj.5056/supp-11 Supplemental Information 11 BD Accuri C6 fcs. Report 2. A file generated by BD Bioscience reporting mouse IL-2, IL-4, IL-5, IL-6, IL-10, IL12p70, TNF-alpha and IFN-gamma components on PBMC samples treated with synthetic peptide mimotopes 68-V, 164-D and 224-D. Untreated PBMC samples and standard curve of all cytokine components were also included. All data duplicates are reported in number of events, median fluorescence intensity (MFI), nominal concentration (pg/mL), fitted concentration (%), and percentage of recovery (%).

10.7717/peerj.5056/supp-12 Supplemental Information 12 BD Accuri C6 fcs. Report 3. A file generated by BD Bioscience reporting mouse IL-2, IL-4, IL-5, IL-6, IL-10, IL12p70, IL-21, TNF-alpha and IFN-gamma components on PBMC samples treated with synthetic peptide mimotopes 68-V, 164-D and 224-D. PBMC sampled treated with synthetic peptide mimotopes wild-type KRAS, G12V and G13D were included as controls. Untreated PBMC samples and standard curve of all cytokine components were also included. All data replicates are reported in number of events, median fluorescence intensity (MFI), nominal concentration (pg/mL), fitted concentration (%), and percentage of recovery (%).

10.7717/peerj.5056/supp-13 Supplemental Information 13 BD Accuri C6 fcs. Report 4. A file generated by BD Bioscience reporting mouse IL-2, IL-4, IL-5, IL-6, IL-10, IL12p70, IL-21, TNF-alpha and IFN-gamma components on PBMC samples treated with synthetic peptide mimotopes 68-V, 164-D and 224-D. PBMC sampled treated with synthetic peptide mimotopes wild-type KRAS, G12V and G13D were included as controls. Untreated PBMC samples and standard curve of all cytokine components were also included. The final single replicate data are reported in number of events, median fluorescence intensity (MFI), nominal concentration (pg/mL), fitted concentration (%), and percentage of recovery (%).

## Abbreviations

AKT: protein kinase B
APC: antigen presenting cell
AT: antigenicity
BSA: bovine serum albumin
CBA: cytometric bead array
CD: cluster of differentiation
CRC: colorectal cancer
CTL: cytotoxic T-cell
DMEM: Dulbecco’s minimal essential medium
EGFR: epidermal growth factor receptor
*EGFR*(+): *EGFR*-positive
ELISA: enzyme-linked immunosorbent assay
GDP: guanosine diphosphate
GM-CSF: granulocyte-macrophage colony-stimulating factor
GTP: guanosine triphosphate
H_2_SO_4_: sulphuric acid
HLA: human leukocyte antigen
HMM: Hidden Markov model
HRP: horseradish peroxidase
HY: hydrophilicity
IC50: half maximal inhibitory concentration
IEDB: Immune Epitope Database and Analysis Resource
IFN-γ: interferon-gamma
IgG: immunoglobulin G
IL: interleukin
IMGT: the international ImMunoGeneTics information system
K-ras: GTP-binding protein
*KRAS*: Kirsten rat sarcoma viral oncogene homolog
*KRAS*(+): *KRAS*-positive
LE: linear epitope
mAbs: monoclonal antibodies
MAPK: mitogen-activated protein kinase
MHC: major histocompatibility complex
N/A: not applicable
NCBI: National Center for Biotechnology Information
NSCLC: non-small cell lung carcinoma
PBMCs: peripheral blood mononuclear cells
PBS: phosphate-buffered saline
PDAC: pancreatic ductal adenocarcinoma
PI3K: phosphatidylinositol-4,5-bisphosphate 3-kinase
RAF: RAF proto-oncogene serine/threonine-protein kinase
RAS: rat sarcoma
RTKs: receptor tyrosine kinases
SA: surface accessibility
scFv: single-chain variable fragment
TAAs: tumor-associated antigens
TAP: transporter associated with antigen processing
Th: T helper
TMB: 3,3′,5,5′-Tetramethylbenzidine
TNF-α: tumor necrosis factor-alpha
Treg: regulatory T-cell
TSAs: tumor-specific antigens
wt*KRAS*: wild-type *KRAS*

## Additional Information and Declarations

### Data Availability

The following information was supplied regarding data availability: All FCS files for the cytometric bead array (CBA) assay can be found on the Open Science Framework: DOI 10.17605/OSF.IO/4KHB5.
