# Supplementary material for: In silico-guided sequence modifications of K-ras epitopes improve immunological outcome against G12V and G13D mutant KRAS antigens
Source: PeerJ. 2018 Jul 20;6:e5056. doi: 10.7717/peerj.5056 (PMC6055689; doi:10.7717/peerj.5056)
Supplement: Supplemental Information 10 — A file generated by BD Bioscience reporting mouse IL-21 component on PBMC samples treated with synthetic peptide mimotopes 68-V, 164-D and 224-D. Untreated PBMC samples and standard curve of IL-21 were also included. All data duplicates are reported in number of events, median fluorescence intensity (MFI), nominal concentration (pg/mL), fitted concentration (%), and percentage of recovery (%). [file peerj-06-5056-s010.pdf]

**Plex Name:**	New Plex
**Created by:**	Administrator
**Creation Date:**	11/7/2016
**Intrument:**	Accuri C6

Instrument

Instrument Name: Accuri C6

Scatter Parameter: SSC-A

Number of Scatter Peaks: 1

Clustering Parameter(s): FL4-A , FL3-A

Reporter Parameter(s): FL2-A

Debris Filter is active!

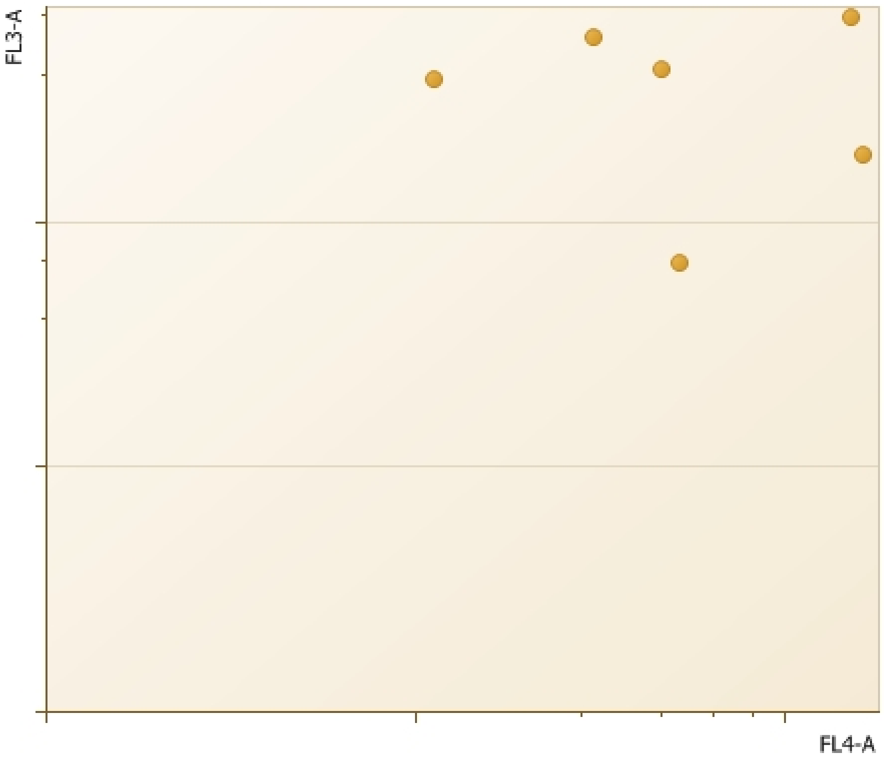

Layout 1

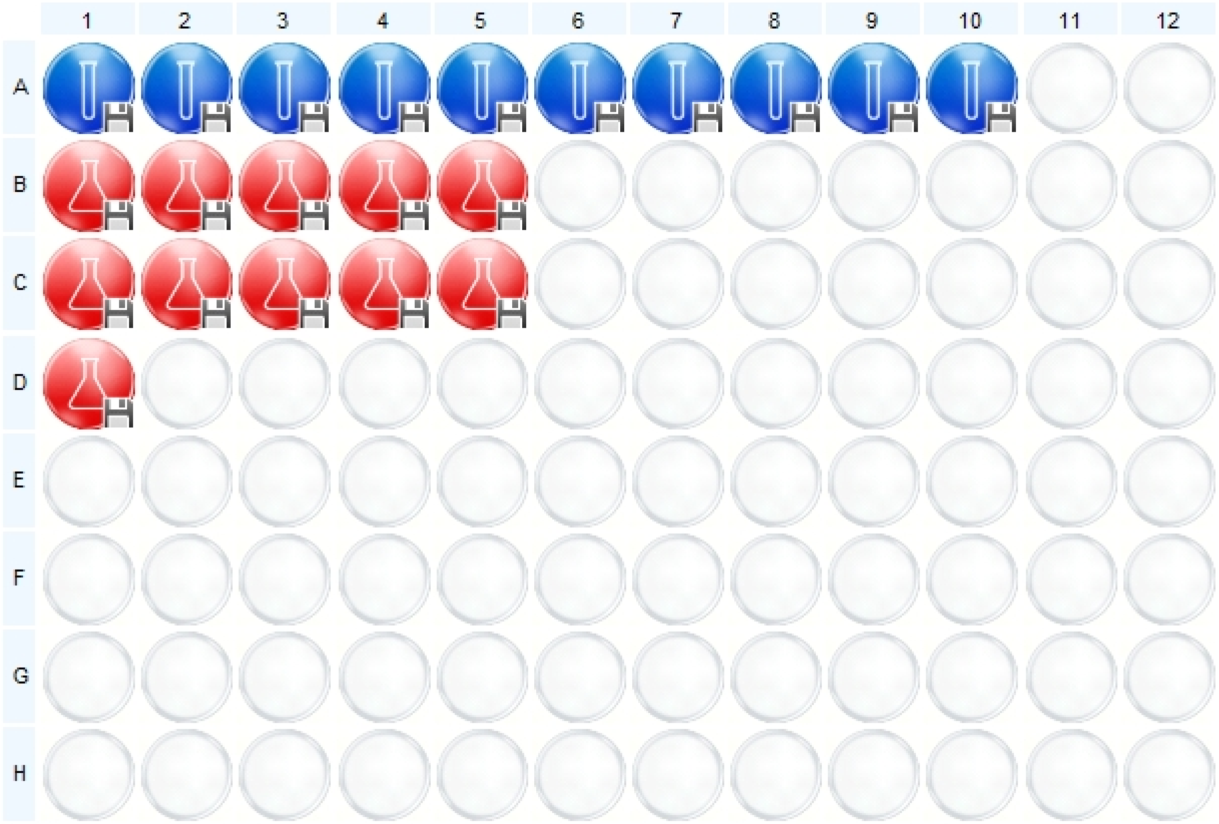

Plex Components

		Analyte		
Name	Lot Number	Name	Model	2nd Reporter
B6		Mouse IL-21	Quantitative	No

Standard Samples of Quantitative Analysis

Reporter Parameter 1	
Sample Name	Concentration
Std001	0.00 pg/mL
Std002	39.06 pg/mL
Std003	78.13 pg/mL

Standard Samples of Quantitative Analysis

Reporter Parameter 1	
Sample Name	Concentration
Std004	156.25 pg/mL
Std005	312.50 pg/mL
Std006	625.00 pg/mL
Std007	1,250.00 pg/mL
Std008	2,500.00 pg/mL
Std009	5,000.00 pg/mL
Std010	10,000.00 pg/mL

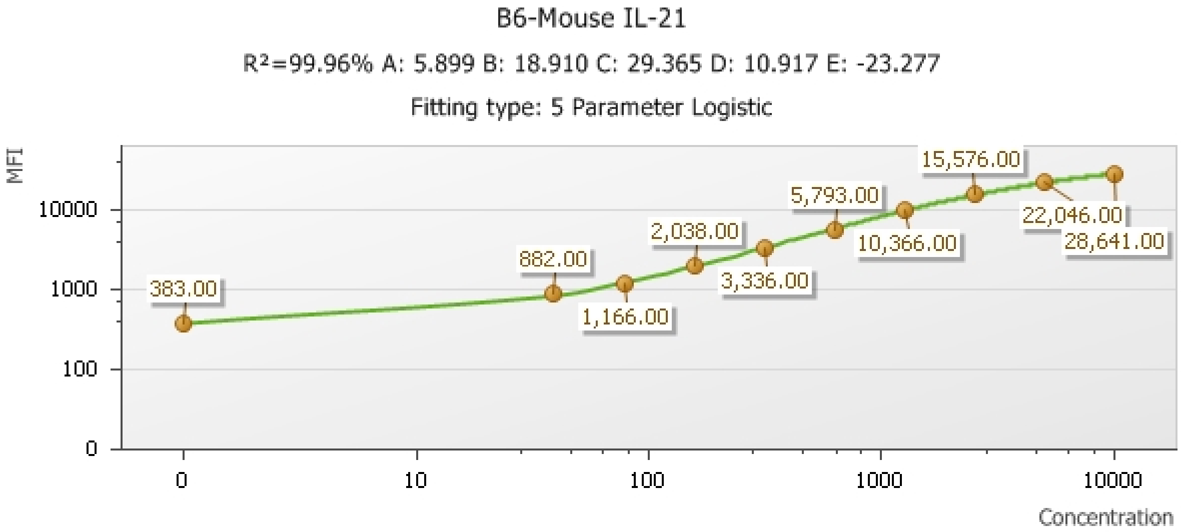

Name	Event #	MFI	SD	CV% (MFI)	Nominal CC	Fitted CC	Recovery %
Std001	768	383.00	330.81	71.87 %	0.00 pg/mL	0.00 pg/mL	0.00 %
Std002	817	882.00	489.26	51.17 %	39.06 pg/mL	43.09 pg/mL	110.30 %
Std003	1089	1,166.00	732.78	61.80 %	78.13 pg/mL	71.51 pg/mL	91.53 %
Std004	810	2,038.00	762.06	48.58 %	156.25 pg/mL	162.22 pg/mL	103.82 %

Name	Event #	MFI	SD	CV% (MFI)	Nominal CC	Fitted CC	Recovery %
Std005	576	3,336.00	978.33	33.32 %	312.50 pg/mL	304.72 pg/mL	97.51 %
Std006	605	5,793.00	1,545.98	24.94 %	625.00 pg/mL	605.70 pg/mL	96.91 %
Std007	507	10,366.00	2,909.60	30.20 %	1,250.00 pg/mL	1,321.03 pg/mL	105.68 %
Std008	541	15,576.00	4,240.24	28.28 %	2,500.00 pg/mL	2,510.23 pg/mL	100.41 %
Std009	517	22,046.00	6,398.53	26.00 %	5,000.00 pg/mL	4,964.39 pg/mL	99.29 %
Std010	657	28,641.00	8,649.86	24.73 %	10,000.00 pg/mL	9,765.38 pg/mL	97.65 %

Results Statistics for B6 - Mouse IL-21

Name	Plate Pos.	Clust.	Event #	MFI	SD	CV	Dilution
Std001	1 - A1	Manual	768	383.00	330.81	71.87 %	1.00
Std002	1 - A2	Manual	817	882.00	489.26	51.17 %	1.00
Std003	1 - A3	Manual	1089	1,166.00	732.78	61.80 %	1.00
Std004	1 - A4	Manual	810	2,038.00	762.06	48.58 %	1.00
Std005	1 - A5	Manual	576	3,336.00	978.33	33.32 %	1.00
Std006	1 - A6	Manual	605	5,793.00	1,545.98	24.94 %	1.00
Std007	1 - A7	Manual	507	10,366.00	2,909.60	30.20 %	1.00
Std008	1 - A8	Manual	541	15,576.00	4,240.24	28.28 %	1.00
Std009	1 - A9	Manual	517	22,046.00	6,398.53	26.00 %	1.00
Std010	1 - A10	Manual	657	28,641.00	8,649.86	24.73 %	1.00
V1B	1 - B1	Manual	660	372.00	303.01	67.46 %	1.00
A1B	1 - B2	Manual	583	329.00	367.68	78.96 %	1.00
139 1 B	1 - B3	Manual	639	386.00	390.67	77.64 %	1.00
224 1 B	1 - B4	Manual	644	423.00	332.10	67.80 %	1.00
164 1 B	1 - B5	Manual	615	397.00	306.16	69.45 %	1.00
V1A	1 - C1	Manual	998	357.00	310.05	72.36 %	1.00
A1A	1 - C2	Manual	976	362.00	296.33	70.96 %	1.00
139 1 A	1 - C3	Manual	837	346.00	314.68	74.25 %	1.00
224 1 A	1 - C4	Manual	765	427.00	313.57	64.56 %	1.00
164 1 A	1 - C5	Manual	743	394.00	271.32	60.17 %	1.00
untreated	1 - D1	Manual	506	364.00	337.66	76.52 %	1.00

Quantitative Analyte Results for B6 - Mouse IL-21

Name	Plate Position	Nominal CC	Fitted CC	Final CC	Message
Std001	1 - A1	0.00 pg/mL	0.00 pg/mL	0.00 pg/mL	Fitting: Below standard range, Recovery: Out of range
Std002	1 - A2	39.06 pg/mL	43.09 pg/mL	43.09 pg/mL	
Std003	1 - A3	78.13 pg/mL	71.51 pg/mL	71.51 pg/mL	
Std004	1 - A4	156.25 pg/mL	162.22 pg/mL	162.22 pg/mL	
Std005	1 - A5	312.50 pg/mL	304.72 pg/mL	304.72 pg/mL	
Std006	1 - A6	625.00 pg/mL	605.70 pg/mL	605.70 pg/mL	
Std007	1 - A7	1,250.00 pg/mL	1,321.03 pg/mL	1,321.03 pg/mL	
Std008	1 - A8	2,500.00 pg/mL	2,510.23 pg/mL	2,510.23 pg/mL	
Std009	1 - A9	5,000.00 pg/mL	4,964.39 pg/mL	4,964.39 pg/mL	
Std010	1 - A10	10,000.00 pg/mL	9,765.38 pg/mL	9,765.38 pg/mL	
V1B	1 - B1	N/A	0.00 pg/mL	0.00 pg/mL	Fitting: Below standard range
A1B	1 - B2	N/A	0.00 pg/mL	0.00 pg/mL	Fitting: Below standard range and out of invertable range
139 1 B	1 - B3	N/A	0.00 pg/mL	0.00 pg/mL	
224 1 B	1 - B4	N/A	2.12 pg/mL	2.12 pg/mL	
164 1 B	1 - B5	N/A	0.51 pg/mL	0.51 pg/mL	
V1A	1 - C1	N/A	0.00 pg/mL	0.00 pg/mL	Fitting: Below standard range and out of invertable range
A1A	1 - C2	N/A	0.00 pg/mL	0.00 pg/mL	Fitting: Below standard range and out of invertable range
139 1 A	1 - C3	N/A	0.00 pg/mL	0.00 pg/mL	Fitting: Below standard range and out of invertable range
224 1 A	1 - C4	N/A	2.39 pg/mL	2.39 pg/mL	

Quantitative Analyte Results for B6 - Mouse IL-21

Name	Plate Position	Nominal CC	Fitted CC	Final CC	Message
164 1 A	1 - C5	N/A	0.34 pg/mL	0.34 pg/mL	
untreated	1 - D1	N/A	0.00 pg/mL	0.00 pg/mL	Fitting: Below standard range and out of invertable range

Sample File Assignment

Sample Name	File Name
Std001	A10 Assay diluent.fcs
Std002	A01 ST 1_256.fcs
Std003	A02 ST 1_128.fcs
Std004	A03 ST 1_64.fcs
Std005	A04 ST 1_32.fcs
Std006	A05 ST 1_16.fcs
Std007	A06 ST 1_8.fcs
Std008	A07 ST 1_4.fcs
Std009	A08 ST 1_2.fcs
Std010	A09 top std.fcs
V1B	B04 V1B 20161014.fcs
A1B	C04 A1B 20161014.fcs
139 1 B	D04 139_1B 20161014.fcs
224 1 B	E04 224_1B 20161014.fcs
164 1 B	F04 164_1B 20161014.fcs
V1A	B04 V1A 20161013.fcs
A1A	C04 A1A 20161013.fcs
139 1 A	D04 139_1 20161013.fcs
224 1 A	E04 224_1 20161013.fcs
164 1 A	F04 164_1 20161013.fcs
untreated	A12 untreated ctrl.fcs
