# Supplementary material for: In silico-guided sequence modifications of K-ras epitopes improve immunological outcome against G12V and G13D mutant KRAS antigens
Source: PeerJ. 2018 Jul 20;6:e5056. doi: 10.7717/peerj.5056 (PMC6055689; doi:10.7717/peerj.5056)
Supplement: Supplemental Information 11 — A file generated by BD Bioscience reporting mouse IL-2, IL-4, IL-5, IL-6, IL-10, IL12p70, TNF-alpha and IFN-gamma components on PBMC samples treated with synthetic peptide mimotopes 68-V, 164-D and 224-D. Untreated PBMC samples and standard curve of all cytokine components were also included. All data duplicates are reported in number of events, median fluorescence intensity (MFI), nominal concentration (pg/mL), fitted concentration (%), and percentage of recovery (%). [file peerj-06-5056-s011.pdf]

**Plex Name:**	UCSI Pei Jun Mouse CBA 9 Plex
**Created by:**	Administrator
**Creation Date:**	11/7/2016
**Intrument:**	Accuri C6

Instrument

Instrument Name: Accuri C6

Scatter Parameter: SSC-A

Number of Scatter Peaks: 1

Clustering Parameter(s): FL4-A , FL3-A

Reporter Parameter(s): FL2-A

Debris Filter is active!

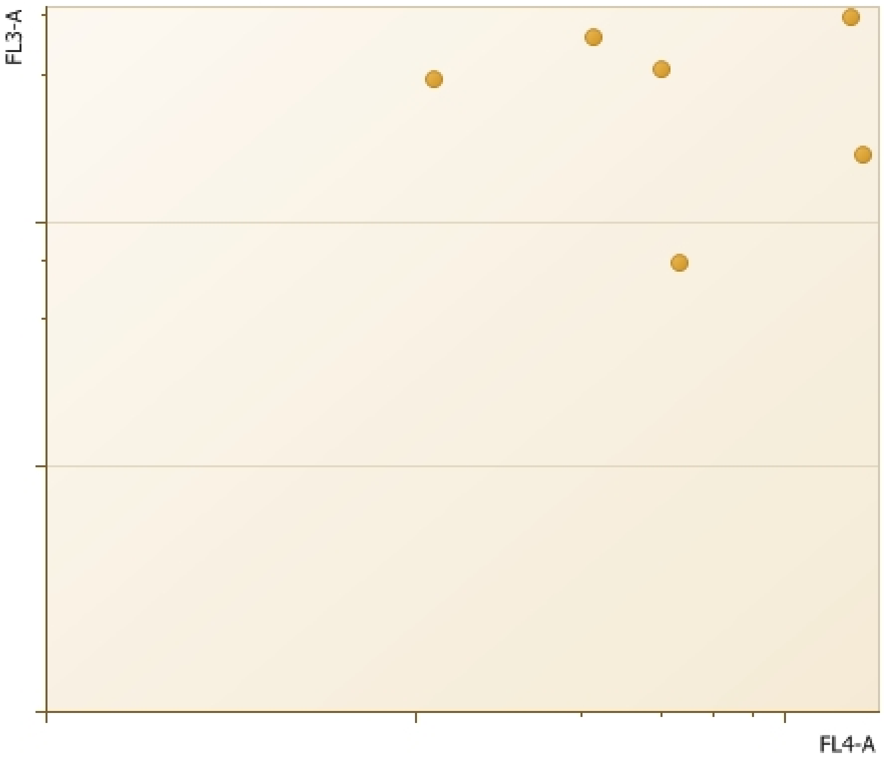

Layout 1

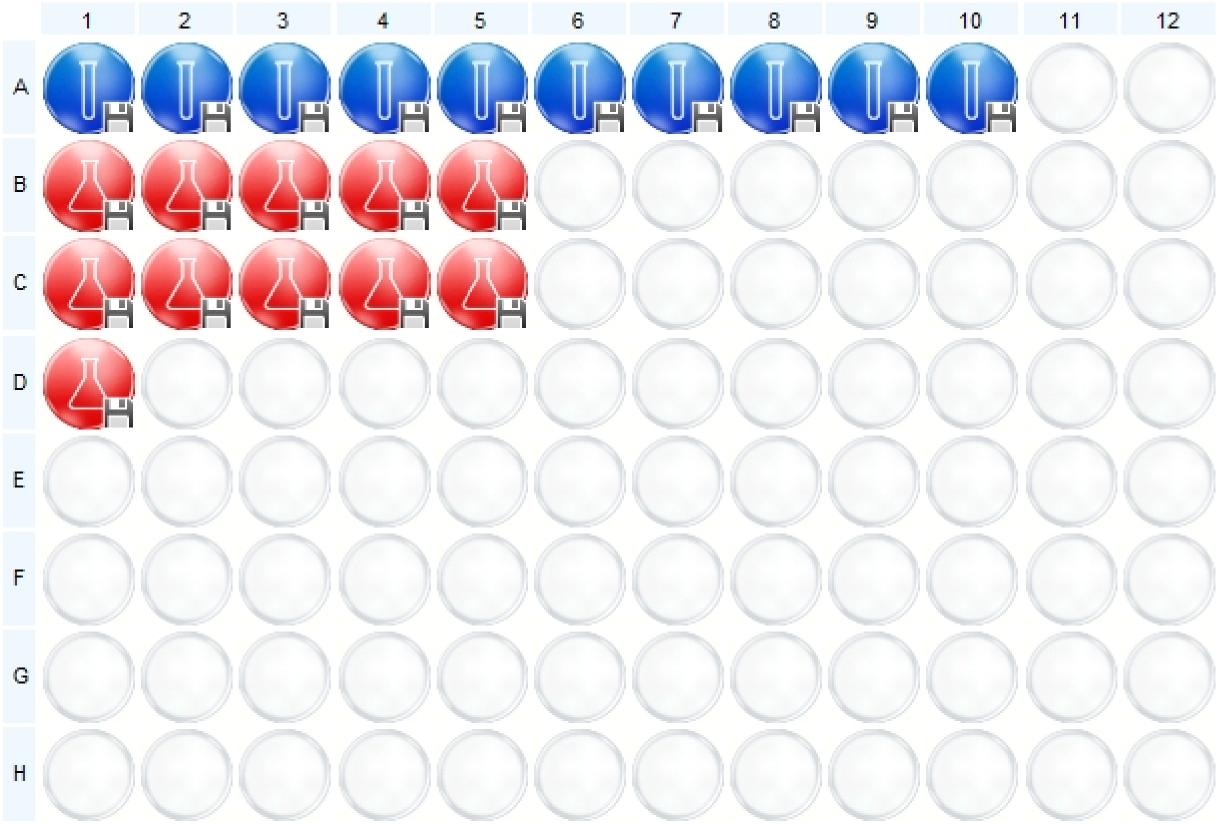

Plex Components

		Analyte		
Name	Lot Number	Name	Model	2nd Reporter
A4		Mouse IFN-γ	Quantitative	No
C4		Mouse IL-10	Quantitative	No
D7		Mouse IL-12p70	Quantitative	No
A5		Mouse IL-2	Quantitative	No
A7		Mouse IL-4	Quantitative	No
A6		Mouse IL-5	Quantitative	No
B4		Mouse IL-6	Quantitative	No
C8		Mouse TNF	Quantitative	No
A8		Mouse IL-3	Quantitative	No

Standard Samples of Quantitative Analysis

Reporter Parameter 1	
Sample Name	Concentration
Std001	0.00 pg/mL
Std002	9.77 pg/mL
Std003	19.53 pg/mL
Std004	39.06 pg/mL
Std005	78.13 pg/mL
Std006	156.25 pg/mL
Std007	312.50 pg/mL
Std008	625.00 pg/mL
Std009	1,250.00 pg/mL
Std010	2,500.00 pg/mL

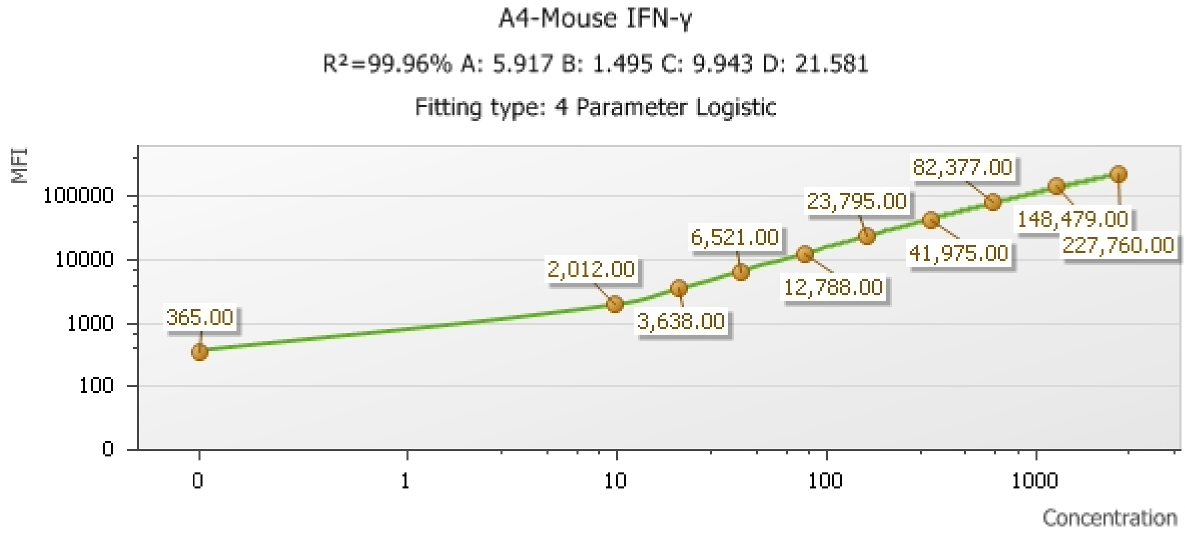

Name	Event #	MFI	SD	CV% (MFI)	Nominal CC	Fitted CC	Recovery %
Std001	1670	365.00	293.55	68.58 %	0.00 pg/mL	0.00 pg/mL	0.00 %
Std002	1541	2,012.00	828.03	39.22 %	9.77 pg/mL	10.26 pg/mL	105.05 %

Name	Event #	MFI	SD	CV% (MFI)	Nominal CC	Fitted CC	Recovery %
Std003	1865	3,638.00	1,363.25	39.58 %	19.53 pg/mL	20.05 pg/mL	102.65 %
Std004	1249	6,521.00	2,396.25	35.94 %	39.06 pg/mL	37.68 pg/mL	96.46 %
Std005	1482	12,788.00	3,674.62	32.79 %	78.13 pg/mL	77.60 pg/mL	99.33 %
Std006	1640	23,795.00	9,441.38	38.43 %	156.25 pg/mL	152.95 pg/mL	97.89 %
Std007	1534	41,975.00	16,643.11	38.86 %	312.50 pg/mL	290.53 pg/mL	92.97 %
Std008	1630	82,377.00	34,350.17	37.71 %	625.00 pg/mL	647.11 pg/mL	103.54 %
Std009	1499	148,479.00	59,269.90	37.41 %	1,250.00 pg/mL	1,362.62 pg/mL	109.01 %
Std010	1514	227,760.00	81,553.38	34.97 %	2,500.00 pg/mL	2,417.33 pg/mL	96.69 %

C4-Mouse IL-10

R^2=99.97% A: 5.706 B: 2.729 C: 7.892 D: 14.767

Fitting type: 4 Parameter Logistic

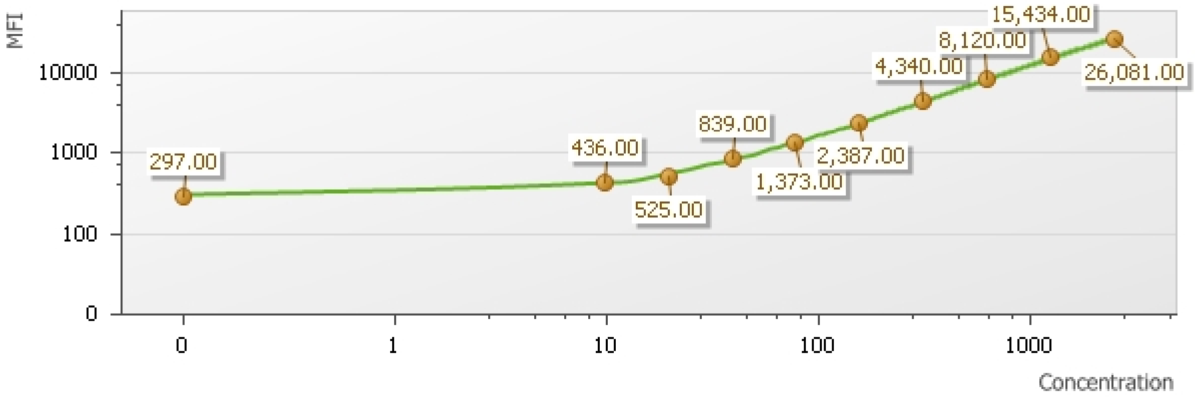

Name	Event #	MFI	SD	CV% (MFI)	Nominal CC	Fitted CC	Recovery %
Std001	788	297.00	311.72	80.14 %	0.00 pg/mL	0.00 pg/mL	0.00 %
Std002	962	436.00	300.23	62.77 %	9.77 pg/mL	11.02 pg/mL	112.87 %
Std003	983	525.00	401.04	65.25 %	19.53 pg/mL	17.32 pg/mL	88.67 %

Name	Event #	MFI	SD	CV% (MFI)	Nominal CC	Fitted CC	Recovery %
Std004	976	839.00	318.02	38.22 %	39.06 pg/mL	39.98 pg/mL	102.34 %
Std005	1063	1,373.00	471.47	31.63 %	78.13 pg/mL	79.40 pg/mL	101.63 %
Std006	792	2,387.00	646.04	27.01 %	156.25 pg/mL	155.76 pg/mL	99.69 %
Std007	876	4,340.00	1,295.79	25.45 %	312.50 pg/mL	307.43 pg/mL	98.38 %
Std008	872	8,120.00	2,226.49	25.42 %	625.00 pg/mL	619.30 pg/mL	99.09 %
Std009	897	15,434.00	4,477.45	25.75 %	1,250.00 pg/mL	1,295.10 pg/mL	103.61 %
Std010	916	26,081.00	7,289.76	24.98 %	2,500.00 pg/mL	2,454.55 pg/mL	98.18 %

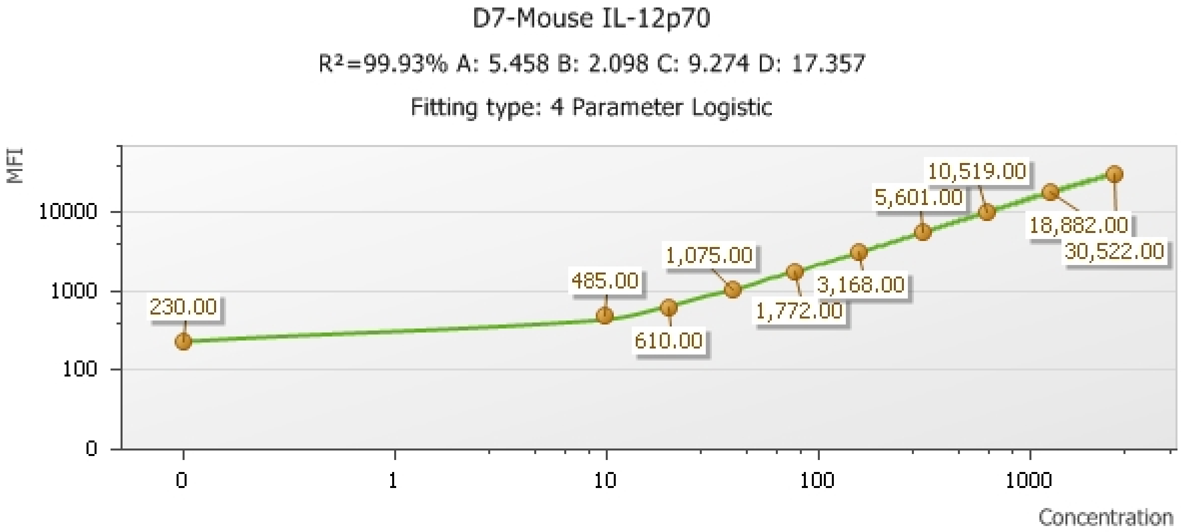

Name	Event #	MFI	SD	CV% (MFI)	Nominal CC	Fitted CC	Recovery %
Std001	685	230.00	323.21	89.97 %	0.00 pg/mL	0.00 pg/mL	0.00 %
Std002	720	485.00	285.96	57.04 %	9.77 pg/mL	11.42 pg/mL	116.90 %
Std003	882	610.00	368.80	57.84 %	19.53 pg/mL	17.17 pg/mL	87.91 %
Std004	600	1,075.00	365.46	32.28 %	39.06 pg/mL	40.01 pg/mL	102.41 %

Name	Event #	MFI	SD	CV% (MFI)	Nominal CC	Fitted CC	Recovery %
Std005	786	1,772.00	537.26	27.36 %	78.13 pg/mL	76.74 pg/mL	98.23 %
Std006	737	3,168.00	691.63	20.22 %	156.25 pg/mL	155.73 pg/mL	99.67 %
Std007	664	5,601.00	1,243.53	19.75 %	312.50 pg/mL	305.40 pg/mL	97.73 %
Std008	686	10,519.00	2,456.67	18.73 %	625.00 pg/mL	643.50 pg/mL	102.96 %
Std009	633	18,882.00	3,123.10	17.72 %	1,250.00 pg/mL	1,308.28 pg/mL	104.66 %
Std010	636	30,522.00	4,574.75	15.90 %	2,500.00 pg/mL	2,398.14 pg/mL	95.93 %

A5-Mouse IL-2

R^2=99.88% A: 5.880 B: 1.704 C: 6.175 D: 16.721

Fitting type: 4 Parameter Logistic

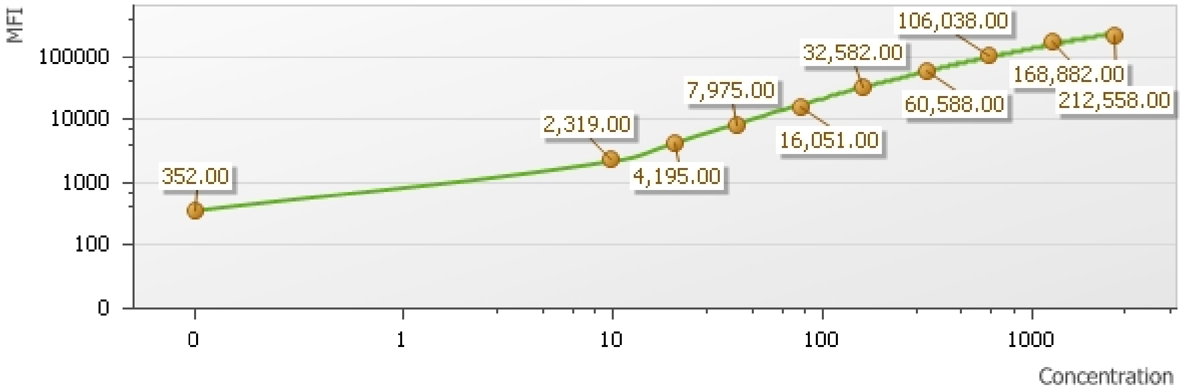

Name	Event #	MFI	SD	CV% (MFI)	Nominal CC	Fitted CC	Recovery %
Std001	794	352.00	295.22	68.12 %	0.00 pg/mL	0.00 pg/mL	0.00 %
Std002	887	2,319.00	646.41	27.16 %	9.77 pg/mL	10.70 pg/mL	109.52 %
Std003	1010	4,195.00	1,117.88	24.25 %	19.53 pg/mL	19.27 pg/mL	98.65 %
Std004	853	7,975.00	2,192.39	24.47 %	39.06 pg/mL	36.08 pg/mL	92.35 %
Std005	989	16,051.00	4,491.54	26.10 %	78.13 pg/mL	72.95 pg/mL	93.37 %

Name	Event #	MFI	SD	CV% (MFI)	Nominal CC	Fitted CC	Recovery %
Std006	830	32,582.00	8,557.01	22.00 %	156.25 pg/mL	156.93 pg/mL	100.44 %
Std007	747	60,588.00	16,679.25	22.49 %	312.50 pg/mL	329.43 pg/mL	105.42 %
Std008	757	106,038.00	27,922.92	20.84 %	625.00 pg/mL	697.11 pg/mL	111.54 %
Std009	687	168,882.00	45,437.98	20.61 %	1,250.00 pg/mL	1,406.33 pg/mL	112.51 %
Std010	692	212,558.00	56,941.48	20.37 %	2,500.00 pg/mL	2,056.59 pg/mL	82.26 %

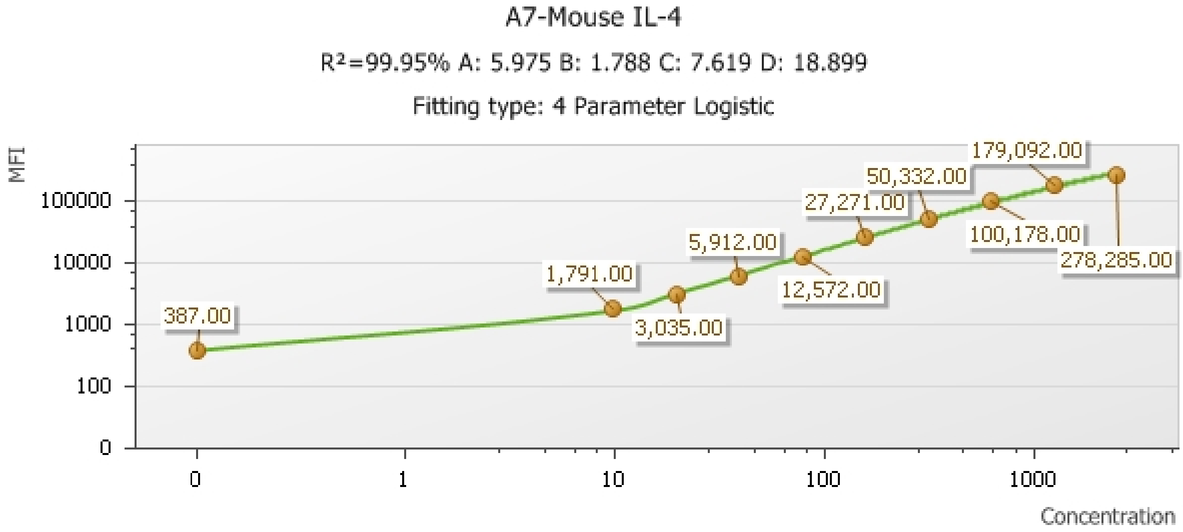

Name	Event #	MFI	SD	CV% (MFI)	Nominal CC	Fitted CC	Recovery %
Std001	1192	387.00	308.38	68.15 %	0.00 pg/mL	0.00 pg/mL	0.00 %
Std002	1122	1,791.00	517.80	26.82 %	9.77 pg/mL	10.75 pg/mL	110.04 %
Std003	1552	3,035.00	922.55	33.82 %	19.53 pg/mL	18.88 pg/mL	96.65 %
Std004	1113	5,912.00	1,879.20	33.83 %	39.06 pg/mL	36.61 pg/mL	93.72 %
Std005	1317	12,572.00	3,953.72	32.32 %	78.13 pg/mL	76.02 pg/mL	97.31 %
Std006	937	27,271.00	7,743.62	24.66 %	156.25 pg/mL	163.18 pg/mL	104.44 %

Name	Event #	MFI	SD	CV% (MFI)	Nominal CC	Fitted CC	Recovery %
Std007	899	50,332.00	15,998.00	27.42 %	312.50 pg/mL	307.29 pg/mL	98.33 %
Std008	1100	100,178.00	30,306.20	30.12 %	625.00 pg/mL	656.26 pg/mL	105.00 %
Std009	921	179,092.00	53,263.89	26.87 %	1,250.00 pg/mL	1,314.65 pg/mL	105.17 %
Std010	905	278,285.00	73,241.92	21.80 %	2,500.00 pg/mL	2,325.27 pg/mL	93.01 %

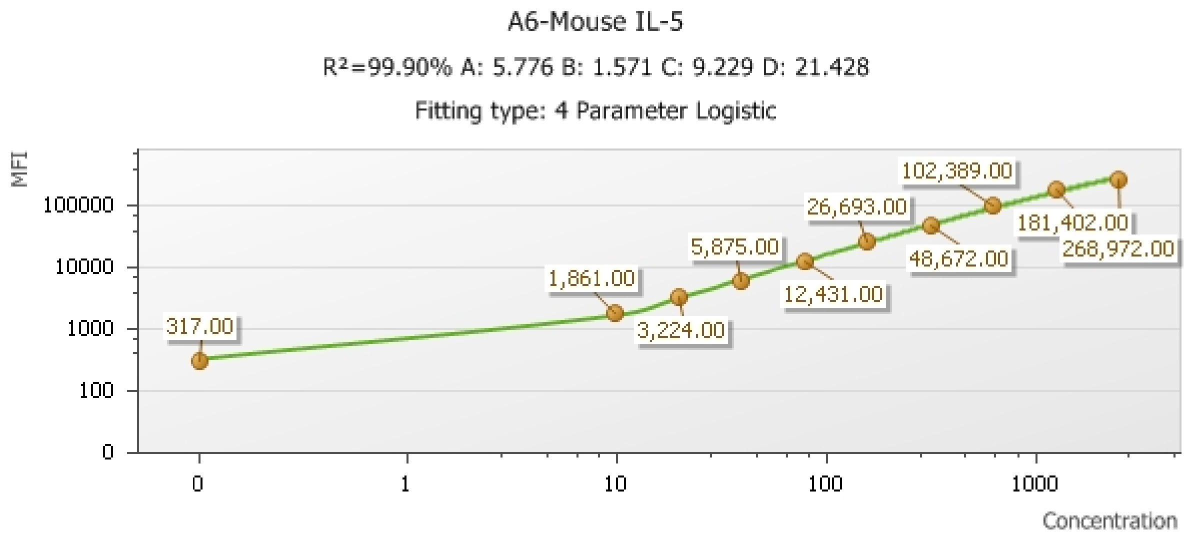

Name	Event #	MFI	SD	CV% (MFI)	Nominal CC	Fitted CC	Recovery %
Std001	1134	317.00	318.20	74.38 %	0.00 pg/mL	0.00 pg/mL	0.00 %
Std002	1206	1,861.00	636.78	34.00 %	9.77 pg/mL	10.81 pg/mL	110.69 %
Std003	1284	3,224.00	1,063.58	34.82 %	19.53 pg/mL	19.36 pg/mL	99.12 %
Std004	1003	5,875.00	1,773.93	32.16 %	39.06 pg/mL	35.46 pg/mL	90.78 %
Std005	1222	12,431.00	3,786.75	29.67 %	78.13 pg/mL	74.67 pg/mL	95.58 %
Std006	1061	26,693.00	7,511.22	33.51 %	156.25 pg/mL	161.69 pg/mL	103.48 %
Std007	1021	48,672.00	16,672.58	38.09 %	312.50 pg/mL	303.67 pg/mL	97.18 %

Name	Event #	MFI	SD	CV% (MFI)	Nominal CC	Fitted CC	Recovery %
Std008	1062	102,389.00	29,269.30	26.16 %	625.00 pg/mL	691.62 pg/mL	110.66 %
Std009	960	181,402.00	43,759.87	23.59 %	1,250.00 pg/mL	1,359.15 pg/mL	108.73 %
Std010	955	268,972.00	67,804.49	22.47 %	2,500.00 pg/mL	2,222.90 pg/mL	88.92 %

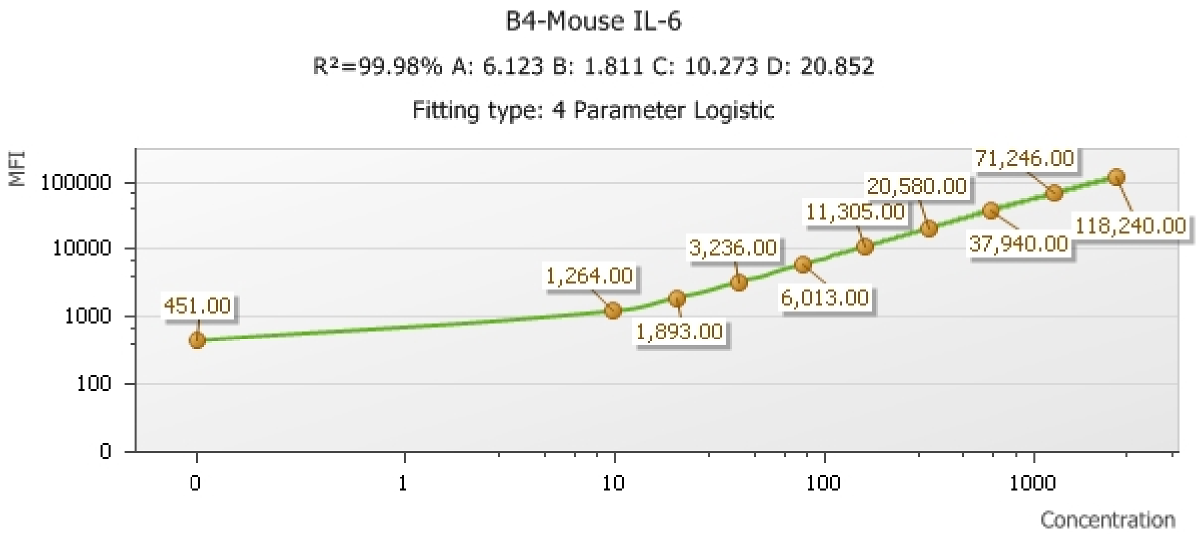

Name	Event #	MFI	SD	CV% (MFI)	Nominal CC	Fitted CC	Recovery %
Std001	902	451.00	301.15	61.67 %	0.00 pg/mL	0.00 pg/mL	0.00 %
Std002	873	1,264.00	440.33	41.12 %	9.77 pg/mL	10.55 pg/mL	108.07 %
Std003	1221	1,893.00	659.39	35.48 %	19.53 pg/mL	18.91 pg/mL	96.79 %
Std004	869	3,236.00	994.45	36.07 %	39.06 pg/mL	37.48 pg/mL	95.96 %
Std005	1220	6,013.00	1,799.32	31.55 %	78.13 pg/mL	77.75 pg/mL	99.52 %
Std006	976	11,305.00	3,269.50	34.33 %	156.25 pg/mL	158.90 pg/mL	101.70 %
Std007	886	20,580.00	6,985.83	35.44 %	312.50 pg/mL	311.34 pg/mL	99.63 %
Std008	961	37,940.00	9,915.26	32.95 %	625.00 pg/mL	624.08 pg/mL	99.85 %

Name	Event #	MFI	SD	CV% (MFI)	Nominal CC	Fitted CC	Recovery %
Std009	873	71,246.00	20,442.83	37.29 %	1,250.00 pg/mL	1,305.11 pg/mL	104.41 %
Std010	887	118,240.00	32,324.39	33.98 %	2,500.00 pg/mL	2,418.58 pg/mL	96.74 %

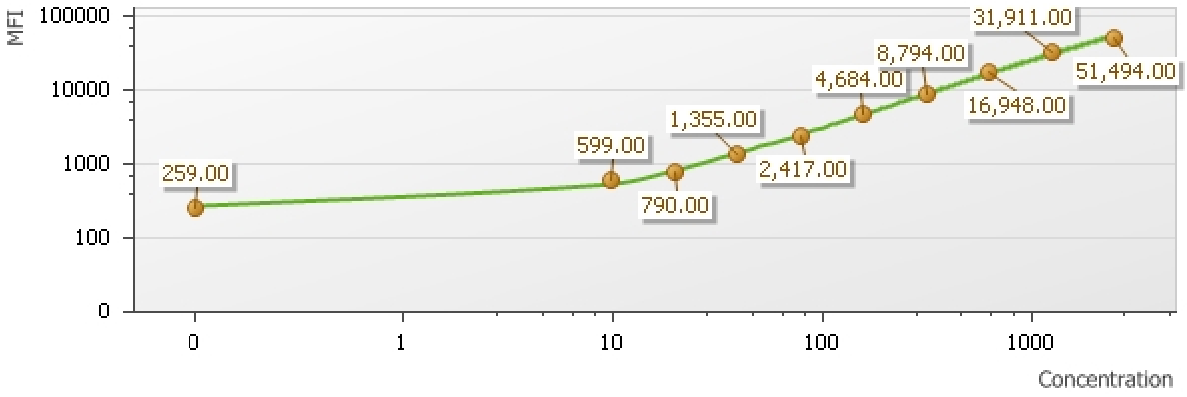

Name	Event #	MFI	SD	CV% (MFI)	Nominal CC	Fitted CC	Recovery %
Std001	802	259.00	284.84	82.65 %	0.00 pg/mL	0.00 pg/mL	0.00 %
Std002	784	599.00	318.76	49.33 %	9.77 pg/mL	11.71 pg/mL	119.88 %
Std003	1037	790.00	390.29	47.24 %	19.53 pg/mL	18.17 pg/mL	93.03 %
Std004	883	1,355.00	424.02	29.18 %	39.06 pg/mL	37.67 pg/mL	96.44 %
Std005	1000	2,417.00	702.57	26.85 %	78.13 pg/mL	75.09 pg/mL	96.11 %
Std006	860	4,684.00	1,276.15	22.90 %	156.25 pg/mL	157.07 pg/mL	100.52 %
Std007	827	8,794.00	2,652.37	24.46 %	312.50 pg/mL	312.23 pg/mL	99.91 %
Std008	875	16,948.00	4,808.81	22.98 %	625.00 pg/mL	643.54 pg/mL	102.97 %
Std009	847	31,911.00	8,123.17	21.86 %	1,250.00 pg/mL	1,326.43 pg/mL	106.11 %

Name	Event #	MFI	SD	CV% (MFI)	Nominal CC	Fitted CC	Recovery %
Std010	903	51,494.00	15,497.62	25.15 %	2,500.00 pg/mL	2,355.74 pg/mL	94.23 %

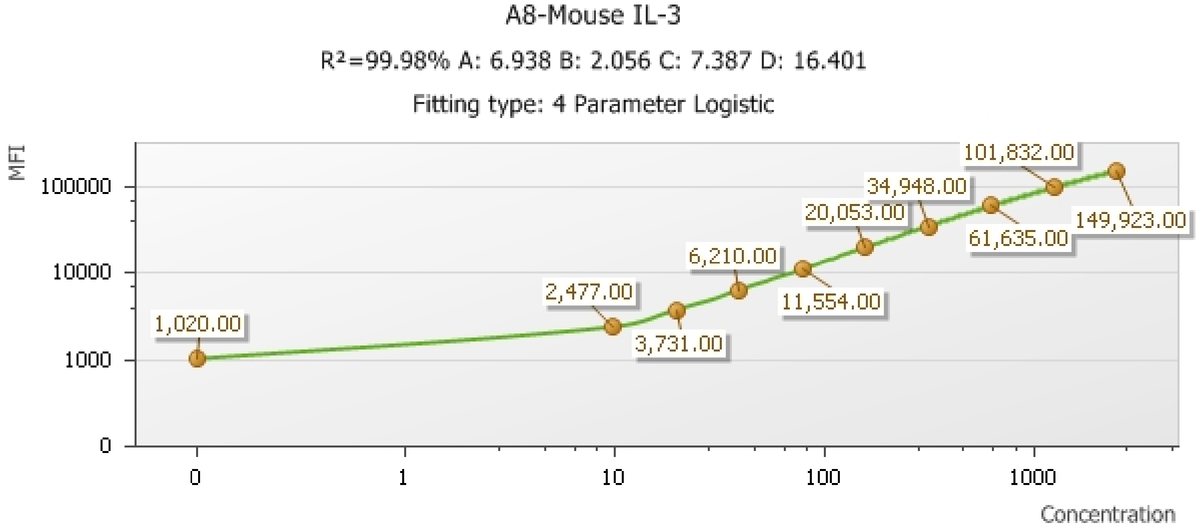

Name	Event #	MFI	SD	CV% (MFI)	Nominal CC	Fitted CC	Recovery %
Std001	785	1,020.00	339.52	30.24 %	0.00 pg/mL	0.00 pg/mL	0.00 %
Std002	865	2,477.00	670.51	24.21 %	9.77 pg/mL	10.42 pg/mL	106.71 %
Std003	1147	3,731.00	965.17	24.53 %	19.53 pg/mL	19.19 pg/mL	98.24 %
Std004	844	6,210.00	1,660.33	24.33 %	39.06 pg/mL	37.35 pg/mL	95.61 %
Std005	1053	11,554.00	3,057.86	27.08 %	78.13 pg/mL	79.63 pg/mL	101.92 %
Std006	848	20,053.00	5,394.07	23.14 %	156.25 pg/mL	154.61 pg/mL	98.95 %
Std007	797	34,948.00	10,550.92	24.18 %	312.50 pg/mL	307.07 pg/mL	98.26 %
Std008	910	61,635.00	18,666.68	25.97 %	625.00 pg/mL	644.65 pg/mL	103.14 %
Std009	835	101,832.00	30,522.29	23.80 %	1,250.00 pg/mL	1,311.37 pg/mL	104.91 %
Std010	744	149,923.00	43,014.49	22.43 %	2,500.00 pg/mL	2,377.53 pg/mL	95.10 %

Results Statistics for A4 - Mouse IFN-γ

Name	Plate Pos.	Clust.	Event #	MFI	SD	CV	Dilution
Std001	1 - A1	Manual	1670	365.00	293.55	68.58 %	1.00
Std002	1 - A2	Manual	1541	2,012.00	828.03	39.22 %	1.00
Std003	1 - A3	Manual	1865	3,638.00	1,363.25	39.58 %	1.00
Std004	1 - A4	Manual	1249	6,521.00	2,396.25	35.94 %	1.00
Std005	1 - A5	Manual	1482	12,788.00	3,674.62	32.79 %	1.00
Std006	1 - A6	Manual	1640	23,795.00	9,441.38	38.43 %	1.00
Std007	1 - A7	Manual	1534	41,975.00	16,643.11	38.86 %	1.00
Std008	1 - A8	Manual	1630	82,377.00	34,350.17	37.71 %	1.00
Std009	1 - A9	Manual	1499	148,479.00	59,269.90	37.41 %	1.00
Std010	1 - A10	Manual	1514	227,760.00	81,553.38	34.97 %	1.00
V1B	1 - B1	Manual	1790	359.00	301.15	72.42 %	1.00
A1B	1 - B2	Manual	1499	258.00	318.02	85.19 %	1.00
139 1 B	1 - B3	Manual	1362	262.00	356.01	88.05 %	1.00
224 1 B	1 - B4	Manual	1513	319.00	298.74	77.03 %	1.00
164 1 B	1 - B5	Manual	1422	335.00	319.87	74.61 %	1.00
V1A	1 - C1	Manual	1665	325.00	299.49	72.91 %	1.00
A1A	1 - C2	Manual	1901	324.00	301.71	74.35 %	1.00
139 1 A	1 - C3	Manual	1731	336.00	295.78	72.74 %	1.00
224 1 A	1 - C4	Manual	1525	325.00	295.41	73.67 %	1.00
164 1 A	1 - C5	Manual	1161	399.00	236.10	58.34 %	1.00
untreated	1 - D1	Manual	992	220.00	293.37	87.14 %	1.00

Quantitative Analyte Results for A4 - Mouse IFN-γ

Name	Plate Position	Nominal CC	Fitted CC	Final CC	Message
Std001	1 - A1	0.00 pg/mL	0.00 pg/mL	0.00 pg/mL	Fitting: Below standard range and out of invertable range, Recovery: Out of range
Std002	1 - A2	9.77 pg/mL	10.26 pg/mL	10.26 pg/mL	
Std003	1 - A3	19.53 pg/mL	20.05 pg/mL	20.05 pg/mL	
Std004	1 - A4	39.06 pg/mL	37.68 pg/mL	37.68 pg/mL	
Std005	1 - A5	78.13 pg/mL	77.60 pg/mL	77.60 pg/mL	

Quantitative Analyte Results for A4 - Mouse IFN-γ

Name	Plate Position	Nominal CC	Fitted CC	Final CC	Message
Std006	1 - A6	156.25 pg/mL	152.95 pg/mL	152.95 pg/mL	
Std007	1 - A7	312.50 pg/mL	290.53 pg/mL	290.53 pg/mL	
Std008	1 - A8	625.00 pg/mL	647.11 pg/mL	647.11 pg/mL	
Std009	1 - A9	1,250.00 pg/mL	1,362.62 pg/mL	1,362.62 pg/mL	
Std010	1 - A10	2,500.00 pg/mL	2,417.33 pg/mL	2,417.33 pg/mL	
V1B	1 - B1	N/A	0.00 pg/mL	0.00 pg/mL	Fitting: Below standard range and out of invertable range
A1B	1 - B2	N/A	0.00 pg/mL	0.00 pg/mL	Fitting: Below standard range and out of invertable range
139 1 B	1 - B3	N/A	0.00 pg/mL	0.00 pg/mL	Fitting: Below standard range and out of invertable range
224 1 B	1 - B4	N/A	0.00 pg/mL	0.00 pg/mL	Fitting: Below standard range and out of invertable range
164 1 B	1 - B5	N/A	0.00 pg/mL	0.00 pg/mL	Fitting: Below standard range and out of invertable range
V1A	1 - C1	N/A	0.00 pg/mL	0.00 pg/mL	Fitting: Below standard range and out of invertable range
A1A	1 - C2	N/A	0.00 pg/mL	0.00 pg/mL	Fitting: Below standard range and out of invertable range
139 1 A	1 - C3	N/A	0.00 pg/mL	0.00 pg/mL	Fitting: Below standard range and out of invertable range
224 1 A	1 - C4	N/A	0.00 pg/mL	0.00 pg/mL	Fitting: Below standard range and out of invertable range
164 1 A	1 - C5	N/A	0.31 pg/mL	0.31 pg/mL	

Quantitative Analyte Results for A4 - Mouse IFN-γ

Name	Plate Position	Nominal CC	Fitted CC	Final CC	Message
untreated	1 - D1	N/A	0.00 pg/mL	0.00 pg/mL	Fitting: Below standard range and out of invertable range

Results Statistics for C4 - Mouse IL-10

Name	Plate Pos.	Clust.	Event #	MFI	SD	CV	Dilution
Std001	1 - A1	Manual	788	297.00	311.72	80.14 %	1.00
Std002	1 - A2	Manual	962	436.00	300.23	62.77 %	1.00
Std003	1 - A3	Manual	983	525.00	401.04	65.25 %	1.00
Std004	1 - A4	Manual	976	839.00	318.02	38.22 %	1.00
Std005	1 - A5	Manual	1063	1,373.00	471.47	31.63 %	1.00
Std006	1 - A6	Manual	792	2,387.00	646.04	27.01 %	1.00
Std007	1 - A7	Manual	876	4,340.00	1,295.79	25.45 %	1.00
Std008	1 - A8	Manual	872	8,120.00	2,226.49	25.42 %	1.00
Std009	1 - A9	Manual	897	15,434.00	4,477.45	25.75 %	1.00
Std010	1 - A10	Manual	916	26,081.00	7,289.76	24.98 %	1.00
V1B	1 - B1	Manual	977	282.00	308.38	81.63 %	1.00
A1B	1 - B2	Manual	751	224.00	312.09	89.92 %	1.00
139 1 B	1 - B3	Manual	745	185.00	286.88	95.37 %	1.00
224 1 B	1 - B4	Manual	794	213.00	323.58	90.66 %	1.00
164 1 B	1 - B5	Manual	774	266.00	278.73	80.05 %	1.00
V1A	1 - C1	Manual	996	243.00	287.07	84.75 %	1.00
A1A	1 - C2	Manual	1071	254.00	302.45	83.54 %	1.00
139 1 A	1 - C3	Manual	936	285.00	297.63	80.47 %	1.00
224 1 A	1 - C4	Manual	1042	260.00	292.81	79.66 %	1.00
164 1 A	1 - C5	Manual	911	316.00	243.89	66.98 %	1.00
untreated	1 - D1	Manual	880	223.00	303.19	90.65 %	1.00

Quantitative Analyte Results for C4 - Mouse IL-10

Name	Plate Position	Nominal CC	Fitted CC	Final CC	Message
Std001	1 - A1	0.00 pg/mL	0.00 pg/mL	0.00 pg/mL	Fitting: Below standard range and out of invertable range, Recovery: Out of range
Std002	1 - A2	9.77 pg/mL	11.02 pg/mL	11.02 pg/mL	
Std003	1 - A3	19.53 pg/mL	17.32 pg/mL	17.32 pg/mL	
Std004	1 - A4	39.06 pg/mL	39.98 pg/mL	39.98 pg/mL	
Std005	1 - A5	78.13 pg/mL	79.40 pg/mL	79.40 pg/mL	
Std006	1 - A6	156.25 pg/mL	155.76 pg/mL	155.76 pg/mL	
Std007	1 - A7	312.50 pg/mL	307.43 pg/mL	307.43 pg/mL	
Std008	1 - A8	625.00 pg/mL	619.30 pg/mL	619.30 pg/mL	
Std009	1 - A9	1,250.00 pg/mL	1,295.10 pg/mL	1,295.10 pg/mL	
Std010	1 - A10	2,500.00 pg/mL	2,454.55 pg/mL	2,454.55 pg/mL	
V1B	1 - B1	N/A	0.00 pg/mL	0.00 pg/mL	Fitting: Below standard range and out of invertable range
A1B	1 - B2	N/A	0.00 pg/mL	0.00 pg/mL	Fitting: Below standard range and out of invertable range
139 1 B	1 - B3	N/A	0.00 pg/mL	0.00 pg/mL	Fitting: Below standard range and out of invertable range
224 1 B	1 - B4	N/A	0.00 pg/mL	0.00 pg/mL	Fitting: Below standard range and out of invertable range
164 1 B	1 - B5	N/A	0.00 pg/mL	0.00 pg/mL	Fitting: Below standard range and out of invertable range
V1A	1 - C1	N/A	0.00 pg/mL	0.00 pg/mL	Fitting: Below standard range and out of invertable range
A1A	1 - C2	N/A	0.00 pg/mL	0.00 pg/mL	Fitting: Below standard range and out of invertable range

Quantitative Analyte Results for C4 - Mouse IL-10

Name	Plate Position	Nominal CC	Fitted CC	Final CC	Message
139 1 A	1 - C3	N/A	0.00 pg/mL	0.00 pg/mL	Fitting: Below standard range and out of invertable range
224 1 A	1 - C4	N/A	0.00 pg/mL	0.00 pg/mL	Fitting: Below standard range and out of invertable range
164 1 A	1 - C5	N/A	2.24 pg/mL	2.24 pg/mL	
untreated	1 - D1	N/A	0.00 pg/mL	0.00 pg/mL	Fitting: Below standard range and out of invertable range

Results Statistics for D7 - Mouse IL-12p70

Name	Plate Pos.	Clust.	Event #	MFI	SD	CV	Dilution
Std001	1 - A1	Manual	685	230.00	323.21	89.97 %	1.00
Std002	1 - A2	Manual	720	485.00	285.96	57.04 %	1.00
Std003	1 - A3	Manual	882	610.00	368.80	57.84 %	1.00
Std004	1 - A4	Manual	600	1,075.00	365.46	32.28 %	1.00
Std005	1 - A5	Manual	786	1,772.00	537.26	27.36 %	1.00
Std006	1 - A6	Manual	737	3,168.00	691.63	20.22 %	1.00
Std007	1 - A7	Manual	664	5,601.00	1,243.53	19.75 %	1.00
Std008	1 - A8	Manual	686	10,519.00	2,456.67	18.73 %	1.00
Std009	1 - A9	Manual	633	18,882.00	3,123.10	17.72 %	1.00
Std010	1 - A10	Manual	636	30,522.00	4,574.75	15.90 %	1.00
V1B	1 - B1	Manual	701	251.00	306.16	85.72 %	1.00
A1B	1 - B2	Manual	547	168.00	272.80	100.36 %	1.00
139 1 B	1 - B3	Manual	524	80.00	201.08	114.34 %	1.00
224 1 B	1 - B4	Manual	528	191.00	279.28	90.77 %	1.00
164 1 B	1 - B5	Manual	597	196.00	307.64	89.64 %	1.00
V1A	1 - C1	Manual	696	248.00	287.44	81.88 %	1.00
A1A	1 - C2	Manual	930	179.00	265.57	92.75 %	1.00
139 1 A	1 - C3	Manual	826	214.00	267.05	86.93 %	1.00
224 1 A	1 - C4	Manual	971	239.00	275.02	82.58 %	1.00
164 1 A	1 - C5	Manual	1058	298.00	231.66	69.36 %	1.00
untreated	1 - D1	Manual	555	110.00	219.42	107.32 %	1.00

Quantitative Analyte Results for D7 - Mouse IL-12p70

Name	Plate Position	Nominal CC	Fitted CC	Final CC	Message
Std001	1 - A1	0.00 pg/mL	0.00 pg/mL	0.00 pg/mL	Fitting: Below standard range and out of invertable range, Recovery: Out of range
Std002	1 - A2	9.77 pg/mL	11.42 pg/mL	11.42 pg/mL	
Std003	1 - A3	19.53 pg/mL	17.17 pg/mL	17.17 pg/mL	
Std004	1 - A4	39.06 pg/mL	40.01 pg/mL	40.01 pg/mL	
Std005	1 - A5	78.13 pg/mL	76.74 pg/mL	76.74 pg/mL	
Std006	1 - A6	156.25 pg/mL	155.73 pg/mL	155.73 pg/mL	
Std007	1 - A7	312.50 pg/mL	305.40 pg/mL	305.40 pg/mL	
Std008	1 - A8	625.00 pg/mL	643.50 pg/mL	643.50 pg/mL	
Std009	1 - A9	1,250.00 pg/mL	1,308.28 pg/mL	1,308.28 pg/mL	
Std010	1 - A10	2,500.00 pg/mL	2,398.14 pg/mL	2,398.14 pg/mL	
V1B	1 - B1	N/A	1.20 pg/mL	1.20 pg/mL	
A1B	1 - B2	N/A	0.00 pg/mL	0.00 pg/mL	Fitting: Below standard range and out of invertable range
139 1 B	1 - B3	N/A	0.00 pg/mL	0.00 pg/mL	Fitting: Below standard range and out of invertable range
224 1 B	1 - B4	N/A	0.00 pg/mL	0.00 pg/mL	Fitting: Below standard range and out of invertable range
164 1 B	1 - B5	N/A	0.00 pg/mL	0.00 pg/mL	Fitting: Below standard range and out of invertable range
V1A	1 - C1	N/A	1.05 pg/mL	1.05 pg/mL	
A1A	1 - C2	N/A	0.00 pg/mL	0.00 pg/mL	Fitting: Below standard range and out of invertable range
139 1 A	1 - C3	N/A	0.00 pg/mL	0.00 pg/mL	Fitting: Below standard range and out of invertable range

Quantitative Analyte Results for D7 - Mouse IL-12p70

Name	Plate Position	Nominal CC	Fitted CC	Final CC	Message
224 1 A	1 - C4	N/A	0.52 pg/mL	0.52 pg/mL	
164 1 A	1 - C5	N/A	3.27 pg/mL	3.27 pg/mL	
untreated	1 - D1	N/A	0.00 pg/mL	0.00 pg/mL	Fitting: Below standard range and out of invertable range

Results Statistics for A5 - Mouse IL-2

Name	Plate Pos.	Clust.	Event #	MFI	SD	CV	Dilution
Std001	1 - A1	Manual	794	352.00	295.22	68.12 %	1.00
Std002	1 - A2	Manual	887	2,319.00	646.41	27.16 %	1.00
Std003	1 - A3	Manual	1010	4,195.00	1,117.88	24.25 %	1.00
Std004	1 - A4	Manual	853	7,975.00	2,192.39	24.47 %	1.00
Std005	1 - A5	Manual	989	16,051.00	4,491.54	26.10 %	1.00
Std006	1 - A6	Manual	830	32,582.00	8,557.01	22.00 %	1.00
Std007	1 - A7	Manual	747	60,588.00	16,679.25	22.49 %	1.00
Std008	1 - A8	Manual	757	106,038.00	27,922.92	20.84 %	1.00
Std009	1 - A9	Manual	687	168,882.00	45,437.98	20.61 %	1.00
Std010	1 - A10	Manual	692	212,558.00	56,941.48	20.37 %	1.00
V1B	1 - B1	Manual	770	378.00	291.70	68.42 %	1.00
A1B	1 - B2	Manual	741	301.00	358.79	84.57 %	1.00
139 1 B	1 - B3	Manual	723	275.00	338.03	84.93 %	1.00
224 1 B	1 - B4	Manual	855	348.00	325.43	75.16 %	1.00
164 1 B	1 - B5	Manual	779	357.00	286.14	69.69 %	1.00
V1A	1 - C1	Manual	1066	314.00	293.18	75.50 %	1.00
A1A	1 - C2	Manual	958	301.00	312.64	76.51 %	1.00
139 1 A	1 - C3	Manual	853	336.00	292.44	70.99 %	1.00
224 1 A	1 - C4	Manual	1020	349.00	282.81	68.39 %	1.00
164 1 A	1 - C5	Manual	848	408.00	228.69	56.44 %	1.00
untreated	1 - D1	Manual	777	306.00	301.34	78.28 %	1.00

Quantitative Analyte Results for A5 - Mouse IL-2

Name	Plate Position	Nominal CC	Fitted CC	Final CC	Message
Std001	1 - A1	0.00 pg/mL	0.00 pg/mL	0.00 pg/mL	Fitting: Below standard range and out of invertable range, Recovery: Out of range
Std002	1 - A2	9.77 pg/mL	10.70 pg/mL	10.70 pg/mL	
Std003	1 - A3	19.53 pg/mL	19.27 pg/mL	19.27 pg/mL	
Std004	1 - A4	39.06 pg/mL	36.08 pg/mL	36.08 pg/mL	
Std005	1 - A5	78.13 pg/mL	72.95 pg/mL	72.95 pg/mL	
Std006	1 - A6	156.25 pg/mL	156.93 pg/mL	156.93 pg/mL	
Std007	1 - A7	312.50 pg/mL	329.43 pg/mL	329.43 pg/mL	
Std008	1 - A8	625.00 pg/mL	697.11 pg/mL	697.11 pg/mL	
Std009	1 - A9	1,250.00 pg/mL	1,406.33 pg/mL	1,406.33 pg/mL	
Std010	1 - A10	2,500.00 pg/mL	2,056.59 pg/mL	2,056.59 pg/mL	
V1B	1 - B1	N/A	0.32 pg/mL	0.32 pg/mL	
A1B	1 - B2	N/A	0.00 pg/mL	0.00 pg/mL	Fitting: Below standard range and out of invertable range
139 1 B	1 - B3	N/A	0.00 pg/mL	0.00 pg/mL	Fitting: Below standard range and out of invertable range
224 1 B	1 - B4	N/A	0.00 pg/mL	0.00 pg/mL	Fitting: Below standard range and out of invertable range
164 1 B	1 - B5	N/A	0.00 pg/mL	0.00 pg/mL	Fitting: Out of invertable range
V1A	1 - C1	N/A	0.00 pg/mL	0.00 pg/mL	Fitting: Below standard range and out of invertable range
A1A	1 - C2	N/A	0.00 pg/mL	0.00 pg/mL	Fitting: Below standard range and out of invertable range

Quantitative Analyte Results for A5 - Mouse IL-2

Name	Plate Position	Nominal CC	Fitted CC	Final CC	Message
139 1 A	1 - C3	N/A	0.00 pg/mL	0.00 pg/mL	Fitting: Below standard range and out of invertable range
224 1 A	1 - C4	N/A	0.00 pg/mL	0.00 pg/mL	Fitting: Below standard range and out of invertable range
164 1 A	1 - C5	N/A	0.59 pg/mL	0.59 pg/mL	
untreated	1 - D1	N/A	0.00 pg/mL	0.00 pg/mL	Fitting: Below standard range and out of invertable range

Results Statistics for A7 - Mouse IL-4

Name	Plate Pos.	Clust.	Event #	MFI	SD	CV	Dilution
Std001	1 - A1	Manual	1192	387.00	308.38	68.15 %	1.00
Std002	1 - A2	Manual	1122	1,791.00	517.80	26.82 %	1.00
Std003	1 - A3	Manual	1552	3,035.00	922.55	33.82 %	1.00
Std004	1 - A4	Manual	1113	5,912.00	1,879.20	33.83 %	1.00
Std005	1 - A5	Manual	1317	12,572.00	3,953.72	32.32 %	1.00
Std006	1 - A6	Manual	937	27,271.00	7,743.62	24.66 %	1.00
Std007	1 - A7	Manual	899	50,332.00	15,998.00	27.42 %	1.00
Std008	1 - A8	Manual	1100	100,178.00	30,306.20	30.12 %	1.00
Std009	1 - A9	Manual	921	179,092.00	53,263.89	26.87 %	1.00
Std010	1 - A10	Manual	905	278,285.00	73,241.92	21.80 %	1.00
V1B	1 - B1	Manual	1028	351.00	288.37	69.62 %	1.00
A1B	1 - B2	Manual	906	292.00	332.29	79.84 %	1.00
139 1 B	1 - B3	Manual	847	261.00	331.36	86.87 %	1.00
224 1 B	1 - B4	Manual	972	332.00	303.01	73.48 %	1.00
164 1 B	1 - B5	Manual	929	349.00	310.60	70.30 %	1.00
V1A	1 - C1	Manual	1173	304.00	277.62	73.17 %	1.00
A1A	1 - C2	Manual	1311	317.00	283.18	73.07 %	1.00
139 1 A	1 - C3	Manual	1225	323.00	280.58	71.27 %	1.00
224 1 A	1 - C4	Manual	1174	330.00	282.99	70.09 %	1.00
164 1 A	1 - C5	Manual	1174	426.00	250.93	55.77 %	1.00
untreated	1 - D1	Manual	809	271.00	298.37	82.27 %	1.00

Quantitative Analyte Results for A7 - Mouse IL-4

Name	Plate Position	Nominal CC	Fitted CC	Final CC	Message
Std001	1 - A1	0.00 pg/mL	0.00 pg/mL	0.00 pg/mL	Fitting: Below standard range and out of invertable range, Recovery: Out of range
Std002	1 - A2	9.77 pg/mL	10.75 pg/mL	10.75 pg/mL	
Std003	1 - A3	19.53 pg/mL	18.88 pg/mL	18.88 pg/mL	
Std004	1 - A4	39.06 pg/mL	36.61 pg/mL	36.61 pg/mL	
Std005	1 - A5	78.13 pg/mL	76.02 pg/mL	76.02 pg/mL	
Std006	1 - A6	156.25 pg/mL	163.18 pg/mL	163.18 pg/mL	
Std007	1 - A7	312.50 pg/mL	307.29 pg/mL	307.29 pg/mL	
Std008	1 - A8	625.00 pg/mL	656.26 pg/mL	656.26 pg/mL	
Std009	1 - A9	1,250.00 pg/mL	1,314.65 pg/mL	1,314.65 pg/mL	
Std010	1 - A10	2,500.00 pg/mL	2,325.27 pg/mL	2,325.27 pg/mL	
V1B	1 - B1	N/A	0.00 pg/mL	0.00 pg/mL	Fitting: Below standard range and out of invertable range
A1B	1 - B2	N/A	0.00 pg/mL	0.00 pg/mL	Fitting: Below standard range and out of invertable range
139 1 B	1 - B3	N/A	0.00 pg/mL	0.00 pg/mL	Fitting: Below standard range and out of invertable range
224 1 B	1 - B4	N/A	0.00 pg/mL	0.00 pg/mL	Fitting: Below standard range and out of invertable range
164 1 B	1 - B5	N/A	0.00 pg/mL	0.00 pg/mL	Fitting: Below standard range and out of invertable range
V1A	1 - C1	N/A	0.00 pg/mL	0.00 pg/mL	Fitting: Below standard range and out of invertable range
A1A	1 - C2	N/A	0.00 pg/mL	0.00 pg/mL	Fitting: Below standard range and out of invertable range

Quantitative Analyte Results for A7 - Mouse IL-4

Name	Plate Position	Nominal CC	Fitted CC	Final CC	Message
139 1 A	1 - C3	N/A	0.00 pg/mL	0.00 pg/mL	Fitting: Below standard range and out of invertable range
224 1 A	1 - C4	N/A	0.00 pg/mL	0.00 pg/mL	Fitting: Below standard range and out of invertable range
164 1 A	1 - C5	N/A	0.56 pg/mL	0.56 pg/mL	
untreated	1 - D1	N/A	0.00 pg/mL	0.00 pg/mL	Fitting: Below standard range and out of invertable range

Results Statistics for A6 - Mouse IL-5

Name	Plate Pos.	Clust.	Event #	MFI	SD	CV	Dilution
Std001	1 - A1	Manual	1134	317.00	318.20	74.38 %	1.00
Std002	1 - A2	Manual	1206	1,861.00	636.78	34.00 %	1.00
Std003	1 - A3	Manual	1284	3,224.00	1,063.58	34.82 %	1.00
Std004	1 - A4	Manual	1003	5,875.00	1,773.93	32.16 %	1.00
Std005	1 - A5	Manual	1222	12,431.00	3,786.75	29.67 %	1.00
Std006	1 - A6	Manual	1061	26,693.00	7,511.22	33.51 %	1.00
Std007	1 - A7	Manual	1021	48,672.00	16,672.58	38.09 %	1.00
Std008	1 - A8	Manual	1062	102,389.00	29,269.30	26.16 %	1.00
Std009	1 - A9	Manual	960	181,402.00	43,759.87	23.59 %	1.00
Std010	1 - A10	Manual	955	268,972.00	67,804.49	22.47 %	1.00
V1B	1 - B1	Manual	1215	299.00	281.69	75.37 %	1.00
A1B	1 - B2	Manual	1030	243.00	339.14	88.09 %	1.00
139 1 B	1 - B3	Manual	925	211.00	330.99	94.01 %	1.00
224 1 B	1 - B4	Manual	1186	266.00	316.16	83.60 %	1.00
164 1 B	1 - B5	Manual	1032	303.00	305.97	77.06 %	1.00
V1A	1 - C1	Manual	1607	282.00	268.35	77.68 %	1.00
A1A	1 - C2	Manual	1526	266.00	283.92	79.26 %	1.00
139 1 A	1 - C3	Manual	1340	290.00	283.73	76.59 %	1.00
224 1 A	1 - C4	Manual	1555	306.00	283.18	74.72 %	1.00
164 1 A	1 - C5	Manual	1371	357.00	246.11	61.88 %	1.00
untreated	1 - D1	Manual	951	241.00	293.55	86.11 %	1.00

Quantitative Analyte Results for A6 - Mouse IL-5

Name	Plate Position	Nominal CC	Fitted CC	Final CC	Message
Std001	1 - A1	0.00 pg/mL	0.00 pg/mL	0.00 pg/mL	Fitting: Below standard range and out of invertable range, Recovery: Out of range
Std002	1 - A2	9.77 pg/mL	10.81 pg/mL	10.81 pg/mL	
Std003	1 - A3	19.53 pg/mL	19.36 pg/mL	19.36 pg/mL	
Std004	1 - A4	39.06 pg/mL	35.46 pg/mL	35.46 pg/mL	
Std005	1 - A5	78.13 pg/mL	74.67 pg/mL	74.67 pg/mL	
Std006	1 - A6	156.25 pg/mL	161.69 pg/mL	161.69 pg/mL	
Std007	1 - A7	312.50 pg/mL	303.67 pg/mL	303.67 pg/mL	
Std008	1 - A8	625.00 pg/mL	691.62 pg/mL	691.62 pg/mL	
Std009	1 - A9	1,250.00 pg/mL	1,359.15 pg/mL	1,359.15 pg/mL	
Std010	1 - A10	2,500.00 pg/mL	2,222.90 pg/mL	2,222.90 pg/mL	
V1B	1 - B1	N/A	0.00 pg/mL	0.00 pg/mL	Fitting: Below standard range and out of invertable range
A1B	1 - B2	N/A	0.00 pg/mL	0.00 pg/mL	Fitting: Below standard range and out of invertable range
139 1 B	1 - B3	N/A	0.00 pg/mL	0.00 pg/mL	Fitting: Below standard range and out of invertable range
224 1 B	1 - B4	N/A	0.00 pg/mL	0.00 pg/mL	Fitting: Below standard range and out of invertable range
164 1 B	1 - B5	N/A	0.00 pg/mL	0.00 pg/mL	Fitting: Below standard range and out of invertable range
V1A	1 - C1	N/A	0.00 pg/mL	0.00 pg/mL	Fitting: Below standard range and out of invertable range
A1A	1 - C2	N/A	0.00 pg/mL	0.00 pg/mL	Fitting: Below standard range and out of invertable range

Quantitative Analyte Results for A6 - Mouse IL-5

Name	Plate Position	Nominal CC	Fitted CC	Final CC	Message
139 1 A	1 - C3	N/A	0.00 pg/mL	0.00 pg/mL	Fitting: Below standard range and out of invertable range
224 1 A	1 - C4	N/A	0.00 pg/mL	0.00 pg/mL	Fitting: Below standard range and out of invertable range
164 1 A	1 - C5	N/A	0.45 pg/mL	0.45 pg/mL	
untreated	1 - D1	N/A	0.00 pg/mL	0.00 pg/mL	Fitting: Below standard range and out of invertable range

Results Statistics for B4 - Mouse IL-6

Name	Plate Pos.	Clust.	Event #	MFI	SD	CV	Dilution
Std001	1 - A1	Manual	902	451.00	301.15	61.67 %	1.00
Std002	1 - A2	Manual	873	1,264.00	440.33	41.12 %	1.00
Std003	1 - A3	Manual	1221	1,893.00	659.39	35.48 %	1.00
Std004	1 - A4	Manual	869	3,236.00	994.45	36.07 %	1.00
Std005	1 - A5	Manual	1220	6,013.00	1,799.32	31.55 %	1.00
Std006	1 - A6	Manual	976	11,305.00	3,269.50	34.33 %	1.00
Std007	1 - A7	Manual	886	20,580.00	6,985.83	35.44 %	1.00
Std008	1 - A8	Manual	961	37,940.00	9,915.26	32.95 %	1.00
Std009	1 - A9	Manual	873	71,246.00	20,442.83	37.29 %	1.00
Std010	1 - A10	Manual	887	118,240.00	32,324.39	33.98 %	1.00
V1B	1 - B1	Manual	962	422.00	318.57	64.32 %	1.00
A1B	1 - B2	Manual	838	391.00	353.97	73.71 %	1.00
139 1 B	1 - B3	Manual	737	400.00	373.62	78.26 %	1.00
224 1 B	1 - B4	Manual	880	399.00	352.12	73.43 %	1.00
164 1 B	1 - B5	Manual	813	414.00	315.05	68.31 %	1.00
V1A	1 - C1	Manual	948	413.00	319.50	68.05 %	1.00
A1A	1 - C2	Manual	1202	408.00	317.83	67.53 %	1.00
139 1 A	1 - C3	Manual	1009	394.00	312.09	67.90 %	1.00
224 1 A	1 - C4	Manual	1036	445.00	292.26	61.79 %	1.00
164 1 A	1 - C5	Manual	1371	454.00	259.46	53.22 %	1.00
untreated	1 - D1	Manual	997	422.00	390.29	72.36 %	1.00

Quantitative Analyte Results for B4 - Mouse IL-6

Name	Plate Position	Nominal CC	Fitted CC	Final CC	Message
Std001	1 - A1	0.00 pg/mL	0.00 pg/mL	0.00 pg/mL	Fitting: Below standard range and out of invertable range, Recovery: Out of range
Std002	1 - A2	9.77 pg/mL	10.55 pg/mL	10.55 pg/mL	
Std003	1 - A3	19.53 pg/mL	18.91 pg/mL	18.91 pg/mL	
Std004	1 - A4	39.06 pg/mL	37.48 pg/mL	37.48 pg/mL	
Std005	1 - A5	78.13 pg/mL	77.75 pg/mL	77.75 pg/mL	
Std006	1 - A6	156.25 pg/mL	158.90 pg/mL	158.90 pg/mL	
Std007	1 - A7	312.50 pg/mL	311.34 pg/mL	311.34 pg/mL	
Std008	1 - A8	625.00 pg/mL	624.08 pg/mL	624.08 pg/mL	
Std009	1 - A9	1,250.00 pg/mL	1,305.11 pg/mL	1,305.11 pg/mL	
Std010	1 - A10	2,500.00 pg/mL	2,418.58 pg/mL	2,418.58 pg/mL	
V1B	1 - B1	N/A	0.00 pg/mL	0.00 pg/mL	Fitting: Below standard range and out of invertable range
A1B	1 - B2	N/A	0.00 pg/mL	0.00 pg/mL	Fitting: Below standard range and out of invertable range
139 1 B	1 - B3	N/A	0.00 pg/mL	0.00 pg/mL	Fitting: Below standard range and out of invertable range
224 1 B	1 - B4	N/A	0.00 pg/mL	0.00 pg/mL	Fitting: Below standard range and out of invertable range
164 1 B	1 - B5	N/A	0.00 pg/mL	0.00 pg/mL	Fitting: Below standard range and out of invertable range
V1A	1 - C1	N/A	0.00 pg/mL	0.00 pg/mL	Fitting: Below standard range and out of invertable range
A1A	1 - C2	N/A	0.00 pg/mL	0.00 pg/mL	Fitting: Below standard range and out of invertable range

Quantitative Analyte Results for B4 - Mouse IL-6

Name	Plate Position	Nominal CC	Fitted CC	Final CC	Message
139 1 A	1 - C3	N/A	0.00 pg/mL	0.00 pg/mL	Fitting: Below standard range and out of invertable range
224 1 A	1 - C4	N/A	0.00 pg/mL	0.00 pg/mL	Fitting: Below standard range and out of invertable range
164 1 A	1 - C5	N/A	0.00 pg/mL	0.00 pg/mL	Fitting: Out of invertable range
untreated	1 - D1	N/A	0.00 pg/mL	0.00 pg/mL	Fitting: Below standard range and out of invertable range

Results Statistics for C8 - Mouse TNF

Name	Plate Pos.	Clust.	Event #	MFI	SD	CV	Dilution
Std001	1 - A1	Manual	802	259.00	284.84	82.65 %	1.00
Std002	1 - A2	Manual	784	599.00	318.76	49.33 %	1.00
Std003	1 - A3	Manual	1037	790.00	390.29	47.24 %	1.00
Std004	1 - A4	Manual	883	1,355.00	424.02	29.18 %	1.00
Std005	1 - A5	Manual	1000	2,417.00	702.57	26.85 %	1.00
Std006	1 - A6	Manual	860	4,684.00	1,276.15	22.90 %	1.00
Std007	1 - A7	Manual	827	8,794.00	2,652.37	24.46 %	1.00
Std008	1 - A8	Manual	875	16,948.00	4,808.81	22.98 %	1.00
Std009	1 - A9	Manual	847	31,911.00	8,123.17	21.86 %	1.00
Std010	1 - A10	Manual	903	51,494.00	15,497.62	25.15 %	1.00
V1B	1 - B1	Manual	924	271.00	277.80	79.91 %	1.00
A1B	1 - B2	Manual	764	194.00	291.33	92.57 %	1.00
139 1 B	1 - B3	Manual	738	202.00	303.75	93.46 %	1.00
224 1 B	1 - B4	Manual	943	252.00	295.78	85.41 %	1.00
164 1 B	1 - B5	Manual	791	281.00	286.14	79.92 %	1.00
V1A	1 - C1	Manual	1266	262.00	308.57	82.44 %	1.00
A1A	1 - C2	Manual	1270	235.00	267.79	82.72 %	1.00
139 1 A	1 - C3	Manual	1111	254.00	296.52	81.12 %	1.00
224 1 A	1 - C4	Manual	1110	252.00	290.03	81.91 %	1.00
164 1 A	1 - C5	Manual	1024	307.00	259.27	69.35 %	1.00

Results Statistics for C8 - Mouse TNF

Name	Plate Pos.	Clust.	Event #	MFI	SD	CV	Dilution
untreated	1 - D1	Manual	746	229.00	323.02	89.32 %	1.00

Quantitative Analyte Results for C8 - Mouse TNF

Name	Plate Position	Nominal CC	Fitted CC	Final CC	Message
Std001	1 - A1	0.00 pg/mL	0.00 pg/mL	0.00 pg/mL	Fitting: Below standard range and out of invertable range, Recovery: Out of range
Std002	1 - A2	9.77 pg/mL	11.71 pg/mL	11.71 pg/mL	
Std003	1 - A3	19.53 pg/mL	18.17 pg/mL	18.17 pg/mL	
Std004	1 - A4	39.06 pg/mL	37.67 pg/mL	37.67 pg/mL	
Std005	1 - A5	78.13 pg/mL	75.09 pg/mL	75.09 pg/mL	
Std006	1 - A6	156.25 pg/mL	157.07 pg/mL	157.07 pg/mL	
Std007	1 - A7	312.50 pg/mL	312.23 pg/mL	312.23 pg/mL	
Std008	1 - A8	625.00 pg/mL	643.54 pg/mL	643.54 pg/mL	
Std009	1 - A9	1,250.00 pg/mL	1,326.43 pg/mL	1,326.43 pg/mL	
Std010	1 - A10	2,500.00 pg/mL	2,355.74 pg/mL	2,355.74 pg/mL	
V1B	1 - B1	N/A	0.47 pg/mL	0.47 pg/mL	
A1B	1 - B2	N/A	0.00 pg/mL	0.00 pg/mL	Fitting: Below standard range and out of invertable range
139 1 B	1 - B3	N/A	0.00 pg/mL	0.00 pg/mL	Fitting: Below standard range and out of invertable range
224 1 B	1 - B4	N/A	0.00 pg/mL	0.00 pg/mL	Fitting: Below standard range and out of invertable range
164 1 B	1 - B5	N/A	0.97 pg/mL	0.97 pg/mL	
V1A	1 - C1	N/A	0.00 pg/mL	0.00 pg/mL	Fitting: Out of invertable range

Quantitative Analyte Results for C8 - Mouse TNF

Name	Plate Position	Nominal CC	Fitted CC	Final CC	Message
A1A	1 - C2	N/A	0.00 pg/mL	0.00 pg/mL	Fitting: Below standard range and out of invertable range
139 1 A	1 - C3	N/A	0.00 pg/mL	0.00 pg/mL	Fitting: Below standard range and out of invertable range
224 1 A	1 - C4	N/A	0.00 pg/mL	0.00 pg/mL	Fitting: Below standard range and out of invertable range
164 1 A	1 - C5	N/A	1.96 pg/mL	1.96 pg/mL	
untreated	1 - D1	N/A	0.00 pg/mL	0.00 pg/mL	Fitting: Below standard range and out of invertable range

Results Statistics for A8 - Mouse IL-3

Name	Plate Pos.	Clust.	Event #	MFI	SD	CV	Dilution
Std001	1 - A1	Manual	785	1,020.00	339.52	30.24 %	1.00
Std002	1 - A2	Manual	865	2,477.00	670.51	24.21 %	1.00
Std003	1 - A3	Manual	1147	3,731.00	965.17	24.53 %	1.00
Std004	1 - A4	Manual	844	6,210.00	1,660.33	24.33 %	1.00
Std005	1 - A5	Manual	1053	11,554.00	3,057.86	27.08 %	1.00
Std006	1 - A6	Manual	848	20,053.00	5,394.07	23.14 %	1.00
Std007	1 - A7	Manual	797	34,948.00	10,550.92	24.18 %	1.00
Std008	1 - A8	Manual	910	61,635.00	18,666.68	25.97 %	1.00
Std009	1 - A9	Manual	835	101,832.00	30,522.29	23.80 %	1.00
Std010	1 - A10	Manual	744	149,923.00	43,014.49	22.43 %	1.00
V1B	1 - B1	Manual	902	1,005.00	335.99	31.02 %	1.00
A1B	1 - B2	Manual	807	961.00	377.32	37.46 %	1.00
139 1 B	1 - B3	Manual	767	959.00	386.96	39.83 %	1.00
224 1 B	1 - B4	Manual	869	1,011.00	344.70	35.01 %	1.00
164 1 B	1 - B5	Manual	756	1,021.00	311.72	32.31 %	1.00
V1A	1 - C1	Manual	980	940.00	307.82	29.99 %	1.00
A1A	1 - C2	Manual	1150	941.00	302.27	32.45 %	1.00
139 1 A	1 - C3	Manual	1007	962.00	318.02	34.50 %	1.00

Results Statistics for A8 - Mouse IL-3

Name	Plate Pos.	Clust.	Event #	MFI	SD	CV	Dilution
224 1 A	1 - C4	Manual	984	982.00	306.53	29.63 %	1.00
164 1 A	1 - C5	Manual	948	1,032.00	292.44	25.30 %	1.00
untreated	1 - D1	Manual	751	998.00	375.84	33.75 %	1.00

Quantitative Analyte Results for A8 - Mouse IL-3

Name	Plate Position	Nominal CC	Fitted CC	Final CC	Message
Std001	1 - A1	0.00 pg/mL	0.00 pg/mL	0.00 pg/mL	Fitting: Below standard range and out of invertable range, Recovery: Out of range
Std002	1 - A2	9.77 pg/mL	10.42 pg/mL	10.42 pg/mL	
Std003	1 - A3	19.53 pg/mL	19.19 pg/mL	19.19 pg/mL	
Std004	1 - A4	39.06 pg/mL	37.35 pg/mL	37.35 pg/mL	
Std005	1 - A5	78.13 pg/mL	79.63 pg/mL	79.63 pg/mL	
Std006	1 - A6	156.25 pg/mL	154.61 pg/mL	154.61 pg/mL	
Std007	1 - A7	312.50 pg/mL	307.07 pg/mL	307.07 pg/mL	
Std008	1 - A8	625.00 pg/mL	644.65 pg/mL	644.65 pg/mL	
Std009	1 - A9	1,250.00 pg/mL	1,311.37 pg/mL	1,311.37 pg/mL	
Std010	1 - A10	2,500.00 pg/mL	2,377.53 pg/mL	2,377.53 pg/mL	
V1B	1 - B1	N/A	0.00 pg/mL	0.00 pg/mL	Fitting: Below standard range and out of invertable range
A1B	1 - B2	N/A	0.00 pg/mL	0.00 pg/mL	Fitting: Below standard range and out of invertable range
139 1 B	1 - B3	N/A	0.00 pg/mL	0.00 pg/mL	Fitting: Below standard range and out of invertable range
224 1 B	1 - B4	N/A	0.00 pg/mL	0.00 pg/mL	Fitting: Below standard range and out of invertable range

Quantitative Analyte Results for A8 - Mouse IL-3

Name	Plate Position	Nominal CC	Fitted CC	Final CC	Message
164 1 B	1 - B5	N/A	0.00 pg/mL	0.00 pg/mL	Fitting: Out of invertable range
V1A	1 - C1	N/A	0.00 pg/mL	0.00 pg/mL	Fitting: Below standard range and out of invertable range
A1A	1 - C2	N/A	0.00 pg/mL	0.00 pg/mL	Fitting: Below standard range and out of invertable range
139 1 A	1 - C3	N/A	0.00 pg/mL	0.00 pg/mL	Fitting: Below standard range and out of invertable range
224 1 A	1 - C4	N/A	0.00 pg/mL	0.00 pg/mL	Fitting: Below standard range and out of invertable range
164 1 A	1 - C5	N/A	0.12 pg/mL	0.12 pg/mL	
untreated	1 - D1	N/A	0.00 pg/mL	0.00 pg/mL	Fitting: Below standard range and out of invertable range

Sample File Assignment

Sample Name	File Name
Std001	A10 Assay diluent.fcs
Std002	A01 ST 1_256.fcs
Std003	A02 ST 1_128.fcs
Std004	A03 ST 1_64.fcs
Std005	A04 ST 1_32.fcs
Std006	A05 ST 1_16.fcs
Std007	A06 ST 1_8.fcs
Std008	A07 ST 1_4.fcs
Std009	A08 ST 1_2.fcs
Std010	A09 top std.fcs
V1B	B04 V1B 20161014.fcs
A1B	C04 A1B 20161014.fcs
139 1 B	D04 139_1B 20161014.fcs
224 1 B	E04 224_1B 20161014.fcs
164 1 B	F04 164_1B 20161014.fcs
V1A	B04 V1A 20161013.fcs
A1A	C04 A1A 20161013.fcs
139 1 A	D04 139_1 20161013.fcs
224 1 A	E04 224_1 20161013.fcs
164 1 A	F04 164_1 20161013.fcs
untreated	A12 untreated ctrl.fcs
