# Supplementary material for: In silico-guided sequence modifications of K-ras epitopes improve immunological outcome against G12V and G13D mutant KRAS antigens
Source: PeerJ. 2018 Jul 20;6:e5056. doi: 10.7717/peerj.5056 (PMC6055689; doi:10.7717/peerj.5056)
Supplement: Supplemental Information 12 — A file generated by BD Bioscience reporting mouse IL-2, IL-4, IL-5, IL-6, IL-10, IL12p70, IL-21, TNF-alpha and IFN-gamma components on PBMC samples treated with synthetic peptide mimotopes 68-V, 164-D and 224-D. PBMC sampled treated with synthetic peptide mimotopes wild-type KRAS, G12V and G13D were included as controls. Untreated PBMC samples and standard curve of all cytokine components were also included. All data replicates are reported in number of events, median fluorescence intensity (MFI), nominal concentration (pg/mL), fitted concentration (%), and percentage of recovery (%). [file peerj-06-5056-s012.pdf]

**Plex Name:**	New Plex
**Created by:**	Administrator
**Creation Date:**	5/31/2017
**Intrument:**	BD Accuri C6 Plus

Instrument

Instrument Name:	BD Accuri C6 Plus
Scatter Parameter:	FSC-A
Number of Scatter Peaks:	1
Clustering Parameter(s):	FL4-A, FL3-A
Reporter Parameter(s):	PE-A

Debris Filter is active!

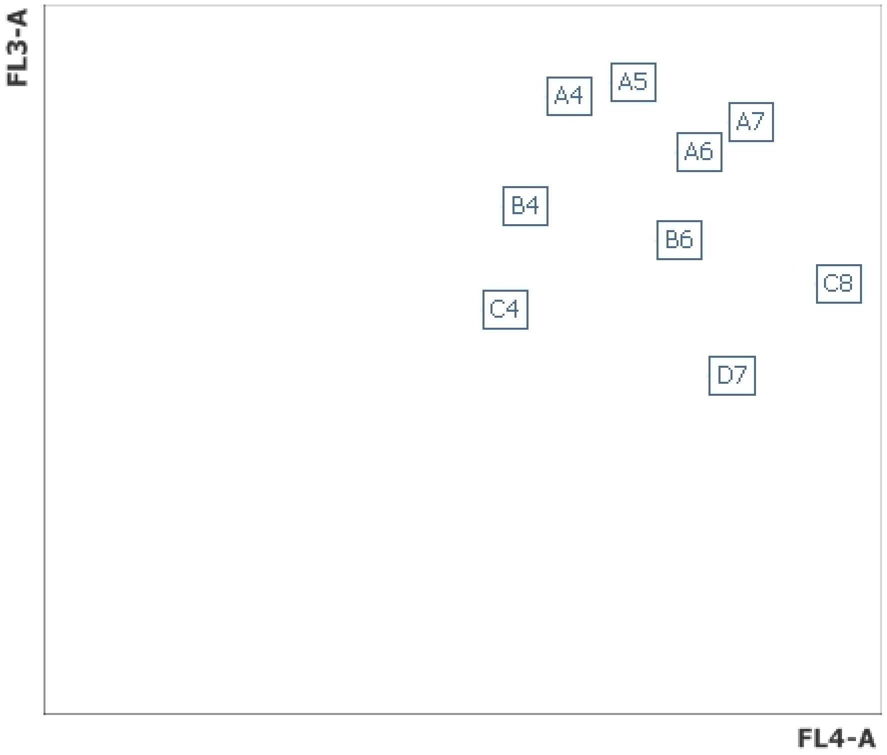

Layout 1

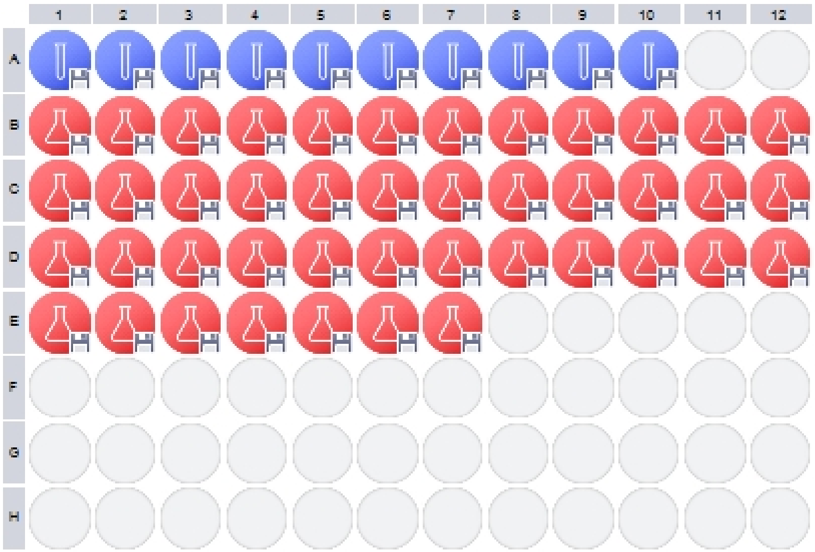

Plex Components

		Analyte		
Name	Lot Number	Name	Model	2nd Reporter
C4		Mouse IL-10	Quantitative	No
D7		Mouse IL-12p70	Quantitative	No
B6		Mouse IL-21	Quantitative	No
A5		Mouse IL-2	Quantitative	No
A7		Mouse IL-4	Quantitative	No
A6		Mouse IL-5	Quantitative	No
B4		Mouse IL-6	Quantitative	No
A4		Mouse IFN-γ	Quantitative	No
C8		Mouse TNF	Quantitative	No

Standard Samples of Quantitative Analysis

Reporter Parameter 1	
Sample Name	Concentration
Std001	0.00 pg/mL
Std002	10.00 pg/mL

Standard Samples of Quantitative Analysis

Reporter Parameter 1	
Sample Name	Concentration
Std003	20.00 pg/mL
Std004	40.00 pg/mL
Std005	80.00 pg/mL
Std006	156.00 pg/mL
Std007	312.50 pg/mL
Std008	625.00 pg/mL
Std009	1,250.00 pg/mL
Std010	2,500.00 pg/mL

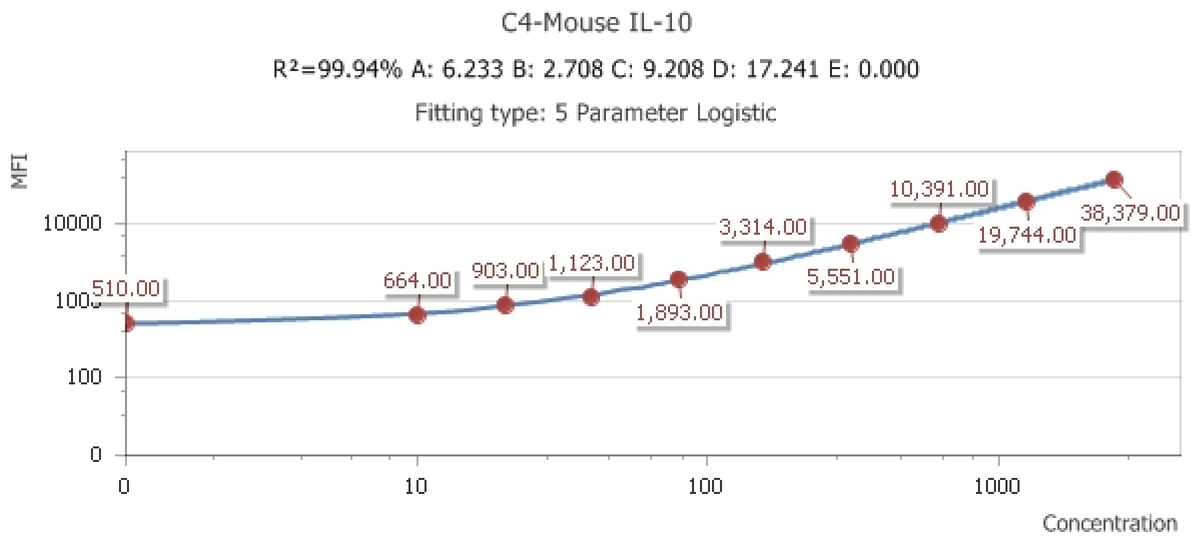

Name	Event #	MFI	SD	CV% (MFI)	Nominal CC pg/mL	Fitted CC pg/mL	Recovery %
Std001	302	510.00	496.30	72.59 %	0.00	0.41	0.00 %
Std002	305	664.00	542.26	62.58 %	10.00	9.46	94.57 %
Std003	321	903.00	568.95	56.06 %	20.00	22.39	111.96 %
Std004	710	1,123.00	255.19	21.43 %	40.00	34.84	87.10 %
Std005	313	1,893.00	432.18	23.57 %	80.00	80.49	100.61 %
Std006	316	3,314.00	686.63	20.41 %	156.00	167.82	107.58 %
Std007	302	5,551.00	883.44	14.60 %	312.50	307.80	98.50 %
Std008	298	10,391.00	1,984.27	17.47 %	625.00	615.93	98.55 %
Std009	281	19,744.00	3,660.17	15.77 %	1,250.00	1,230.58	98.45 %
Std010	295	38,379.00	7,883.73	17.81 %	2,500.00	2,534.96	101.40 %

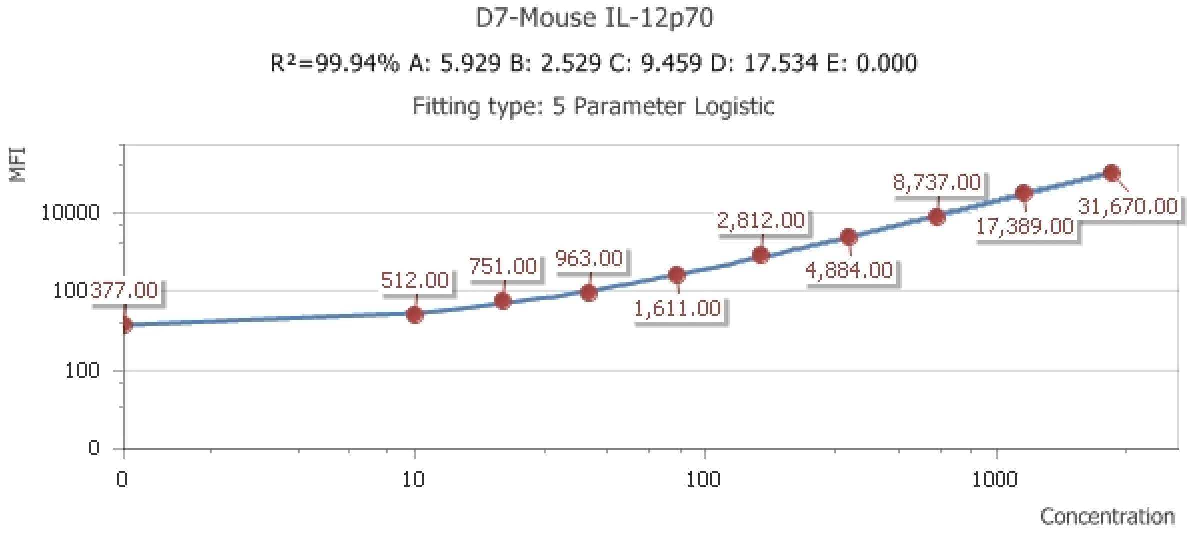

Name	Event #	MFI	SD	CV% (MFI)	Nominal CC pg/mL	Fitted CC pg/mL	Recovery %
Std001	250	377.00	467.39	80.22 %	0.00	0.46	0.00 %
Std002	270	512.00	577.29	75.20 %	10.00	8.79	87.89 %
Std003	294	751.00	527.62	59.01 %	20.00	23.04	115.22 %
Std004	115	963.00	252.04	24.78 %	40.00	36.44	91.10 %
Std005	273	1,611.00	393.26	21.88 %	80.00	79.60	99.51 %
Std006	264	2,812.00	497.41	17.94 %	156.00	163.36	104.72 %
Std007	267	4,884.00	843.60	13.51 %	312.50	312.67	100.05 %
Std008	288	8,737.00	1,159.39	11.78 %	625.00	599.71	95.95 %
Std009	286	17,389.00	3,275.06	14.15 %	1,250.00	1,279.11	102.33 %
Std010	284	31,670.00	5,326.24	13.63 %	2,500.00	2,494.00	99.76 %

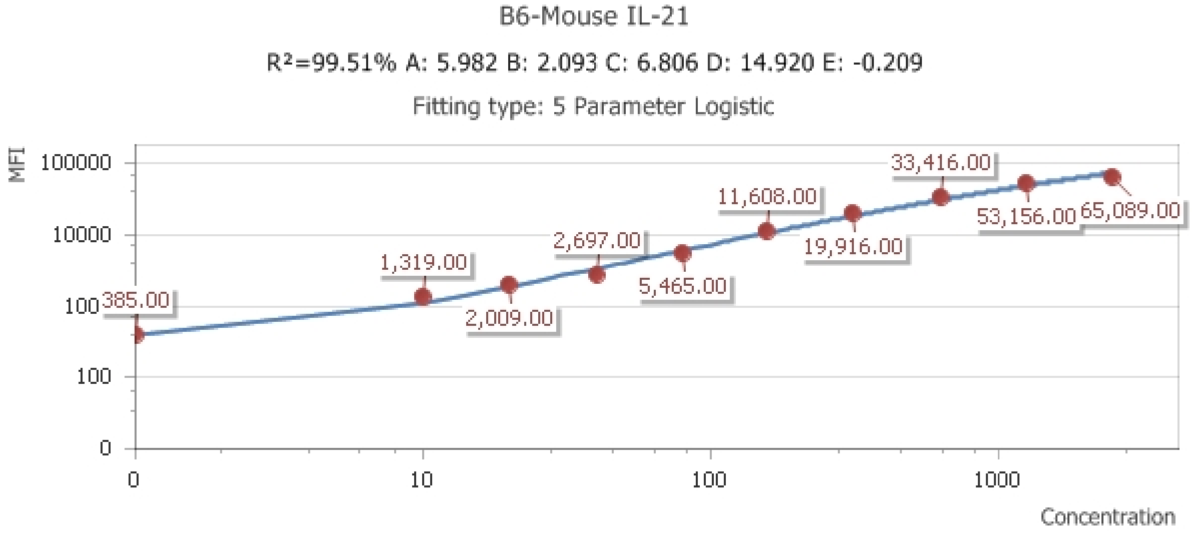

Name	Event #	MFI	SD	CV% (MFI)	Nominal CC pg/mL	Fitted CC pg/mL	Recovery %
Std001	257	385.00	433.66	80.82 %	0.00	0.00	0.00 %
Std002	259	1,319.00	565.61	37.15 %	10.00	12.31	123.05 %
Std003	255	2,009.00	624.17	27.46 %	20.00	21.35	106.73 %
Std004	284	2,697.00	514.28	18.82 %	40.00	30.59	76.47 %
Std005	278	5,465.00	1,175.33	21.70 %	80.00	70.20	87.75 %
Std006	279	11,608.00	2,268.38	19.23 %	156.00	172.27	110.43 %
Std007	289	19,916.00	4,451.14	19.84 %	312.50	341.58	109.31 %
Std008	269	33,416.00	6,753.24	19.99 %	625.00	696.82	111.49 %
Std009	310	53,156.00	8,737.89	16.15 %	1,250.00	1,414.41	113.15 %
Std010	265	65,089.00	10,310.74	16.28 %	2,500.00	1,978.12	79.12 %

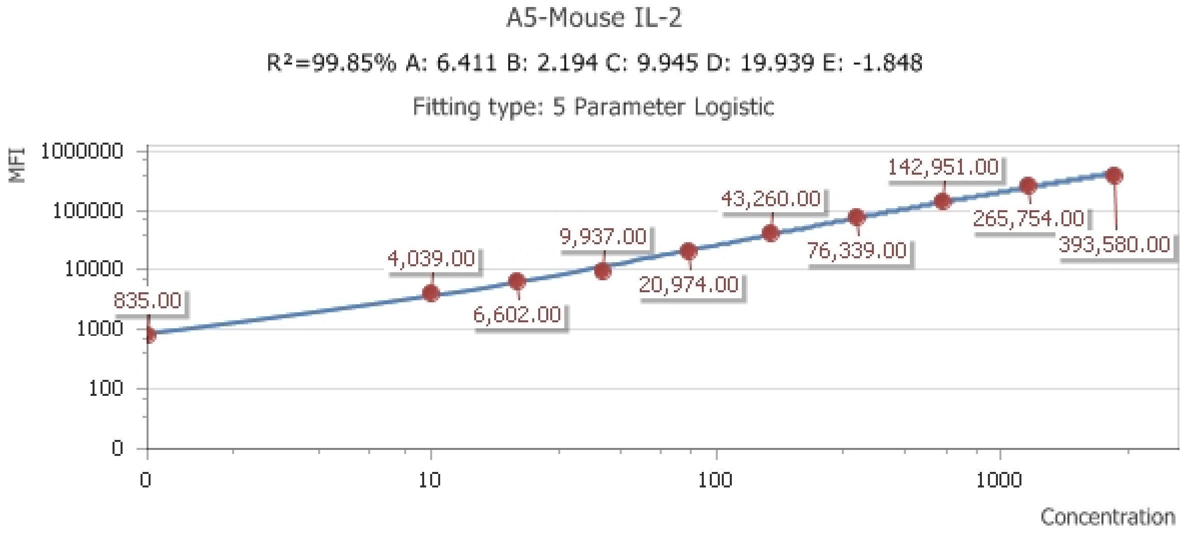

Name	Event #	MFI	SD	CV% (MFI)	Nominal CC pg/mL	Fitted CC pg/mL	Recovery %
Std001	274	835.00	524.10	47.41 %	0.00	0.00	0.00 %
Std002	237	4,039.00	795.04	16.81 %	10.00	11.17	111.69 %
Std003	241	6,602.00	1,332.12	17.77 %	20.00	20.69	103.46 %
Std004	374	9,937.00	1,805.99	15.94 %	40.00	33.34	83.35 %
Std005	257	20,974.00	4,224.67	18.03 %	80.00	76.56	95.70 %
Std006	251	43,260.00	7,249.91	16.80 %	156.00	168.88	108.26 %
Std007	280	76,339.00	16,047.85	17.93 %	312.50	316.65	101.33 %
Std008	280	142,951.00	27,722.77	17.67 %	625.00	648.06	103.69 %
Std009	268	265,754.00	44,102.35	15.08 %	1,250.00	1,363.20	109.06 %
Std010	291	393,580.00	57,123.84	12.43 %	2,500.00	2,238.24	89.53 %

A7-Mouse IL-4

R^2=99.82% A: 6.570 B: 2.333 C: 7.448 D: 17.955 E: -0.153

Fitting type: 5 Parameter Logistic

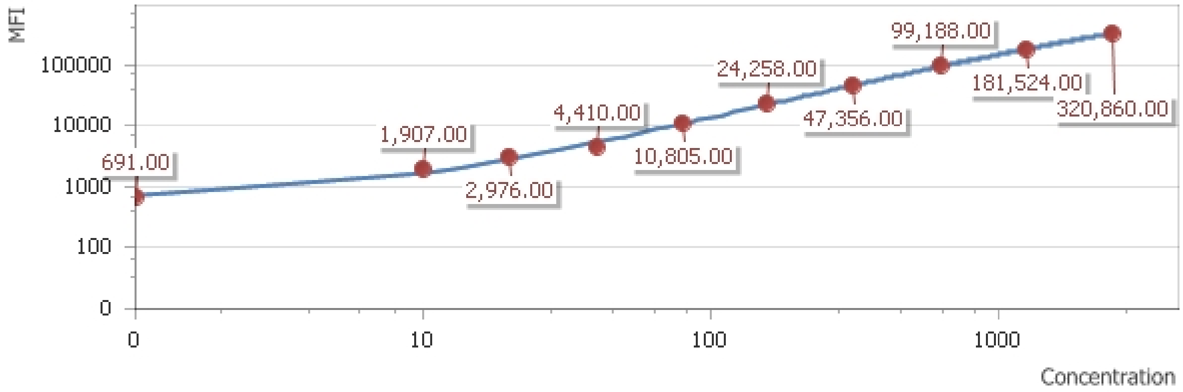

Name	Event #	MFI	SD	CV% (MFI)	Nominal CC pg/mL	Fitted CC pg/mL	Recovery %
Std001	238	691.00	505.01	60.67 %	0.00	0.00	0.00 %
Std002	264	1,907.00	550.79	27.70 %	10.00	11.89	118.93 %
Std003	242	2,976.00	880.48	26.49 %	20.00	20.92	104.58 %
Std004	261	4,410.00	962.58	20.61 %	40.00	32.31	80.78 %
Std005	276	10,805.00	2,680.17	24.43 %	80.00	78.64	98.30 %
Std006	263	24,258.00	5,094.95	20.44 %	156.00	168.02	107.71 %
Std007	283	47,356.00	12,341.16	26.86 %	312.50	316.23	101.19 %
Std008	256	99,188.00	23,978.09	23.75 %	625.00	655.85	104.94 %
Std009	231	181,524.00	40,974.62	20.29 %	1,250.00	1,241.86	99.35 %
Std010	283	320,860.00	59,687.99	17.34 %	2,500.00	2,386.61	95.46 %

A6-Mouse IL-5

R^2=99.86% A: 6.459 B: 2.273 C: 7.487 D: 18.002 E: -0.150

Fitting type: 5 Parameter Logistic

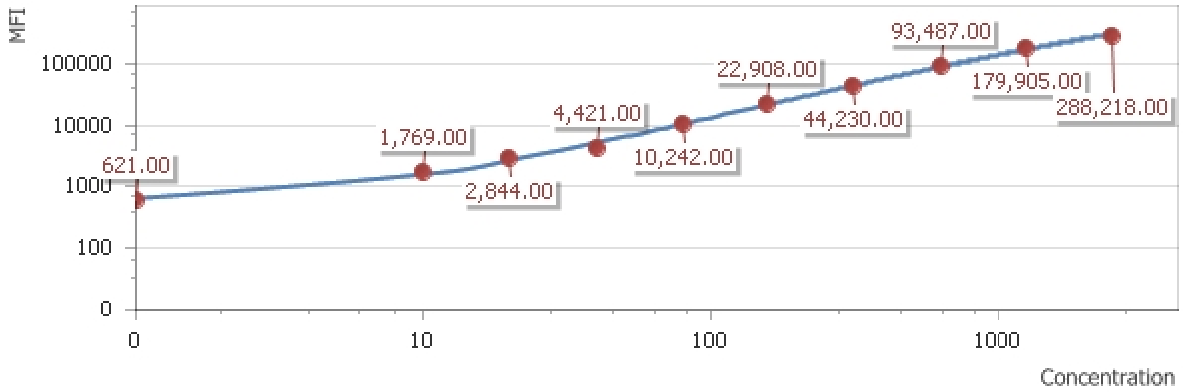

Name	Event #	MFI	SD	CV% (MFI)	Nominal CC pg/mL	Fitted CC pg/mL	Recovery %
Std001	281	621.00	472.58	63.82 %	0.00	0.00	0.00 %
Std002	298	1,769.00	507.42	26.39 %	10.00	11.55	115.51 %
Std003	321	2,844.00	717.21	25.29 %	20.00	20.89	104.45 %
Std004	486	4,421.00	1,018.18	22.19 %	40.00	33.77	84.42 %
Std005	299	10,242.00	2,418.12	22.00 %	80.00	77.47	96.84 %
Std006	280	22,908.00	5,302.33	21.39 %	156.00	165.75	106.25 %
Std007	306	44,230.00	11,191.22	22.59 %	312.50	310.60	99.39 %
Std008	416	93,487.00	23,535.16	23.55 %	625.00	655.61	104.90 %
Std009	300	179,905.00	35,816.84	18.00 %	1,250.00	1,324.10	105.93 %
Std010	293	288,218.00	53,491.10	15.56 %	2,500.00	2,289.69	91.59 %

B4-Mouse IL-6

R^2=99.86% A: 6.271 B: 2.474 C: 10.727 D: 19.476 E: -1.678

Fitting type: 5 Parameter Logistic

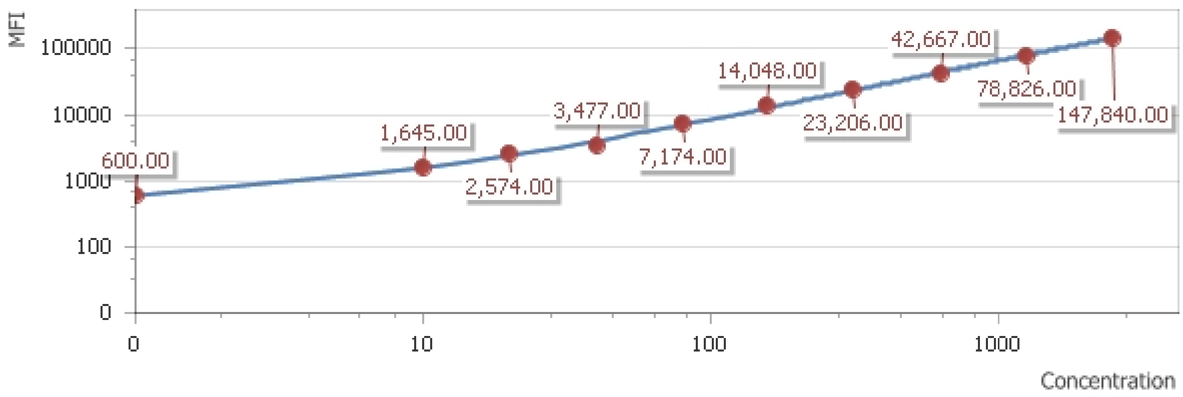

Name	Event #	MFI	SD	CV% (MFI)	Nominal CC pg/mL	Fitted CC pg/mL	Recovery %
Std001	277	600.00	447.00	62.25 %	0.00	0.00	0.00 %
Std002	288	1,645.00	543.93	29.82 %	10.00	10.55	105.46 %
Std003	264	2,574.00	621.21	23.84 %	20.00	21.48	107.39 %
Std004	250	3,477.00	701.83	19.51 %	40.00	32.66	81.66 %
Std005	295	7,174.00	1,404.02	22.95 %	80.00	81.04	101.30 %
Std006	270	14,048.00	2,855.12	18.29 %	156.00	176.23	112.97 %
Std007	259	23,206.00	4,725.79	18.93 %	312.50	309.34	98.99 %
Std008	259	42,667.00	9,503.47	18.94 %	625.00	609.80	97.57 %
Std009	247	78,826.00	16,086.95	19.27 %	1,250.00	1,219.54	97.56 %
Std010	270	147,840.00	28,752.62	17.97 %	2,500.00	2,538.58	101.54 %

A4-Mouse IFN-γ

R^2=99.88% A: 6.280 B: 2.958 C: 10.397 D: 18.232 E: -2.943

Fitting type: 5 Parameter Logistic

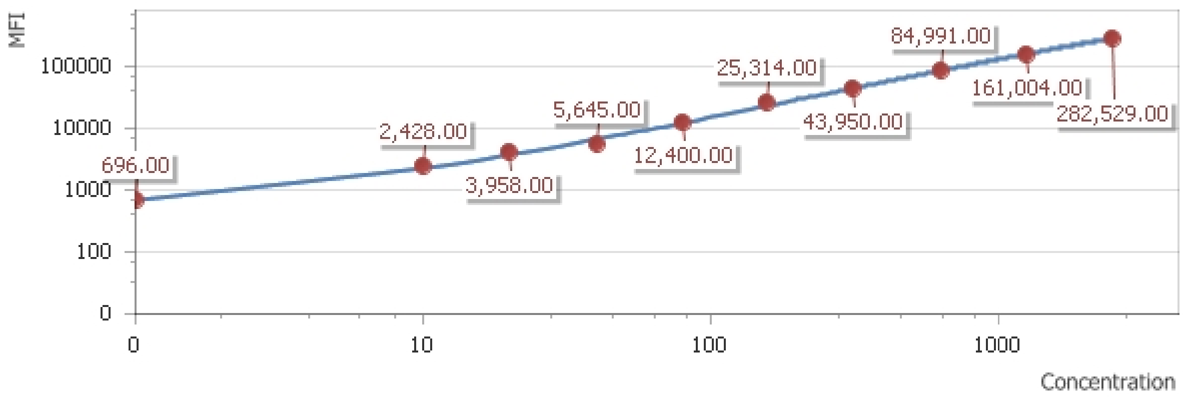

Name	Event #	MFI	SD	CV% (MFI)	Nominal CC pg/mL	Fitted CC pg/mL	Recovery %
Std001	293	696.00	484.44	56.71 %	0.00	0.00	0.00 %
Std002	287	2,428.00	589.33	21.79 %	10.00	10.84	108.45 %
Std003	313	3,958.00	755.01	17.98 %	20.00	21.33	106.63 %
Std004	408	5,645.00	1,214.43	19.43 %	40.00	33.04	82.61 %
Std005	268	12,400.00	2,366.23	17.59 %	80.00	80.32	100.40 %
Std006	290	25,314.00	4,210.21	15.55 %	156.00	171.72	110.07 %
Std007	306	43,950.00	8,085.17	17.15 %	312.50	307.02	98.25 %
Std008	326	84,991.00	17,389.60	17.57 %	625.00	621.69	99.47 %
Std009	294	161,004.00	31,183.34	19.37 %	1,250.00	1,266.32	101.31 %
Std010	300	282,529.00	49,223.43	16.00 %	2,500.00	2,458.61	98.34 %

C8-Mouse TNF

R^2=99.92% A: 5.985 B: 4.935 C: 10.227 D: 13.737 E: -3.681

Fitting type: 5 Parameter Logistic

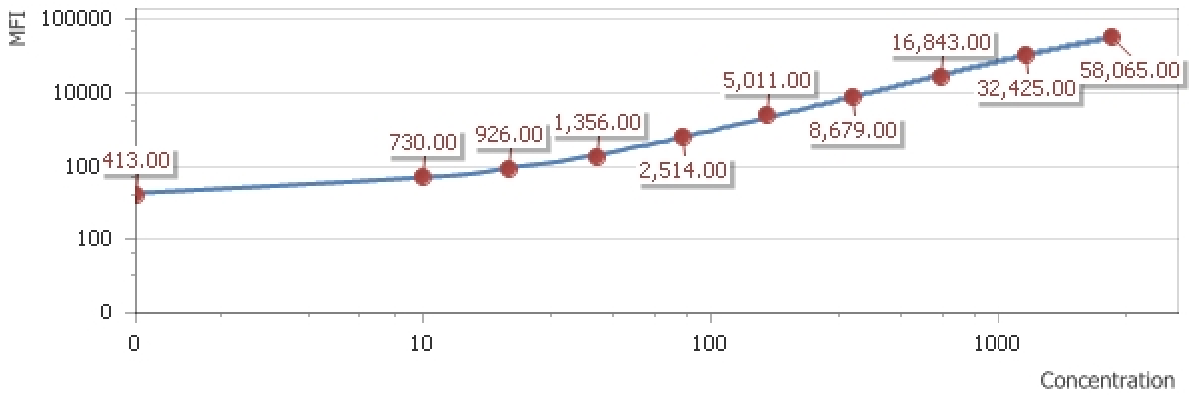

Name	Event #	MFI	SD	CV% (MFI)	Nominal CC pg/mL	Fitted CC pg/mL	Recovery %
Std001	278	413.00	472.21	81.98 %	0.00	0.00	0.00 %
Std002	295	730.00	557.46	61.22 %	10.00	11.51	115.10 %
Std003	328	926.00	557.27	53.56 %	20.00	19.12	95.60 %
Std004	365	1,356.00	293.18	20.01 %	40.00	35.91	89.79 %
Std005	295	2,514.00	550.04	20.26 %	80.00	80.22	100.27 %
Std006	296	5,011.00	888.26	17.23 %	156.00	172.07	110.30 %
Std007	296	8,679.00	1,757.62	19.05 %	312.50	304.37	97.40 %
Std008	276	16,843.00	3,277.66	17.31 %	625.00	605.89	96.94 %
Std009	290	32,425.00	6,703.76	21.08 %	1,250.00	1,244.43	99.55 %
Std010	306	58,065.00	11,822.07	18.46 %	2,500.00	2,540.28	101.61 %

Results Statistics for C4 - Mouse IL-10

Name	Plate Pos.	Clust.	Event #	MFI	SD	CV	Dilution
Std001	1 - A1	Auto	302	510.00	496.30	72.59 %	1.00
Std002	1 - A2	Auto	305	664.00	542.26	62.58 %	1.00
Std003	1 - A3	Auto	321	903.00	568.95	56.06 %	1.00
Std004	1 - A4	Auto	710	1,123.00	255.19	21.43 %	1.00
Std005	1 - A5	Auto	313	1,893.00	432.18	23.57 %	1.00
Std006	1 - A6	Auto	316	3,314.00	686.63	20.41 %	1.00
Std007	1 - A7	Auto	302	5,551.00	883.44	14.60 %	1.00
Std008	1 - A8	Auto	298	10,391.00	1,984.27	17.47 %	1.00
Std009	1 - A9	Auto	281	19,744.00	3,660.17	15.77 %	1.00
Std010	1 - A10	Auto	295	38,379.00	7,883.73	17.81 %	1.00
Test001	1 - B1	Auto	284	521.00	487.40	75.65 %	1.00
Test002	1 - B2	Auto	319	472.00	540.41	77.40 %	1.00
Test003	1 - B3	Auto	306	514.00	469.06	68.72 %	1.00
Test004	1 - B4	Auto	279	486.00	467.02	71.92 %	1.00
Test005	1 - B5	Auto	326	546.00	290.77	47.83 %	1.00
Test006	1 - B6	Auto	292	542.00	287.07	47.64 %	1.00
Test007	1 - B7	Auto	299	466.00	345.45	62.82 %	1.00
Test008	1 - B8	Auto	357	507.00	407.72	62.77 %	1.00
Test009	1 - B9	Auto	371	516.00	301.71	52.28 %	1.00
Test010	1 - B10	Auto	374	569.00	432.55	60.57 %	1.00
Test011	1 - B11	Auto	344	511.00	482.77	72.04 %	1.00
Test012	1 - B12	Auto	383	528.00	463.31	63.93 %	1.00
Test013	1 - C1	Auto	343	533.00	349.89	55.74 %	1.00
Test014	1 - C2	Auto	333	472.00	354.34	61.97 %	1.00
Test015	1 - C3	Auto	344	586.00	545.23	69.55 %	1.00
Test016	1 - C4	Auto	318	514.00	475.17	71.67 %	1.00
Test017	1 - C5	Auto	355	447.00	543.37	77.64 %	1.00
Test018	1 - C6	Auto	374	533.00	636.59	80.86 %	1.00
Test019	1 - C7	Auto	311	399.00	432.18	78.04 %	1.00
Test020	1 - C8	Auto	364	560.00	423.65	62.25 %	1.00
Test021	1 - C9	Auto	376	514.00	481.47	70.42 %	1.00
Test022	1 - C10	Auto	261	412.00	366.20	69.29 %	1.00

Results Statistics for C4 - Mouse IL-10

Name	Plate Pos.	Clust.	Event #	MFI	SD	CV	Dilution
Test023	1 - C11	Auto	291	446.00	223.13	40.99 %	1.00
Test024	1 - C12	Auto	282	425.00	195.15	39.42 %	1.00
Test025	1 - D1	Auto	274	435.00	163.09	32.82 %	1.00
Test026	1 - D2	Auto	275	379.00	329.88	64.17 %	1.00
Test027	1 - D3	Auto	263	366.00	252.04	59.30 %	1.00
Test028	1 - D4	Manual	311	394.00	366.20	71.27 %	1.00
Test029	1 - D5	Manual	270	422.00	326.73	62.97 %	1.00
Test030	1 - D6	Auto	253	367.00	329.51	65.86 %	1.00
Test031	1 - D7	Auto	295	424.00	230.54	46.95 %	1.00
Test032	1 - D8	Auto	282	430.00	270.57	59.28 %	1.00
Test033	1 - D9	Auto	271	419.00	233.51	51.30 %	1.00
Test034	1 - D10	Manual	279	389.00	255.01	59.92 %	1.00
Test035	1 - D11	Auto	308	433.00	204.23	41.41 %	1.00
Test036	1 - D12	Auto	253	383.00	242.03	57.62 %	1.00
Test037	1 - E1	Auto	296	418.00	247.41	51.67 %	1.00
Test038	1 - E2	Auto	262	436.00	262.79	55.93 %	1.00
Test039	1 - E3	Auto	264	408.00	249.08	56.27 %	1.00
Test040	1 - E4	Auto	285	452.00	167.16	31.68 %	1.00
Test041	1 - E5	Auto	299	439.00	312.83	57.27 %	1.00
Test042	1 - E6	Auto	267	405.00	264.64	56.09 %	1.00
Test044	1 - E7	Auto	299	424.00	290.59	56.51 %	1.00

Quantitative Analyte Results for C4 - Mouse IL-10

Name	Plate Position	Nominal CC	Fitted CC	Final CC	Message
Std001	1 - A1	0.00	0.41	0.41	Fitting: Below standard range
Std002	1 - A2	10.00	9.46	9.46	
Std003	1 - A3	20.00	22.39	22.39	
Std004	1 - A4	40.00	34.84	34.84	
Std005	1 - A5	80.00	80.49	80.49	
Std006	1 - A6	156.00	167.82	167.82	
Std007	1 - A7	312.50	307.80	307.80	
Std008	1 - A8	625.00	615.93	615.93	
Std009	1 - A9	1,250.00	1,230.58	1,230.58	
Std010	1 - A10	2,500.00	2,534.96	2,534.96	
Test001	1 - B1	N/A	1.56	1.56	
Test002	1 - B2	N/A	0.00	0.00	Fitting: Below standard range and out of invertable range
Test003	1 - B3	N/A	0.96	0.96	
Test004	1 - B4	N/A	0.00	0.00	Fitting: Below standard range and out of invertable range
Test005	1 - B5	N/A	3.15	3.15	
Test006	1 - B6	N/A	2.92	2.92	

Quantitative Analyte Results for C4 - Mouse IL-10

Name	Plate Position	Nominal CC	Fitted CC	Final CC	Message
Test007	1 - B7	N/A	0.00	0.00	Fitting: Below standard range and out of invertable range
Test008	1 - B8	N/A	0.00	0.00	Fitting: Below standard range and out of invertable range
Test009	1 - B9	N/A	1.16	1.16	
Test010	1 - B10	N/A	4.43	4.43	
Test011	1 - B11	N/A	0.60	0.60	
Test012	1 - B12	N/A	2.05	2.05	
Test013	1 - C1	N/A	2.37	2.37	
Test014	1 - C2	N/A	0.00	0.00	Fitting: Below standard range and out of invertable range
Test015	1 - C3	N/A	5.34	5.34	
Test016	1 - C4	N/A	0.96	0.96	
Test017	1 - C5	N/A	0.00	0.00	Fitting: Below standard range and out of invertable range
Test018	1 - C6	N/A	2.37	2.37	
Test019	1 - C7	N/A	0.00	0.00	Fitting: Below standard range and out of invertable range
Test020	1 - C8	N/A	3.94	3.94	
Test021	1 - C9	N/A	0.96	0.96	
Test022	1 - C10	N/A	0.00	0.00	Fitting: Below standard range and out of invertable range
Test023	1 - C11	N/A	0.00	0.00	Fitting: Below standard range and out of invertable range
Test024	1 - C12	N/A	0.00	0.00	Fitting: Below standard range and out of invertable range
Test025	1 - D1	N/A	0.00	0.00	Fitting: Below standard range and out of invertable range
Test026	1 - D2	N/A	0.00	0.00	Fitting: Below standard range and out of invertable range
Test027	1 - D3	N/A	0.00	0.00	Fitting: Below standard range and out of invertable range
Test028	1 - D4	N/A	0.00	0.00	Fitting: Below standard range and out of invertable range
Test029	1 - D5	N/A	0.00	0.00	Fitting: Below standard range and out of invertable range
Test030	1 - D6	N/A	0.00	0.00	Fitting: Below standard range and out of invertable range
Test031	1 - D7	N/A	0.00	0.00	Fitting: Below standard range and out of invertable range
Test032	1 - D8	N/A	0.00	0.00	Fitting: Below standard range and out of invertable range
Test033	1 - D9	N/A	0.00	0.00	Fitting: Below standard range and out of invertable range
Test034	1 - D10	N/A	0.00	0.00	Fitting: Below standard range and out of invertable range
Test035	1 - D11	N/A	0.00	0.00	Fitting: Below standard range and out of invertable range
Test036	1 - D12	N/A	0.00	0.00	Fitting: Below standard range

Quantitative Analyte Results for C4 - Mouse IL-10

Name	Plate Position	Nominal CC	Fitted CC	Final CC	Message
Test036	1 - D12	N/A	0.00	0.00	and out of invertable range
Test037	1 - E1	N/A	0.00	0.00	Fitting: Below standard range and out of invertable range
Test038	1 - E2	N/A	0.00	0.00	Fitting: Below standard range and out of invertable range
Test039	1 - E3	N/A	0.00	0.00	Fitting: Below standard range and out of invertable range
Test040	1 - E4	N/A	0.00	0.00	Fitting: Below standard range and out of invertable range
Test041	1 - E5	N/A	0.00	0.00	Fitting: Below standard range and out of invertable range
Test042	1 - E6	N/A	0.00	0.00	Fitting: Below standard range and out of invertable range
Test044	1 - E7	N/A	0.00	0.00	Fitting: Below standard range and out of invertable range

Results Statistics for D7 - Mouse IL-12p70

Name	Plate Pos.	Clust.	Event #	MFI	SD	CV	Dilution
Std001	1 - A1	Auto	250	377.00	467.39	80.22 %	1.00
Std002	1 - A2	Auto	270	512.00	577.29	75.20 %	1.00
Std003	1 - A3	Auto	294	751.00	527.62	59.01 %	1.00
Std004	1 - A4	Auto	115	963.00	252.04	24.78 %	1.00
Std005	1 - A5	Auto	273	1,611.00	393.26	21.88 %	1.00
Std006	1 - A6	Auto	264	2,812.00	497.41	17.94 %	1.00
Std007	1 - A7	Auto	267	4,884.00	843.60	13.51 %	1.00
Std008	1 - A8	Auto	288	8,737.00	1,159.39	11.78 %	1.00
Std009	1 - A9	Auto	286	17,389.00	3,275.06	14.15 %	1.00
Std010	1 - A10	Auto	284	31,670.00	5,326.24	13.63 %	1.00
Test001	1 - B1	Auto	284	369.00	490.56	88.13 %	1.00
Test002	1 - B2	Auto	272	395.00	489.26	85.32 %	1.00
Test003	1 - B3	Auto	274	358.00	470.35	83.54 %	1.00
Test004	1 - B4	Auto	264	312.00	473.32	92.09 %	1.00
Test005	1 - B5	Auto	279	421.00	310.60	63.81 %	1.00
Test006	1 - B6	Auto	286	403.00	266.68	57.45 %	1.00
Test007	1 - B7	Auto	266	438.00	395.48	69.15 %	1.00
Test008	1 - B8	Auto	295	412.00	413.65	71.71 %	1.00
Test009	1 - B9	Auto	264	432.00	294.11	59.19 %	1.00
Test010	1 - B10	Auto	270	435.00	425.88	72.85 %	1.00
Test011	1 - B11	Auto	264	420.00	419.95	75.18 %	1.00
Test012	1 - B12	Auto	232	420.00	396.22	72.88 %	1.00
Test013	1 - C1	Auto	241	383.00	348.04	67.79 %	1.00
Test014	1 - C2	Auto	274	440.00	376.02	67.80 %	1.00
Test015	1 - C3	Auto	268	389.00	457.38	82.44 %	1.00
Test016	1 - C4	Auto	281	414.00	459.61	78.57 %	1.00

Results Statistics for D7 - Mouse IL-12p70

Name	Plate Pos.	Clust.	Event #	MFI	SD	CV	Dilution
Test017	1 - C5	Auto	233	320.00	444.04	87.76 %	1.00
Test018	1 - C6	Auto	225	301.00	503.71	94.08 %	1.00
Test019	1 - C7	Auto	253	381.00	432.92	83.07 %	1.00
Test020	1 - C8	Auto	262	363.00	394.56	72.30 %	1.00
Test021	1 - C9	Auto	220	291.00	351.19	84.49 %	1.00
Test022	1 - C10	Auto	223	234.00	329.14	89.02 %	1.00
Test023	1 - C11	Auto	210	357.00	187.73	48.70 %	1.00
Test024	1 - C12	Auto	249	314.00	175.69	48.93 %	1.00
Test025	1 - D1	Auto	213	404.00	163.09	37.01 %	1.00
Test026	1 - D2	Auto	235	277.00	269.09	76.99 %	1.00
Test027	1 - D3	Auto	217	338.00	256.12	68.25 %	1.00
Test028	1 - D4	Manual	250	274.00	342.67	80.34 %	1.00
Test029	1 - D5	Manual	252	312.00	307.64	75.70 %	1.00
Test030	1 - D6	Auto	248	270.00	309.31	79.35 %	1.00
Test031	1 - D7	Auto	241	363.00	226.84	53.48 %	1.00
Test032	1 - D8	Auto	234	349.00	268.35	67.14 %	1.00
Test033	1 - D9	Auto	283	329.00	290.59	68.28 %	1.00
Test034	1 - D10	Manual	281	343.00	311.72	69.59 %	1.00
Test035	1 - D11	Auto	175	345.00	219.42	52.35 %	1.00
Test036	1 - D12	Auto	223	297.00	225.36	71.06 %	1.00
Test037	1 - E1	Auto	195	295.00	215.72	62.60 %	1.00
Test038	1 - E2	Auto	228	303.00	280.95	69.99 %	1.00
Test039	1 - E3	Auto	236	305.00	260.20	65.98 %	1.00
Test040	1 - E4	Auto	217	359.00	156.04	36.81 %	1.00
Test041	1 - E5	Auto	244	326.00	270.20	67.58 %	1.00
Test042	1 - E6	Auto	245	319.00	278.73	67.43 %	1.00
Test044	1 - E7	Auto	237	309.00	253.15	65.42 %	1.00

Quantitative Analyte Results for D7 - Mouse IL-12p70

Name	Plate Position	Nominal CC	Fitted CC	Final CC	Message
Std001	1 - A1	0.00	0.46	0.46	Fitting: Below standard range
Std002	1 - A2	10.00	8.79	8.79	
Std003	1 - A3	20.00	23.04	23.04	
Std004	1 - A4	40.00	36.44	36.44	
Std005	1 - A5	80.00	79.60	79.60	
Std006	1 - A6	156.00	163.36	163.36	
Std007	1 - A7	312.50	312.67	312.67	
Std008	1 - A8	625.00	599.71	599.71	
Std009	1 - A9	1,250.00	1,279.11	1,279.11	
Std010	1 - A10	2,500.00	2,494.00	2,494.00	
Test001	1 - B1	N/A	0.00	0.00	Fitting: Below standard range and out of invertable range
Test002	1 - B2	N/A	2.00	2.00	

Quantitative Analyte Results for D7 - Mouse IL-12p70

Name	Plate Position	Nominal CC	Fitted CC	Final CC	Message
Test003	1 - B3	N/A	0.00	0.00	Fitting: Below standard range and out of invertable range
Test004	1 - B4	N/A	0.00	0.00	Fitting: Below standard range and out of invertable range
Test005	1 - B5	N/A	3.60	3.60	
Test006	1 - B6	N/A	2.52	2.52	
Test007	1 - B7	N/A	4.58	4.58	
Test008	1 - B8	N/A	3.07	3.07	
Test009	1 - B9	N/A	4.23	4.23	
Test010	1 - B10	N/A	4.41	4.41	
Test011	1 - B11	N/A	3.54	3.54	
Test012	1 - B12	N/A	3.54	3.54	
Test013	1 - C1	N/A	1.12	1.12	
Test014	1 - C2	N/A	4.69	4.69	
Test015	1 - C3	N/A	1.59	1.59	
Test016	1 - C4	N/A	3.18	3.18	
Test017	1 - C5	N/A	0.00	0.00	Fitting: Below standard range and out of invertable range
Test018	1 - C6	N/A	0.00	0.00	Fitting: Below standard range and out of invertable range
Test019	1 - C7	N/A	0.94	0.94	
Test020	1 - C8	N/A	0.00	0.00	Fitting: Below standard range and out of invertable range
Test021	1 - C9	N/A	0.00	0.00	Fitting: Below standard range and out of invertable range
Test022	1 - C10	N/A	0.00	0.00	Fitting: Below standard range and out of invertable range
Test023	1 - C11	N/A	0.00	0.00	Fitting: Below standard range and out of invertable range
Test024	1 - C12	N/A	0.00	0.00	Fitting: Below standard range and out of invertable range
Test025	1 - D1	N/A	2.58	2.58	
Test026	1 - D2	N/A	0.00	0.00	Fitting: Below standard range and out of invertable range
Test027	1 - D3	N/A	0.00	0.00	Fitting: Below standard range and out of invertable range
Test028	1 - D4	N/A	0.00	0.00	Fitting: Below standard range and out of invertable range
Test029	1 - D5	N/A	0.00	0.00	Fitting: Below standard range and out of invertable range
Test030	1 - D6	N/A	0.00	0.00	Fitting: Below standard range and out of invertable range
Test031	1 - D7	N/A	0.00	0.00	Fitting: Below standard range and out of invertable range
Test032	1 - D8	N/A	0.00	0.00	Fitting: Below standard range and out of invertable range
Test033	1 - D9	N/A	0.00	0.00	Fitting: Below standard range and out of invertable range
					[illegible]

Quantitative Analyte Results for D7 - Mouse IL-12p70

Name	Plate Position	Nominal CC	Fitted CC	Final CC	Message
Test034	1 - D10	N/A	0.00	0.00	Fitting: Below standard range and out of invertable range
Test035	1 - D11	N/A	0.00	0.00	Fitting: Below standard range and out of invertable range
Test036	1 - D12	N/A	0.00	0.00	Fitting: Below standard range and out of invertable range
Test037	1 - E1	N/A	0.00	0.00	Fitting: Below standard range and out of invertable range
Test038	1 - E2	N/A	0.00	0.00	Fitting: Below standard range and out of invertable range
Test039	1 - E3	N/A	0.00	0.00	Fitting: Below standard range and out of invertable range
Test040	1 - E4	N/A	0.00	0.00	Fitting: Below standard range and out of invertable range
Test041	1 - E5	N/A	0.00	0.00	Fitting: Below standard range and out of invertable range
Test042	1 - E6	N/A	0.00	0.00	Fitting: Below standard range and out of invertable range
Test044	1 - E7	N/A	0.00	0.00	Fitting: Below standard range and out of invertable range

Results Statistics for B6 - Mouse IL-21

Name	Plate Pos.	Clust.	Event #	MFI	SD	CV	Dilution
Std001	1 - A1	Auto	257	385.00	433.66	80.82 %	1.00
Std002	1 - A2	Auto	259	1,319.00	565.61	37.15 %	1.00
Std003	1 - A3	Auto	255	2,009.00	624.17	27.46 %	1.00
Std004	1 - A4	Auto	284	2,697.00	514.28	18.82 %	1.00
Std005	1 - A5	Auto	278	5,465.00	1,175.33	21.70 %	1.00
Std006	1 - A6	Auto	279	11,608.00	2,268.38	19.23 %	1.00
Std007	1 - A7	Auto	289	19,916.00	4,451.14	19.84 %	1.00
Std008	1 - A8	Auto	269	33,416.00	6,753.24	19.99 %	1.00
Std009	1 - A9	Auto	310	53,156.00	8,737.89	16.15 %	1.00
Std010	1 - A10	Auto	265	65,089.00	10,310.74	16.28 %	1.00
Test001	1 - B1	Auto	275	384.00	429.21	81.46 %	1.00
Test002	1 - B2	Auto	281	434.00	453.30	78.43 %	1.00
Test003	1 - B3	Auto	265	492.00	425.51	72.97 %	1.00
Test004	1 - B4	Auto	248	363.00	495.56	82.80 %	1.00
Test005	1 - B5	Auto	261	449.00	299.86	57.88 %	1.00
Test006	1 - B6	Auto	299	445.00	299.49	56.86 %	1.00
Test007	1 - B7	Auto	258	428.00	404.38	69.31 %	1.00
Test008	1 - B8	Auto	275	428.00	404.75	66.61 %	1.00
Test009	1 - B9	Auto	186	446.00	273.54	53.85 %	1.00
Test010	1 - B10	Auto	228	641.00	443.67	58.78 %	1.00
Test011	1 - B11	Auto	254	485.00	479.62	73.35 %	1.00
Test012	1 - B12	Auto	237	503.00	343.96	62.83 %	1.00

Results Statistics for B6 - Mouse IL-21

Name	Plate Pos.	Clust.	Event #	MFI	SD	CV	Dilution
Test013	1 - C1	Auto	250	502.00	351.93	58.59 %	1.00
Test014	1 - C2	Auto	234	465.00	385.85	67.12 %	1.00
Test015	1 - C3	Auto	265	574.00	483.70	72.96 %	1.00
Test016	1 - C4	Auto	253	458.00	452.56	75.94 %	1.00
Test017	1 - C5	Auto	287	508.00	518.17	74.05 %	1.00
Test018	1 - C6	Auto	204	411.00	555.23	84.41 %	1.00
Test019	1 - C7	Auto	264	471.00	518.91	78.12 %	1.00
Test020	1 - C8	Auto	279	441.00	424.76	72.70 %	1.00
Test021	1 - C9	Auto	268	442.00	461.09	75.14 %	1.00
Test022	1 - C10	Auto	265	410.00	339.14	68.44 %	1.00
Test023	1 - C11	Auto	263	409.00	177.17	41.52 %	1.00
Test024	1 - C12	Auto	253	389.00	209.42	44.85 %	1.00
Test025	1 - D1	Auto	256	396.00	144.18	32.76 %	1.00
Test026	1 - D2	Auto	248	351.00	290.77	69.81 %	1.00
Test027	1 - D3	Auto	276	370.00	280.40	63.35 %	1.00
Test028	1 - D4	Manual	233	405.00	405.12	71.09 %	1.00
Test029	1 - D5	Manual	276	403.00	305.42	62.02 %	1.00
Test030	1 - D6	Auto	297	384.00	327.65	62.58 %	1.00
Test031	1 - D7	Auto	245	397.00	199.41	50.03 %	1.00
Test032	1 - D8	Auto	300	407.00	297.63	58.66 %	1.00
Test033	1 - D9	Auto	236	356.00	273.35	60.32 %	1.00
Test034	1 - D10	Manual	259	407.00	296.52	62.94 %	1.00
Test035	1 - D11	Auto	266	356.00	170.68	45.18 %	1.00
Test036	1 - D12	Auto	258	371.00	283.92	60.43 %	1.00
Test037	1 - E1	Auto	275	397.00	274.28	55.97 %	1.00
Test038	1 - E2	Auto	287	385.00	249.08	57.60 %	1.00
Test039	1 - E3	Auto	278	350.00	313.38	64.36 %	1.00
Test040	1 - E4	Auto	239	401.00	144.55	35.88 %	1.00
Test041	1 - E5	Auto	266	396.00	283.55	57.13 %	1.00
Test042	1 - E6	Auto	250	364.00	238.88	57.62 %	1.00
Test044	1 - E7	Auto	270	381.00	310.05	63.81 %	1.00

Quantitative Analyte Results for B6 - Mouse IL-21

Name	Plate Position	Nominal CC	Fitted CC	Final CC	Message
Std001	1 - A1	0.00	0.00	0.00	Fitting: Below standard range and out of invertable range
Std002	1 - A2	10.00	12.31	12.31	
Std003	1 - A3	20.00	21.35	21.35	
Std004	1 - A4	40.00	30.59	30.59	
Std005	1 - A5	80.00	70.20	70.20	
Std006	1 - A6	156.00	172.27	172.27	
Std007	1 - A7	312.50	341.58	341.58	
Std008	1 - A8	625.00	696.82	696.82	

Quantitative Analyte Results for B6 - Mouse IL-21

Name	Plate Position	Nominal CC	Fitted CC	Final CC	Message
Std009	1 - A9	1,250.00	1,414.41	1,414.41	
Std010	1 - A10	2,500.00	1,978.12	1,978.12	
Test001	1 - B1	N/A	0.00	0.00	Fitting: Below standard range and out of invertable range
Test002	1 - B2	N/A	0.74	0.74	
Test003	1 - B3	N/A	1.60	1.60	
Test004	1 - B4	N/A	0.00	0.00	Fitting: Below standard range and out of invertable range
Test005	1 - B5	N/A	0.98	0.98	
Test006	1 - B6	N/A	0.92	0.92	
Test007	1 - B7	N/A	0.64	0.64	
Test008	1 - B8	N/A	0.64	0.64	
Test009	1 - B9	N/A	0.93	0.93	
Test010	1 - B10	N/A	3.58	3.58	
Test011	1 - B11	N/A	1.50	1.50	
Test012	1 - B12	N/A	1.75	1.75	
Test013	1 - C1	N/A	1.74	1.74	
Test014	1 - C2	N/A	1.22	1.22	
Test015	1 - C3	N/A	2.70	2.70	
Test016	1 - C4	N/A	1.11	1.11	
Test017	1 - C5	N/A	1.82	1.82	
Test018	1 - C6	N/A	0.33	0.33	
Test019	1 - C7	N/A	1.30	1.30	
Test020	1 - C8	N/A	0.85	0.85	
Test021	1 - C9	N/A	0.87	0.87	
Test022	1 - C10	N/A	0.31	0.31	
Test023	1 - C11	N/A	0.28	0.28	
Test024	1 - C12	N/A	0.00	0.00	Fitting: Below standard range and out of invertable range
Test025	1 - D1	N/A	0.00	0.00	Fitting: Below standard range and out of invertable range
Test026	1 - D2	N/A	0.00	0.00	Fitting: Below standard range and out of invertable range
Test027	1 - D3	N/A	0.00	0.00	Fitting: Below standard range and out of invertable range
Test028	1 - D4	N/A	0.19	0.19	
Test029	1 - D5	N/A	0.14	0.14	
Test030	1 - D6	N/A	0.00	0.00	Fitting: Below standard range and out of invertable range
Test031	1 - D7	N/A	0.00	0.00	
Test032	1 - D8	N/A	0.24	0.24	
Test033	1 - D9	N/A	0.00	0.00	Fitting: Below standard range and out of invertable range
Test034	1 - D10	N/A	0.24	0.24	
Test035	1 - D11	N/A	0.00	0.00	Fitting: Below standard range and out of invertable range
[illegible]	[illegible]	[illegible]	[illegible]	[illegible]	Fitting: Below standard range [illegible]

Quantitative Analyte Results for B6 - Mouse IL-21

Name	Plate Position	Nominal CC	Fitted CC	Final CC	Message
Test036	1 - D12	N/A	0.00	0.00	Fitting: Below standard range and out of invertable range
Test037	1 - E1	N/A	0.00	0.00	
Test038	1 - E2	N/A	0.00	0.00	Fitting: Below standard range and out of invertable range
Test039	1 - E3	N/A	0.00	0.00	Fitting: Below standard range and out of invertable range
Test040	1 - E4	N/A	0.08	0.08	
Test041	1 - E5	N/A	0.00	0.00	Fitting: Below standard range and out of invertable range
Test042	1 - E6	N/A	0.00	0.00	Fitting: Below standard range and out of invertable range
Test044	1 - E7	N/A	0.00	0.00	Fitting: Below standard range and out of invertable range

Results Statistics for A5 - Mouse IL-2

Name	Plate Pos.	Clust.	Event #	MFI	SD	CV	Dilution
Std001	1 - A1	Auto	274	835.00	524.10	47.41 %	1.00
Std002	1 - A2	Auto	237	4,039.00	795.04	16.81 %	1.00
Std003	1 - A3	Auto	241	6,602.00	1,332.12	17.77 %	1.00
Std004	1 - A4	Auto	374	9,937.00	1,805.99	15.94 %	1.00
Std005	1 - A5	Auto	257	20,974.00	4,224.67	18.03 %	1.00
Std006	1 - A6	Auto	251	43,260.00	7,249.91	16.80 %	1.00
Std007	1 - A7	Auto	280	76,339.00	16,047.85	17.93 %	1.00
Std008	1 - A8	Auto	280	142,951.00	27,722.77	17.67 %	1.00
Std009	1 - A9	Auto	268	265,754.00	44,102.35	15.08 %	1.00
Std010	1 - A10	Auto	291	393,580.00	57,123.84	12.43 %	1.00
Test001	1 - B1	Auto	258	894.00	568.39	54.34 %	1.00
Test002	1 - B2	Auto	253	831.00	465.54	47.09 %	1.00
Test003	1 - B3	Auto	278	883.00	419.02	40.58 %	1.00
Test004	1 - B4	Auto	273	869.00	507.05	47.73 %	1.00
Test005	1 - B5	Auto	256	919.00	294.30	26.91 %	1.00
Test006	1 - B6	Auto	273	900.00	265.39	27.61 %	1.00
Test007	1 - B7	Auto	268	844.00	351.75	38.66 %	1.00
Test008	1 - B8	Auto	236	808.00	359.16	36.79 %	1.00
Test009	1 - B9	Auto	291	865.00	277.99	28.81 %	1.00
Test010	1 - B10	Auto	271	874.00	372.87	37.19 %	1.00
Test011	1 - B11	Auto	262	893.00	427.36	43.93 %	1.00
Test012	1 - B12	Auto	293	857.00	410.68	41.25 %	1.00
Test013	1 - C1	Auto	289	921.00	343.59	34.01 %	1.00
Test014	1 - C2	Auto	272	851.00	369.72	36.47 %	1.00
Test015	1 - C3	Auto	273	807.00	528.18	52.00 %	1.00
Test016	1 - C4	Auto	288	827.00	523.17	50.01 %	1.00
Test017	1 - C5	Auto	271	841.00	475.91	45.68 %	1.00
Test018	1 - C6	Auto	250	743.00	471.10	51.03 %	1.00

Results Statistics for A5 - Mouse IL-2

Name	Plate Pos.	Clust.	Event #	MFI	SD	CV	Dilution
Test019	1 - C7	Auto	267	872.00	492.96	48.24 %	1.00
Test020	1 - C8	Auto	273	868.00	366.57	36.07 %	1.00
Test021	1 - C9	Auto	255	804.00	381.77	44.78 %	1.00
Test022	1 - C10	Auto	232	734.00	364.35	41.31 %	1.00
Test023	1 - C11	Auto	273	737.00	199.04	24.32 %	1.00
Test024	1 - C12	Auto	259	756.00	161.60	21.23 %	1.00
Test025	1 - D1	Auto	247	754.00	156.41	18.63 %	1.00
Test026	1 - D2	Auto	265	678.00	295.41	36.41 %	1.00
Test027	1 - D3	Auto	265	699.00	283.55	32.33 %	1.00
Test028	1 - D4	Manual	228	727.00	361.20	41.04 %	1.00
Test029	1 - D5	Manual	240	765.00	293.37	33.35 %	1.00
Test030	1 - D6	Auto	251	717.00	286.88	35.50 %	1.00
Test031	1 - D7	Auto	238	724.00	223.87	27.24 %	1.00
Test032	1 - D8	Auto	291	719.00	263.90	30.85 %	1.00
Test033	1 - D9	Auto	250	688.00	236.85	28.63 %	1.00
Test034	1 - D10	Manual	241	738.00	338.77	36.35 %	1.00
Test035	1 - D11	Auto	225	700.00	190.14	24.45 %	1.00
Test036	1 - D12	Auto	269	735.00	279.10	35.98 %	1.00
Test037	1 - E1	Auto	253	748.00	204.23	25.78 %	1.00
Test038	1 - E2	Auto	256	742.00	262.79	30.68 %	1.00
Test039	1 - E3	Auto	271	783.00	275.76	30.85 %	1.00
Test040	1 - E4	Auto	286	748.00	177.17	20.16 %	1.00
Test041	1 - E5	Auto	257	716.00	283.18	31.18 %	1.00
Test042	1 - E6	Auto	256	772.00	260.20	30.90 %	1.00
Test044	1 - E7	Auto	266	754.00	267.05	33.18 %	1.00

Quantitative Analyte Results for A5 - Mouse IL-2

Name	Plate Position	Nominal CC	Fitted CC	Final CC	Message
Std001	1 - A1	0.00	0.00	0.00	Fitting: Below standard range
Std002	1 - A2	10.00	11.17	11.17	
Std003	1 - A3	20.00	20.69	20.69	
Std004	1 - A4	40.00	33.34	33.34	
Std005	1 - A5	80.00	76.56	76.56	
Std006	1 - A6	156.00	168.88	168.88	
Std007	1 - A7	312.50	316.65	316.65	
Std008	1 - A8	625.00	648.06	648.06	
Std009	1 - A9	1,250.00	1,363.20	1,363.20	
Std010	1 - A10	2,500.00	2,238.24	2,238.24	
Test001	1 - B1	N/A	0.15	0.15	
Test002	1 - B2	N/A	0.00	0.00	Fitting: Below standard range
Test003	1 - B3	N/A	0.12	0.12	
Test004	1 - B4	N/A	0.07	0.07	
Test005	1 - B5	N/A	0.23	0.23	

Quantitative Analyte Results for A5 - Mouse IL-2

Name	Plate Position	Nominal CC	Fitted CC	Final CC	Message
Test006	1 - B6	N/A	0.17	0.17	
Test007	1 - B7	N/A	0.00	0.00	
Test008	1 - B8	N/A	0.00	0.00	Fitting: Below standard range
Test009	1 - B9	N/A	0.06	0.06	
Test010	1 - B10	N/A	0.09	0.09	
Test011	1 - B11	N/A	0.15	0.15	
Test012	1 - B12	N/A	0.04	0.04	
Test013	1 - C1	N/A	0.24	0.24	
Test014	1 - C2	N/A	0.02	0.02	
Test015	1 - C3	N/A	0.00	0.00	Fitting: Below standard range
Test016	1 - C4	N/A	0.00	0.00	Fitting: Below standard range
Test017	1 - C5	N/A	0.00	0.00	
Test018	1 - C6	N/A	0.00	0.00	Fitting: Below standard range
Test019	1 - C7	N/A	0.08	0.08	
Test020	1 - C8	N/A	0.07	0.07	
Test021	1 - C9	N/A	0.00	0.00	Fitting: Below standard range
Test022	1 - C10	N/A	0.00	0.00	Fitting: Below standard range
Test023	1 - C11	N/A	0.00	0.00	Fitting: Below standard range
Test024	1 - C12	N/A	0.00	0.00	Fitting: Below standard range
Test025	1 - D1	N/A	0.00	0.00	Fitting: Below standard range
Test026	1 - D2	N/A	0.00	0.00	Fitting: Below standard range
Test027	1 - D3	N/A	0.00	0.00	Fitting: Below standard range
Test028	1 - D4	N/A	0.00	0.00	Fitting: Below standard range
Test029	1 - D5	N/A	0.00	0.00	Fitting: Below standard range
Test030	1 - D6	N/A	0.00	0.00	Fitting: Below standard range
Test031	1 - D7	N/A	0.00	0.00	Fitting: Below standard range
Test032	1 - D8	N/A	0.00	0.00	Fitting: Below standard range
Test033	1 - D9	N/A	0.00	0.00	Fitting: Below standard range
Test034	1 - D10	N/A	0.00	0.00	Fitting: Below standard range
Test035	1 - D11	N/A	0.00	0.00	Fitting: Below standard range
Test036	1 - D12	N/A	0.00	0.00	Fitting: Below standard range
Test037	1 - E1	N/A	0.00	0.00	Fitting: Below standard range
Test038	1 - E2	N/A	0.00	0.00	Fitting: Below standard range
Test039	1 - E3	N/A	0.00	0.00	Fitting: Below standard range
Test040	1 - E4	N/A	0.00	0.00	Fitting: Below standard range
Test041	1 - E5	N/A	0.00	0.00	Fitting: Below standard range
Test042	1 - E6	N/A	0.00	0.00	Fitting: Below standard range
Test044	1 - E7	N/A	0.00	0.00	Fitting: Below standard range

Results Statistics for A7 - Mouse IL-4

Name	Plate Pos.	Clust.	Event #	MFI	SD	CV	Dilution
Std001	1 - A1	Auto	238	691.00	505.01	60.67 %	1.00
Std002	1 - A2	Auto	264	1,907.00	550.79	27.70 %	1.00

Results Statistics for A7 - Mouse IL-4

Name	Plate Pos.	Clust.	Event #	MFI	SD	CV	Dilution
Std003	1 - A3	Auto	242	2,976.00	880.48	26.49 %	1.00
Std004	1 - A4	Auto	261	4,410.00	962.58	20.61 %	1.00
Std005	1 - A5	Auto	276	10,805.00	2,680.17	24.43 %	1.00
Std006	1 - A6	Auto	263	24,258.00	5,094.95	20.44 %	1.00
Std007	1 - A7	Auto	283	47,356.00	12,341.16	26.86 %	1.00
Std008	1 - A8	Auto	256	99,188.00	23,978.09	23.75 %	1.00
Std009	1 - A9	Auto	231	181,524.00	40,974.62	20.29 %	1.00
Std010	1 - A10	Auto	283	320,860.00	59,687.99	17.34 %	1.00
Test001	1 - B1	Auto	287	610.00	470.73	65.17 %	1.00
Test002	1 - B2	Auto	268	620.00	464.24	63.04 %	1.00
Test003	1 - B3	Auto	267	646.00	438.11	57.90 %	1.00
Test004	1 - B4	Auto	263	664.00	453.68	59.03 %	1.00
Test005	1 - B5	Auto	250	669.00	277.80	39.64 %	1.00
Test006	1 - B6	Auto	264	671.00	302.27	39.55 %	1.00
Test007	1 - B7	Auto	256	641.00	387.33	51.90 %	1.00
Test008	1 - B8	Auto	257	599.00	394.00	53.48 %	1.00
Test009	1 - B9	Auto	247	657.00	293.55	39.95 %	1.00
Test010	1 - B10	Auto	272	763.00	407.72	49.48 %	1.00
Test011	1 - B11	Auto	209	641.00	452.56	61.42 %	1.00
Test012	1 - B12	Auto	247	689.00	440.33	53.17 %	1.00
Test013	1 - C1	Auto	252	675.00	337.11	42.94 %	1.00
Test014	1 - C2	Auto	227	693.00	403.27	51.11 %	1.00
Test015	1 - C3	Auto	227	622.00	522.62	66.70 %	1.00
Test016	1 - C4	Auto	248	571.00	477.03	65.58 %	1.00
Test017	1 - C5	Auto	277	588.00	480.36	62.23 %	1.00
Test018	1 - C6	Auto	270	664.00	581.36	66.50 %	1.00
Test019	1 - C7	Auto	234	750.00	545.23	60.19 %	1.00
Test020	1 - C8	Auto	272	620.00	380.29	53.79 %	1.00
Test021	1 - C9	Auto	255	564.00	402.53	58.16 %	1.00
Test022	1 - C10	Auto	295	567.00	323.95	51.42 %	1.00
Test023	1 - C11	Auto	304	589.00	167.90	28.74 %	1.00
Test024	1 - C12	Auto	291	576.00	165.31	26.98 %	1.00
Test025	1 - D1	Auto	319	583.00	150.48	23.53 %	1.00
Test026	1 - D2	Auto	283	517.00	296.52	49.47 %	1.00
Test027	1 - D3	Auto	298	541.00	298.19	47.57 %	1.00
Test028	1 - D4	Manual	332	548.00	353.97	53.19 %	1.00
Test029	1 - D5	Manual	300	493.00	242.96	45.92 %	1.00
Test030	1 - D6	Auto	324	514.00	303.01	51.50 %	1.00
Test031	1 - D7	Auto	296	569.00	231.84	34.54 %	1.00
Test032	1 - D8	Auto	266	540.00	239.81	40.94 %	1.00
Test033	1 - D9	Auto	301	498.00	231.66	41.30 %	1.00
Test034	1 - D10	Manual	322	507.00	287.07	44.38 %	1.00
Test035	1 - D11	Auto	291	560.00	179.39	30.02 %	1.00
Test036	1 - D12	Auto	313	600.00	290.22	41.59 %	1.00
Test037	1 - E1	Auto	276	577.00	209.60	35.58 %	1.00

Results Statistics for A7 - Mouse IL-4

Name	Plate Pos.	Clust.	Event #	MFI	SD	CV	Dilution
Test038	1 - E2	Auto	303	564.00	269.83	39.42 %	1.00
Test039	1 - E3	Auto	302	570.00	237.40	39.34 %	1.00
Test040	1 - E4	Auto	266	590.00	174.76	26.01 %	1.00
Test041	1 - E5	Auto	285	616.00	250.19	35.62 %	1.00
Test042	1 - E6	Auto	329	555.00	228.32	38.71 %	1.00
Test044	1 - E7	Auto	300	553.00	275.21	43.75 %	1.00

Quantitative Analyte Results for A7 - Mouse IL-4

Name	Plate Position	Nominal CC	Fitted CC	Final CC	Message
Std001	1 - A1	0.00	0.00	0.00	Fitting: Below standard range and out of invertable range
Std002	1 - A2	10.00	11.89	11.89	
Std003	1 - A3	20.00	20.92	20.92	
Std004	1 - A4	40.00	32.31	32.31	
Std005	1 - A5	80.00	78.64	78.64	
Std006	1 - A6	156.00	168.02	168.02	
Std007	1 - A7	312.50	316.23	316.23	
Std008	1 - A8	625.00	655.85	655.85	
Std009	1 - A9	1,250.00	1,241.86	1,241.86	
Std010	1 - A10	2,500.00	2,386.61	2,386.61	
Test001	1 - B1	N/A	0.00	0.00	Fitting: Below standard range and out of invertable range
Test002	1 - B2	N/A	0.00	0.00	Fitting: Below standard range and out of invertable range
Test003	1 - B3	N/A	0.00	0.00	Fitting: Below standard range and out of invertable range
Test004	1 - B4	N/A	0.00	0.00	Fitting: Below standard range and out of invertable range
Test005	1 - B5	N/A	0.00	0.00	Fitting: Below standard range and out of invertable range
Test006	1 - B6	N/A	0.00	0.00	Fitting: Below standard range and out of invertable range
Test007	1 - B7	N/A	0.00	0.00	Fitting: Below standard range and out of invertable range
Test008	1 - B8	N/A	0.00	0.00	Fitting: Below standard range and out of invertable range
Test009	1 - B9	N/A	0.00	0.00	Fitting: Below standard range and out of invertable range
Test010	1 - B10	N/A	0.96	0.96	
Test011	1 - B11	N/A	0.00	0.00	Fitting: Below standard range and out of invertable range
Test012	1 - B12	N/A	0.00	0.00	Fitting: Below standard range and out of invertable range
Test013	1 - C1	N/A	0.00	0.00	Fitting: Below standard range and out of invertable range

Quantitative Analyte Results for A7 - Mouse IL-4

Name	Plate Position	Nominal CC	Fitted CC	Final CC	Message
Test014	1 - C2	N/A	0.00	0.00	Fitting: Below standard range and out of invertable range
Test015	1 - C3	N/A	0.00	0.00	Fitting: Below standard range and out of invertable range
Test016	1 - C4	N/A	0.00	0.00	Fitting: Below standard range and out of invertable range
Test017	1 - C5	N/A	0.00	0.00	Fitting: Below standard range and out of invertable range
Test018	1 - C6	N/A	0.00	0.00	Fitting: Below standard range and out of invertable range
Test019	1 - C7	N/A	0.78	0.78	
Test020	1 - C8	N/A	0.00	0.00	Fitting: Below standard range and out of invertable range
Test021	1 - C9	N/A	0.00	0.00	Fitting: Below standard range and out of invertable range
Test022	1 - C10	N/A	0.00	0.00	Fitting: Below standard range and out of invertable range
Test023	1 - C11	N/A	0.00	0.00	Fitting: Below standard range and out of invertable range
Test024	1 - C12	N/A	0.00	0.00	Fitting: Below standard range and out of invertable range
Test025	1 - D1	N/A	0.00	0.00	Fitting: Below standard range and out of invertable range
Test026	1 - D2	N/A	0.00	0.00	Fitting: Below standard range and out of invertable range
Test027	1 - D3	N/A	0.00	0.00	Fitting: Below standard range and out of invertable range
Test028	1 - D4	N/A	0.00	0.00	Fitting: Below standard range and out of invertable range
Test029	1 - D5	N/A	0.00	0.00	Fitting: Below standard range and out of invertable range
Test030	1 - D6	N/A	0.00	0.00	Fitting: Below standard range and out of invertable range
Test031	1 - D7	N/A	0.00	0.00	Fitting: Below standard range and out of invertable range
Test032	1 - D8	N/A	0.00	0.00	Fitting: Below standard range and out of invertable range
Test033	1 - D9	N/A	0.00	0.00	Fitting: Below standard range and out of invertable range
Test034	1 - D10	N/A	0.00	0.00	Fitting: Below standard range and out of invertable range
Test035	1 - D11	N/A	0.00	0.00	Fitting: Below standard range and out of invertable range
Test036	1 - D12	N/A	0.00	0.00	Fitting: Below standard range and out of invertable range
Test037	1 - E1	N/A	0.00	0.00	Fitting: Below standard range and out of invertable range
Test038	1 - E2	N/A	0.00	0.00	Fitting: Below standard range and out of invertable range
Test039	1 - E3	N/A	0.00	0.00	Fitting: Below standard range

Quantitative Analyte Results for A7 - Mouse IL-4

Name	Plate Position	Nominal CC	Fitted CC	Final CC	Message
[illegible]	[illegible]	[illegible]	[illegible]	[illegible]	[illegible] and out of invertable range
Test040	1 - E4	N/A	0.00	0.00	Fitting: Below standard range and out of invertable range
Test041	1 - E5	N/A	0.00	0.00	Fitting: Below standard range and out of invertable range
Test042	1 - E6	N/A	0.00	0.00	Fitting: Below standard range and out of invertable range
Test044	1 - E7	N/A	0.00	0.00	Fitting: Below standard range and out of invertable range

Results Statistics for A6 - Mouse IL-5

Name	Plate Pos.	Clust.	Event #	MFI	SD	CV	Dilution
Std001	1 - A1	Auto	281	621.00	472.58	63.82 %	1.00
Std002	1 - A2	Auto	298	1,769.00	507.42	26.39 %	1.00
Std003	1 - A3	Auto	321	2,844.00	717.21	25.29 %	1.00
Std004	1 - A4	Auto	486	4,421.00	1,018.18	22.19 %	1.00
Std005	1 - A5	Auto	299	10,242.00	2,418.12	22.00 %	1.00
Std006	1 - A6	Auto	280	22,908.00	5,302.33	21.39 %	1.00
Std007	1 - A7	Auto	306	44,230.00	11,191.22	22.59 %	1.00
Std008	1 - A8	Auto	416	93,487.00	23,535.16	23.55 %	1.00
Std009	1 - A9	Auto	300	179,905.00	35,816.84	18.00 %	1.00
Std010	1 - A10	Auto	293	288,218.00	53,491.10	15.56 %	1.00
Test001	1 - B1	Auto	273	489.00	497.41	75.96 %	1.00
Test002	1 - B2	Auto	285	546.00	488.52	68.21 %	1.00
Test003	1 - B3	Auto	295	656.00	428.47	61.67 %	1.00
Test004	1 - B4	Auto	285	541.00	512.61	70.67 %	1.00
Test005	1 - B5	Auto	257	610.00	237.59	37.13 %	1.00
Test006	1 - B6	Auto	269	537.00	244.26	47.07 %	1.00
Test007	1 - B7	Auto	299	555.00	402.53	61.20 %	1.00
Test008	1 - B8	Auto	302	504.00	404.75	61.48 %	1.00
Test009	1 - B9	Auto	288	604.00	274.28	40.26 %	1.00
Test010	1 - B10	Auto	291	552.00	389.18	57.27 %	1.00
Test011	1 - B11	Auto	364	491.00	432.18	68.39 %	1.00
Test012	1 - B12	Auto	344	558.00	366.02	60.02 %	1.00
Test013	1 - C1	Auto	283	506.00	334.33	53.21 %	1.00
Test014	1 - C2	Auto	320	540.00	316.16	53.60 %	1.00
Test015	1 - C3	Auto	299	530.00	462.57	70.63 %	1.00
Test016	1 - C4	Auto	288	618.00	491.11	69.15 %	1.00
Test017	1 - C5	Auto	297	517.00	446.63	67.30 %	1.00
Test018	1 - C6	Auto	274	346.00	483.88	87.82 %	1.00
Test019	1 - C7	Auto	307	476.00	447.75	68.11 %	1.00
Test020	1 - C8	Auto	293	502.00	391.41	58.23 %	1.00
Test021	1 - C9	Auto	293	528.00	453.68	63.32 %	1.00
Test022	1 - C10	Auto	249	463.00	345.08	61.94 %	1.00

Results Statistics for A6 - Mouse IL-5

Name	Plate Pos.	Clust.	Event #	MFI	SD	CV	Dilution
Test023	1 - C11	Auto	290	463.00	161.60	31.85 %	1.00
Test024	1 - C12	Auto	279	462.00	167.53	33.28 %	1.00
Test025	1 - D1	Auto	277	487.00	155.30	27.25 %	1.00
Test026	1 - D2	Auto	271	398.00	282.44	58.76 %	1.00
Test027	1 - D3	Auto	320	403.00	251.12	55.52 %	1.00
Test028	1 - D4	Manual	273	418.00	331.36	61.34 %	1.00
Test029	1 - D5	Manual	272	459.00	285.22	54.29 %	1.00
Test030	1 - D6	Auto	262	367.00	255.38	60.37 %	1.00
Test031	1 - D7	Auto	274	475.00	204.97	36.70 %	1.00
Test032	1 - D8	Auto	275	423.00	252.78	51.14 %	1.00
Test033	1 - D9	Auto	287	469.00	223.87	43.15 %	1.00
Test034	1 - D10	Manual	241	446.00	259.46	53.36 %	1.00
Test035	1 - D11	Auto	294	428.00	157.53	35.56 %	1.00
Test036	1 - D12	Auto	394	451.00	279.10	52.91 %	1.00
Test037	1 - E1	Auto	286	464.00	217.94	41.76 %	1.00
Test038	1 - E2	Auto	280	465.00	271.50	53.96 %	1.00
Test039	1 - E3	Auto	286	462.00	277.06	50.48 %	1.00
Test040	1 - E4	Auto	280	482.00	160.68	29.84 %	1.00
Test041	1 - E5	Auto	270	465.00	261.68	50.14 %	1.00
Test042	1 - E6	Auto	245	469.00	275.02	51.20 %	1.00
Test044	1 - E7	Auto	298	444.00	271.69	55.72 %	1.00

Quantitative Analyte Results for A6 - Mouse IL-5

Name	Plate Position	Nominal CC	Fitted CC	Final CC	Message
Std001	1 - A1	0.00	0.00	0.00	Fitting: Below standard range and out of invertable range
Std002	1 - A2	10.00	11.55	11.55	
Std003	1 - A3	20.00	20.89	20.89	
Std004	1 - A4	40.00	33.77	33.77	
Std005	1 - A5	80.00	77.47	77.47	
Std006	1 - A6	156.00	165.75	165.75	
Std007	1 - A7	312.50	310.60	310.60	
Std008	1 - A8	625.00	655.61	655.61	
Std009	1 - A9	1,250.00	1,324.10	1,324.10	
Std010	1 - A10	2,500.00	2,289.69	2,289.69	
Test001	1 - B1	N/A	0.00	0.00	Fitting: Below standard range and out of invertable range
Test002	1 - B2	N/A	0.00	0.00	Fitting: Below standard range and out of invertable range
Test003	1 - B3	N/A	0.45	0.45	
Test004	1 - B4	N/A	0.00	0.00	Fitting: Below standard range and out of invertable range
Test005	1 - B5	N/A	0.00	0.00	Fitting: Below standard range and out of invertable range

Quantitative Analyte Results for A6 - Mouse IL-5

Name	Plate Position	Nominal CC	Fitted CC	Final CC	Message
Test006	1 - B6	N/A	0.00	0.00	Fitting: Below standard range and out of invertable range
Test007	1 - B7	N/A	0.00	0.00	Fitting: Below standard range and out of invertable range
Test008	1 - B8	N/A	0.00	0.00	Fitting: Below standard range and out of invertable range
Test009	1 - B9	N/A	0.00	0.00	Fitting: Below standard range and out of invertable range
Test010	1 - B10	N/A	0.00	0.00	Fitting: Below standard range and out of invertable range
Test011	1 - B11	N/A	0.00	0.00	Fitting: Below standard range and out of invertable range
Test012	1 - B12	N/A	0.00	0.00	Fitting: Below standard range and out of invertable range
Test013	1 - C1	N/A	0.00	0.00	Fitting: Below standard range and out of invertable range
Test014	1 - C2	N/A	0.00	0.00	Fitting: Below standard range and out of invertable range
Test015	1 - C3	N/A	0.00	0.00	Fitting: Below standard range and out of invertable range
Test016	1 - C4	N/A	0.00	0.00	Fitting: Below standard range and out of invertable range
Test017	1 - C5	N/A	0.00	0.00	Fitting: Below standard range and out of invertable range
Test018	1 - C6	N/A	0.00	0.00	Fitting: Below standard range and out of invertable range
Test019	1 - C7	N/A	0.00	0.00	Fitting: Below standard range and out of invertable range
Test020	1 - C8	N/A	0.00	0.00	Fitting: Below standard range and out of invertable range
Test021	1 - C9	N/A	0.00	0.00	Fitting: Below standard range and out of invertable range
Test022	1 - C10	N/A	0.00	0.00	Fitting: Below standard range and out of invertable range
Test023	1 - C11	N/A	0.00	0.00	Fitting: Below standard range and out of invertable range
Test024	1 - C12	N/A	0.00	0.00	Fitting: Below standard range and out of invertable range
Test025	1 - D1	N/A	0.00	0.00	Fitting: Below standard range and out of invertable range
Test026	1 - D2	N/A	0.00	0.00	Fitting: Below standard range and out of invertable range
Test027	1 - D3	N/A	0.00	0.00	Fitting: Below standard range and out of invertable range
Test028	1 - D4	N/A	0.00	0.00	Fitting: Below standard range and out of invertable range
Test029	1 - D5	N/A	0.00	0.00	Fitting: Below standard range and out of invertable range
Test030	1 - D6	N/A	0.00	0.00	Fitting: Below standard range and out of invertable range

Quantitative Analyte Results for A6 - Mouse IL-5

Name	Plate Position	Nominal CC	Fitted CC	Final CC	Message
Test031	1 - D7	N/A	0.00	0.00	Fitting: Below standard range and out of invertable range
Test032	1 - D8	N/A	0.00	0.00	Fitting: Below standard range and out of invertable range
Test033	1 - D9	N/A	0.00	0.00	Fitting: Below standard range and out of invertable range
Test034	1 - D10	N/A	0.00	0.00	Fitting: Below standard range and out of invertable range
Test035	1 - D11	N/A	0.00	0.00	Fitting: Below standard range and out of invertable range
Test036	1 - D12	N/A	0.00	0.00	Fitting: Below standard range and out of invertable range
Test037	1 - E1	N/A	0.00	0.00	Fitting: Below standard range and out of invertable range
Test038	1 - E2	N/A	0.00	0.00	Fitting: Below standard range and out of invertable range
Test039	1 - E3	N/A	0.00	0.00	Fitting: Below standard range and out of invertable range
Test040	1 - E4	N/A	0.00	0.00	Fitting: Below standard range and out of invertable range
Test041	1 - E5	N/A	0.00	0.00	Fitting: Below standard range and out of invertable range
Test042	1 - E6	N/A	0.00	0.00	Fitting: Below standard range and out of invertable range
Test044	1 - E7	N/A	0.00	0.00	Fitting: Below standard range and out of invertable range

Results Statistics for B4 - Mouse IL-6

Name	Plate Pos.	Clust.	Event #	MFI	SD	CV	Dilution
Std001	1 - A1	Auto	277	600.00	447.00	62.25 %	1.00
Std002	1 - A2	Auto	288	1,645.00	543.93	29.82 %	1.00
Std003	1 - A3	Auto	264	2,574.00	621.21	23.84 %	1.00
Std004	1 - A4	Auto	250	3,477.00	701.83	19.51 %	1.00
Std005	1 - A5	Auto	295	7,174.00	1,404.02	22.95 %	1.00
Std006	1 - A6	Auto	270	14,048.00	2,855.12	18.29 %	1.00
Std007	1 - A7	Auto	259	23,206.00	4,725.79	18.93 %	1.00
Std008	1 - A8	Auto	259	42,667.00	9,503.47	18.94 %	1.00
Std009	1 - A9	Auto	247	78,826.00	16,086.95	19.27 %	1.00
Std010	1 - A10	Auto	270	147,840.00	28,752.62	17.97 %	1.00
Test001	1 - B1	Auto	286	691.00	484.07	58.15 %	1.00
Test002	1 - B2	Auto	289	690.00	419.58	52.34 %	1.00
Test003	1 - B3	Auto	278	864.00	416.98	44.56 %	1.00
Test004	1 - B4	Auto	305	816.00	551.16	54.86 %	1.00
Test005	1 - B5	Auto	278	896.00	292.44	29.55 %	1.00
Test006	1 - B6	Auto	303	996.00	304.67	27.97 %	1.00
Test007	1 - B7	Auto	321	792.00	396.97	44.85 %	1.00

Results Statistics for B4 - Mouse IL-6

Name	Plate Pos.	Clust.	Event #	MFI	SD	CV	Dilution
Test008	1 - B8	Auto	271	966.00	362.50	33.13 %	1.00
Test009	1 - B9	Auto	318	862.00	277.99	29.79 %	1.00
Test010	1 - B10	Auto	306	908.00	400.30	38.20 %	1.00
Test011	1 - B11	Auto	310	876.00	466.28	46.16 %	1.00
Test012	1 - B12	Auto	281	874.00	419.21	41.32 %	1.00
Test013	1 - C1	Auto	278	1,081.00	339.89	28.34 %	1.00
Test014	1 - C2	Auto	294	885.00	380.66	35.90 %	1.00
Test015	1 - C3	Auto	283	794.00	503.34	52.59 %	1.00
Test016	1 - C4	Auto	311	811.00	518.17	52.03 %	1.00
Test017	1 - C5	Auto	271	832.00	445.52	47.63 %	1.00
Test018	1 - C6	Auto	307	799.00	476.66	53.38 %	1.00
Test019	1 - C7	Auto	309	833.00	482.59	46.73 %	1.00
Test020	1 - C8	Auto	283	846.00	435.14	40.28 %	1.00
Test021	1 - C9	Auto	291	801.00	441.81	46.55 %	1.00
Test022	1 - C10	Auto	318	656.00	349.71	46.80 %	1.00
Test023	1 - C11	Auto	296	695.00	189.03	24.79 %	1.00
Test024	1 - C12	Auto	300	700.00	201.26	24.11 %	1.00
Test025	1 - D1	Auto	286	694.00	158.08	21.09 %	1.00
Test026	1 - D2	Auto	306	646.00	285.96	38.43 %	1.00
Test027	1 - D3	Auto	279	710.00	292.81	36.65 %	1.00
Test028	1 - D4	Manual	325	621.00	329.88	43.78 %	1.00
Test029	1 - D5	Manual	297	638.00	311.72	41.74 %	1.00
Test030	1 - D6	Auto	287	730.00	287.62	37.22 %	1.00
Test031	1 - D7	Auto	319	764.00	180.14	23.49 %	1.00
Test032	1 - D8	Auto	281	723.00	283.92	37.90 %	1.00
Test033	1 - D9	Auto	286	676.00	277.06	34.72 %	1.00
Test034	1 - D10	Manual	268	643.00	262.23	37.28 %	1.00
Test035	1 - D11	Auto	317	758.00	211.27	27.89 %	1.00
Test036	1 - D12	Auto	306	695.00	271.50	34.07 %	1.00
Test037	1 - E1	Auto	286	645.00	234.81	31.63 %	1.00
Test038	1 - E2	Auto	287	666.00	237.22	34.02 %	1.00
Test039	1 - E3	Auto	276	693.00	283.55	35.15 %	1.00
Test040	1 - E4	Auto	326	756.00	164.94	20.09 %	1.00
Test041	1 - E5	Auto	304	705.00	244.44	32.07 %	1.00
Test042	1 - E6	Auto	282	676.00	280.03	34.68 %	1.00
Test044	1 - E7	Auto	276	634.00	239.81	32.23 %	1.00

Quantitative Analyte Results for B4 - Mouse IL-6

Name	Plate Position	Nominal CC	Fitted CC	Final CC	Message
Std001	1 - A1	0.00	0.00	0.00	Fitting: Below standard range
Std002	1 - A2	10.00	10.55	10.55	
Std003	1 - A3	20.00	21.48	21.48	
Std004	1 - A4	40.00	32.66	32.66	

Quantitative Analyte Results for B4 - Mouse IL-6

Name	Plate Position	Nominal CC	Fitted CC	Final CC	Message
Std005	1 - A5	80.00	81.04	81.04	
Std006	1 - A6	156.00	176.23	176.23	
Std007	1 - A7	312.50	309.34	309.34	
Std008	1 - A8	625.00	609.80	609.80	
Std009	1 - A9	1,250.00	1,219.54	1,219.54	
Std010	1 - A10	2,500.00	2,538.58	2,538.58	
Test001	1 - B1	N/A	0.75	0.75	
Test002	1 - B2	N/A	0.74	0.74	
Test003	1 - B3	N/A	2.32	2.32	
Test004	1 - B4	N/A	1.87	1.87	
Test005	1 - B5	N/A	2.62	2.62	
Test006	1 - B6	N/A	3.60	3.60	
Test007	1 - B7	N/A	1.65	1.65	
Test008	1 - B8	N/A	3.30	3.30	
Test009	1 - B9	N/A	2.30	2.30	
Test010	1 - B10	N/A	2.74	2.74	
Test011	1 - B11	N/A	2.43	2.43	
Test012	1 - B12	N/A	2.41	2.41	
Test013	1 - C1	N/A	4.46	4.46	
Test014	1 - C2	N/A	2.52	2.52	
Test015	1 - C3	N/A	1.67	1.67	
Test016	1 - C4	N/A	1.82	1.82	
Test017	1 - C5	N/A	2.02	2.02	
Test018	1 - C6	N/A	1.71	1.71	
Test019	1 - C7	N/A	2.03	2.03	
Test020	1 - C8	N/A	2.15	2.15	
Test021	1 - C9	N/A	1.73	1.73	
Test022	1 - C10	N/A	0.44	0.44	
Test023	1 - C11	N/A	0.78	0.78	
Test024	1 - C12	N/A	0.82	0.82	
Test025	1 - D1	N/A	0.77	0.77	
Test026	1 - D2	N/A	0.36	0.36	
Test027	1 - D3	N/A	0.91	0.91	
Test028	1 - D4	N/A	0.14	0.14	
Test029	1 - D5	N/A	0.29	0.29	
Test030	1 - D6	N/A	1.09	1.09	
Test031	1 - D7	N/A	1.39	1.39	
Test032	1 - D8	N/A	1.03	1.03	
Test033	1 - D9	N/A	0.62	0.62	
Test034	1 - D10	N/A	0.33	0.33	
Test035	1 - D11	N/A	1.34	1.34	
Test036	1 - D12	N/A	0.78	0.78	
Test037	1 - E1	N/A	0.35	0.35	
Test038	1 - E2	N/A	0.53	0.53	
Test039	1 - E3	N/A	0.76	0.76	

Quantitative Analyte Results for B4 - Mouse IL-6

Name	Plate Position	Nominal CC	Fitted CC	Final CC	Message
[illegible]	[illegible]	[illegible]	[illegible]	[illegible]	
Test040	1 - E4	N/A	1.32	1.32	
Test041	1 - E5	N/A	0.87	0.87	
Test042	1 - E6	N/A	0.62	0.62	
Test044	1 - E7	N/A	0.25	0.25	

Results Statistics for A4 - Mouse IFN-γ

Name	Plate Pos.	Clust.	Event #	MFI	SD	CV	Dilution
Std001	1 - A1	Auto	293	696.00	484.44	56.71 %	1.00
Std002	1 - A2	Auto	287	2,428.00	589.33	21.79 %	1.00
Std003	1 - A3	Auto	313	3,958.00	755.01	17.98 %	1.00
Std004	1 - A4	Auto	408	5,645.00	1,214.43	19.43 %	1.00
Std005	1 - A5	Auto	268	12,400.00	2,366.23	17.59 %	1.00
Std006	1 - A6	Auto	290	25,314.00	4,210.21	15.55 %	1.00
Std007	1 - A7	Auto	306	43,950.00	8,085.17	17.15 %	1.00
Std008	1 - A8	Auto	326	84,991.00	17,389.60	17.57 %	1.00
Std009	1 - A9	Auto	294	161,004.00	31,183.34	19.37 %	1.00
Std010	1 - A10	Auto	300	282,529.00	49,223.43	16.00 %	1.00
Test001	1 - B1	Auto	289	695.00	551.16	59.76 %	1.00
Test002	1 - B2	Auto	263	635.00	464.80	60.41 %	1.00
Test003	1 - B3	Auto	303	759.00	458.86	51.97 %	1.00
Test004	1 - B4	Auto	311	630.00	438.11	57.69 %	1.00
Test005	1 - B5	Auto	287	672.00	292.81	37.34 %	1.00
Test006	1 - B6	Auto	272	680.00	297.08	38.52 %	1.00
Test007	1 - B7	Auto	324	671.00	361.38	47.19 %	1.00
Test008	1 - B8	Auto	264	712.00	399.75	46.77 %	1.00
Test009	1 - B9	Auto	265	697.00	309.12	37.24 %	1.00
Test010	1 - B10	Auto	258	746.00	368.43	43.59 %	1.00
Test011	1 - B11	Auto	297	716.00	477.40	56.66 %	1.00
Test012	1 - B12	Auto	286	662.00	406.60	52.22 %	1.00
Test013	1 - C1	Auto	285	741.00	361.01	40.27 %	1.00
Test014	1 - C2	Auto	285	708.00	350.26	43.19 %	1.00
Test015	1 - C3	Auto	278	731.00	482.77	55.01 %	1.00
Test016	1 - C4	Auto	287	702.00	504.08	59.22 %	1.00
Test017	1 - C5	Auto	281	601.00	432.92	56.12 %	1.00
Test018	1 - C6	Auto	294	702.00	637.89	67.35 %	1.00
Test019	1 - C7	Auto	294	599.00	438.85	57.11 %	1.00
Test020	1 - C8	Auto	277	696.00	403.27	48.89 %	1.00
Test021	1 - C9	Auto	279	620.00	426.99	53.81 %	1.00
Test022	1 - C10	Auto	264	559.00	310.60	47.95 %	1.00
Test023	1 - C11	Auto	268	603.00	181.43	26.34 %	1.00
Test024	1 - C12	Auto	262	599.00	180.88	26.50 %	1.00
Test025	1 - D1	Auto	259	598.00	155.67	22.64 %	1.00

Results Statistics for A4 - Mouse IFN-γ

Name	Plate Pos.	Clust.	Event #	MFI	SD	CV	Dilution
Test026	1 - D2	Auto	272	560.00	275.39	42.31 %	1.00
Test027	1 - D3	Auto	242	562.00	297.26	47.13 %	1.00
Test028	1 - D4	Manual	247	541.00	316.54	47.93 %	1.00
Test029	1 - D5	Manual	261	588.00	283.18	42.69 %	1.00
Test030	1 - D6	Auto	268	543.00	326.17	47.59 %	1.00
Test031	1 - D7	Auto	255	632.00	190.51	25.77 %	1.00
Test032	1 - D8	Auto	262	613.00	275.39	39.80 %	1.00
Test033	1 - D9	Auto	275	561.00	249.82	39.92 %	1.00
Test034	1 - D10	Manual	268	631.00	262.05	37.19 %	1.00
Test035	1 - D11	Auto	305	582.00	195.70	29.38 %	1.00
Test036	1 - D12	Auto	267	630.00	287.62	39.56 %	1.00
Test037	1 - E1	Auto	300	594.00	225.73	34.53 %	1.00
Test038	1 - E2	Auto	256	599.00	237.96	33.29 %	1.00
Test039	1 - E3	Auto	224	558.00	251.67	38.63 %	1.00
Test040	1 - E4	Auto	280	621.00	160.12	22.18 %	1.00
Test041	1 - E5	Auto	274	604.00	272.80	38.92 %	1.00
Test042	1 - E6	Auto	273	574.00	231.29	37.16 %	1.00
Test044	1 - E7	Auto	260	608.00	288.92	39.98 %	1.00

Quantitative Analyte Results for A4 - Mouse IFN-γ

Name	Plate Position	Nominal CC	Fitted CC	Final CC	Message
Std001	1 - A1	0.00	0.00	0.00	Fitting: Below standard range
Std002	1 - A2	10.00	10.84	10.84	
Std003	1 - A3	20.00	21.33	21.33	
Std004	1 - A4	40.00	33.04	33.04	
Std005	1 - A5	80.00	80.32	80.32	
Std006	1 - A6	156.00	171.72	171.72	
Std007	1 - A7	312.50	307.02	307.02	
Std008	1 - A8	625.00	621.69	621.69	
Std009	1 - A9	1,250.00	1,266.32	1,266.32	
Std010	1 - A10	2,500.00	2,458.61	2,458.61	
Test001	1 - B1	N/A	0.00	0.00	Fitting: Below standard range
Test002	1 - B2	N/A	0.00	0.00	Fitting: Below standard range
Test003	1 - B3	N/A	0.28	0.28	
Test004	1 - B4	N/A	0.00	0.00	Fitting: Below standard range
Test005	1 - B5	N/A	0.00	0.00	Fitting: Below standard range
Test006	1 - B6	N/A	0.00	0.00	Fitting: Below standard range
Test007	1 - B7	N/A	0.00	0.00	Fitting: Below standard range
Test008	1 - B8	N/A	0.03	0.03	
Test009	1 - B9	N/A	0.00	0.00	
Test010	1 - B10	N/A	0.21	0.21	
Test011	1 - B11	N/A	0.06	0.06	
Test012	1 - B12	N/A	0.00	0.00	Fitting: Below standard range

Quantitative Analyte Results for A4 - Mouse IFN-γ

Name	Plate Position	Nominal CC	Fitted CC	Final CC	Message
Test013	1 - C1	N/A	0.19	0.19	
Test014	1 - C2	N/A	0.01	0.01	
Test015	1 - C3	N/A	0.13	0.13	
Test016	1 - C4	N/A	0.00	0.00	
Test017	1 - C5	N/A	0.00	0.00	Fitting: Below standard range
Test018	1 - C6	N/A	0.00	0.00	
Test019	1 - C7	N/A	0.00	0.00	Fitting: Below standard range
Test020	1 - C8	N/A	0.00	0.00	Fitting: Below standard range
Test021	1 - C9	N/A	0.00	0.00	Fitting: Below standard range
Test022	1 - C10	N/A	0.00	0.00	Fitting: Below standard range
Test023	1 - C11	N/A	0.00	0.00	Fitting: Below standard range
Test024	1 - C12	N/A	0.00	0.00	Fitting: Below standard range
Test025	1 - D1	N/A	0.00	0.00	Fitting: Below standard range
Test026	1 - D2	N/A	0.00	0.00	Fitting: Below standard range
Test027	1 - D3	N/A	0.00	0.00	Fitting: Below standard range
Test028	1 - D4	N/A	0.00	0.00	Fitting: Below standard range
Test029	1 - D5	N/A	0.00	0.00	Fitting: Below standard range
Test030	1 - D6	N/A	0.00	0.00	Fitting: Below standard range
Test031	1 - D7	N/A	0.00	0.00	Fitting: Below standard range
Test032	1 - D8	N/A	0.00	0.00	Fitting: Below standard range
Test033	1 - D9	N/A	0.00	0.00	Fitting: Below standard range
Test034	1 - D10	N/A	0.00	0.00	Fitting: Below standard range
Test035	1 - D11	N/A	0.00	0.00	Fitting: Below standard range
Test036	1 - D12	N/A	0.00	0.00	Fitting: Below standard range
Test037	1 - E1	N/A	0.00	0.00	Fitting: Below standard range
Test038	1 - E2	N/A	0.00	0.00	Fitting: Below standard range
Test039	1 - E3	N/A	0.00	0.00	Fitting: Below standard range
Test040	1 - E4	N/A	0.00	0.00	Fitting: Below standard range
Test041	1 - E5	N/A	0.00	0.00	Fitting: Below standard range
Test042	1 - E6	N/A	0.00	0.00	Fitting: Below standard range
Test044	1 - E7	N/A	0.00	0.00	Fitting: Below standard range

Results Statistics for C8 - Mouse TNF

Name	Plate Pos.	Clust.	Event #	MFI	SD	CV	Dilution
Std001	1 - A1	Auto	278	413.00	472.21	81.98 %	1.00
Std002	1 - A2	Auto	295	730.00	557.46	61.22 %	1.00
Std003	1 - A3	Auto	328	926.00	557.27	53.56 %	1.00
Std004	1 - A4	Auto	365	1,356.00	293.18	20.01 %	1.00
Std005	1 - A5	Auto	295	2,514.00	550.04	20.26 %	1.00
Std006	1 - A6	Auto	296	5,011.00	888.26	17.23 %	1.00
Std007	1 - A7	Auto	296	8,679.00	1,757.62	19.05 %	1.00
Std008	1 - A8	Auto	276	16,843.00	3,277.66	17.31 %	1.00
Std009	1 - A9	Auto	290	32,425.00	6,703.76	21.08 %	1.00

Results Statistics for C8 - Mouse TNF

Name	Plate Pos.	Clust.	Event #	MFI	SD	CV	Dilution
Std010	1 - A10	Auto	306	58,065.00	11,822.07	18.46 %	1.00
Test001	1 - B1	Auto	304	441.00	523.36	81.50 %	1.00
Test002	1 - B2	Auto	311	522.00	510.76	73.72 %	1.00
Test003	1 - B3	Auto	276	600.00	517.80	69.48 %	1.00
Test004	1 - B4	Auto	324	479.00	470.35	76.62 %	1.00
Test005	1 - B5	Auto	331	476.00	298.74	53.50 %	1.00
Test006	1 - B6	Auto	286	570.00	315.79	48.93 %	1.00
Test007	1 - B7	Auto	290	419.00	392.52	69.36 %	1.00
Test008	1 - B8	Auto	311	580.00	390.67	57.37 %	1.00
Test009	1 - B9	Auto	279	558.00	272.80	48.29 %	1.00
Test010	1 - B10	Auto	315	569.00	409.94	60.97 %	1.00
Test011	1 - B11	Auto	314	521.00	455.53	71.17 %	1.00
Test012	1 - B12	Auto	319	562.00	439.59	60.10 %	1.00
Test013	1 - C1	Auto	283	685.00	364.72	46.59 %	1.00
Test014	1 - C2	Auto	305	548.00	411.79	60.19 %	1.00
Test015	1 - C3	Auto	323	553.00	508.53	70.40 %	1.00
Test016	1 - C4	Auto	296	476.00	496.86	73.71 %	1.00
Test017	1 - C5	Auto	291	473.00	528.55	79.39 %	1.00
Test018	1 - C6	Auto	286	489.00	551.34	75.96 %	1.00
Test019	1 - C7	Auto	321	479.00	458.86	75.95 %	1.00
Test020	1 - C8	Auto	284	471.00	388.81	66.06 %	1.00
Test021	1 - C9	Auto	329	518.00	458.49	68.65 %	1.00
Test022	1 - C10	Auto	288	305.00	323.21	77.41 %	1.00
Test023	1 - C11	Auto	258	416.00	184.77	38.88 %	1.00
Test024	1 - C12	Auto	257	420.00	196.82	42.00 %	1.00
Test025	1 - D1	Auto	259	417.00	141.59	31.31 %	1.00
Test026	1 - D2	Auto	264	411.00	314.31	62.16 %	1.00
Test027	1 - D3	Auto	256	390.00	249.45	57.00 %	1.00
Test028	1 - D4	Manual	252	317.00	313.01	74.72 %	1.00
Test029	1 - D5	Manual	291	359.00	311.35	66.85 %	1.00
Test030	1 - D6	Auto	261	379.00	292.07	65.61 %	1.00
Test031	1 - D7	Auto	286	417.00	210.90	42.28 %	1.00
Test032	1 - D8	Auto	280	425.00	291.15	60.22 %	1.00
Test033	1 - D9	Auto	262	342.00	267.61	63.42 %	1.00
Test034	1 - D10	Manual	279	379.00	276.50	61.78 %	1.00
Test035	1 - D11	Auto	263	448.00	214.24	40.84 %	1.00
Test036	1 - D12	Auto	298	401.00	326.17	65.66 %	1.00
Test037	1 - E1	Auto	270	368.00	236.29	54.46 %	1.00
Test038	1 - E2	Auto	276	426.00	275.95	52.09 %	1.00
Test039	1 - E3	Auto	268	325.00	306.34	65.25 %	1.00
Test040	1 - E4	Auto	281	427.00	146.41	32.37 %	1.00
Test041	1 - E5	Auto	258	361.00	258.34	60.70 %	1.00
Test042	1 - E6	Auto	263	375.00	270.57	58.79 %	1.00
Test044	1 - E7	Auto	268	357.00	292.26	64.86 %	1.00

Quantitative Analyte Results for C8 - Mouse TNF

Name	Plate Position	Nominal CC	Fitted CC	Final CC	Message
Std001	1 - A1	0.00	0.00	0.00	Fitting: Below standard range
Std002	1 - A2	10.00	11.51	11.51	
Std003	1 - A3	20.00	19.12	19.12	
Std004	1 - A4	40.00	35.91	35.91	
Std005	1 - A5	80.00	80.22	80.22	
Std006	1 - A6	156.00	172.07	172.07	
Std007	1 - A7	312.50	304.37	304.37	
Std008	1 - A8	625.00	605.89	605.89	
Std009	1 - A9	1,250.00	1,244.43	1,244.43	
Std010	1 - A10	2,500.00	2,540.28	2,540.28	
Test001	1 - B1	N/A	0.82	0.82	
Test002	1 - B2	N/A	3.70	3.70	
Test003	1 - B3	N/A	6.57	6.57	
Test004	1 - B4	N/A	2.16	2.16	
Test005	1 - B5	N/A	2.05	2.05	
Test006	1 - B6	N/A	5.45	5.45	
Test007	1 - B7	N/A	0.05	0.05	
Test008	1 - B8	N/A	5.82	5.82	
Test009	1 - B9	N/A	5.01	5.01	
Test010	1 - B10	N/A	5.42	5.42	
Test011	1 - B11	N/A	3.66	3.66	
Test012	1 - B12	N/A	5.16	5.16	
Test013	1 - C1	N/A	9.78	9.78	
Test014	1 - C2	N/A	4.64	4.64	
Test015	1 - C3	N/A	4.83	4.83	
Test016	1 - C4	N/A	2.05	2.05	
Test017	1 - C5	N/A	1.94	1.94	
Test018	1 - C6	N/A	2.51	2.51	
Test019	1 - C7	N/A	2.16	2.16	
Test020	1 - C8	N/A	1.87	1.87	
Test021	1 - C9	N/A	3.55	3.55	
Test022	1 - C10	N/A	0.00	0.00	Fitting: Below standard range and out of invertable range
Test023	1 - C11	N/A	0.00	0.00	
Test024	1 - C12	N/A	0.08	0.08	
Test025	1 - D1	N/A	0.00	0.00	
Test026	1 - D2	N/A	0.00	0.00	Fitting: Below standard range
Test027	1 - D3	N/A	0.00	0.00	Fitting: Below standard range and out of invertable range
Test028	1 - D4	N/A	0.00	0.00	Fitting: Below standard range and out of invertable range
Test029	1 - D5	N/A	0.00	0.00	Fitting: Below standard range and out of invertable range
Test030	1 - D6	N/A	0.00	0.00	Fitting: Below standard range and out of invertable range

Quantitative Analyte Results for C8 - Mouse TNF

Name	Plate Position	Nominal CC	Fitted CC	Final CC	Message
Test031	1 - D7	N/A	0.00	0.00	
Test032	1 - D8	N/A	0.26	0.26	
Test033	1 - D9	N/A	0.00	0.00	Fitting: Below standard range and out of invertable range
Test034	1 - D10	N/A	0.00	0.00	Fitting: Below standard range and out of invertable range
Test035	1 - D11	N/A	1.07	1.07	
Test036	1 - D12	N/A	0.00	0.00	Fitting: Below standard range
Test037	1 - E1	N/A	0.00	0.00	Fitting: Below standard range and out of invertable range
Test038	1 - E2	N/A	0.30	0.30	
Test039	1 - E3	N/A	0.00	0.00	Fitting: Below standard range and out of invertable range
Test040	1 - E4	N/A	0.33	0.33	
Test041	1 - E5	N/A	0.00	0.00	Fitting: Below standard range and out of invertable range
Test042	1 - E6	N/A	0.00	0.00	Fitting: Below standard range and out of invertable range
Test044	1 - E7	N/A	0.00	0.00	Fitting: Below standard range and out of invertable range

Quantitative Sample Results for Std001

Analyte Name	Event Count	Nominal CC	Fitted CC	Final CC	Message
Mouse IL-10	302	0.00	0.41	0.41	
Mouse IL-12p70	250	0.00	0.46	0.46	
Mouse IL-21	257	0.00	0.00	0.00	
Mouse IL-2	274	0.00	0.00	0.00	
Mouse IL-4	238	0.00	0.00	0.00	
Mouse IL-5	281	0.00	0.00	0.00	
Mouse IL-6	277	0.00	0.00	0.00	
Mouse IFN-γ	293	0.00	0.00	0.00	
Mouse TNF	278	0.00	0.00	0.00	

Quantitative Sample Results for Std002

Analyte Name	Event Count	Nominal CC	Fitted CC	Final CC	Message
Mouse IL-10	305	10.00	9.46	9.46	
Mouse IL-12p70	270	10.00	8.79	8.79	

Quantitative Sample Results for Std002

Analyte Name	Event Count	Nominal CC	Fitted CC	Final CC	Message
Mouse IL-21	259	10.00	12.31	12.31	
Mouse IL-2	237	10.00	11.17	11.17	
Mouse IL-4	264	10.00	11.89	11.89	
Mouse IL-5	298	10.00	11.55	11.55	
Mouse IL-6	288	10.00	10.55	10.55	
Mouse IFN-γ	287	10.00	10.84	10.84	
Mouse TNF	295	10.00	11.51	11.51	

Quantitative Sample Results for Std003

Analyte Name	Event Count	Nominal CC	Fitted CC	Final CC	Message
Mouse IL-10	321	20.00	22.39	22.39	
Mouse IL-12p70	294	20.00	23.04	23.04	
Mouse IL-21	255	20.00	21.35	21.35	
Mouse IL-2	241	20.00	20.69	20.69	
Mouse IL-4	242	20.00	20.92	20.92	
Mouse IL-5	321	20.00	20.89	20.89	
Mouse IL-6	264	20.00	21.48	21.48	
Mouse IFN-γ	313	20.00	21.33	21.33	
Mouse TNF	328	20.00	19.12	19.12	

Quantitative Sample Results for Std004

Analyte Name	Event Count	Nominal CC	Fitted CC	Final CC	Message
Mouse IL-10	710	40.00	34.84	34.84	
Mouse IL-12p70	115	40.00	36.44	36.44	
Mouse IL-21	284	40.00	30.59	30.59	
Mouse IL-2	374	40.00	33.34	33.34	
Mouse IL-4	261	40.00	32.31	32.31	
Mouse IL-5	486	40.00	33.77	33.77	
Mouse IL-6	250	40.00	32.66	32.66	
Mouse IFN-γ	408	40.00	33.04	33.04	

Quantitative Sample Results for Std004

Analyte Name	Event Count	Nominal CC	Fitted CC	Final CC	Message
Mouse TNF	365	40.00	35.91	35.91	

Quantitative Sample Results for Std005

Analyte Name	Event Count	Nominal CC	Fitted CC	Final CC	Message
Mouse IL-10	313	80.00	80.49	80.49	
Mouse IL-12p70	273	80.00	79.60	79.60	
Mouse IL-21	278	80.00	70.20	70.20	
Mouse IL-2	257	80.00	76.56	76.56	
Mouse IL-4	276	80.00	78.64	78.64	
Mouse IL-5	299	80.00	77.47	77.47	
Mouse IL-6	295	80.00	81.04	81.04	
Mouse IFN-γ	268	80.00	80.32	80.32	
Mouse TNF	295	80.00	80.22	80.22	

Quantitative Sample Results for Std006

Analyte Name	Event Count	Nominal CC	Fitted CC	Final CC	Message
Mouse IL-10	316	156.00	167.82	167.82	
Mouse IL-12p70	264	156.00	163.36	163.36	
Mouse IL-21	279	156.00	172.27	172.27	
Mouse IL-2	251	156.00	168.88	168.88	
Mouse IL-4	263	156.00	168.02	168.02	
Mouse IL-5	280	156.00	165.75	165.75	
Mouse IL-6	270	156.00	176.23	176.23	
Mouse IFN-γ	290	156.00	171.72	171.72	
Mouse TNF	296	156.00	172.07	172.07	

Quantitative Sample Results for Std007

Analyte Name	Event Count	Nominal CC	Fitted CC	Final CC	Message
Mouse IL-10	302	312.50	307.80	307.80	
Mouse IL-12p70	267	312.50	312.67	312.67	

Quantitative Sample Results for Std007

Analyte Name	Event Count	Nominal CC	Fitted CC	Final CC	Message
Mouse IL-21	289	312.50	341.58	341.58	
Mouse IL-2	280	312.50	316.65	316.65	
Mouse IL-4	283	312.50	316.23	316.23	
Mouse IL-5	306	312.50	310.60	310.60	
Mouse IL-6	259	312.50	309.34	309.34	
Mouse IFN-γ	306	312.50	307.02	307.02	
Mouse TNF	296	312.50	304.37	304.37	

Quantitative Sample Results for Std008

Analyte Name	Event Count	Nominal CC	Fitted CC	Final CC	Message
Mouse IL-10	298	625.00	615.93	615.93	
Mouse IL-12p70	288	625.00	599.71	599.71	
Mouse IL-21	269	625.00	696.82	696.82	
Mouse IL-2	280	625.00	648.06	648.06	
Mouse IL-4	256	625.00	655.85	655.85	
Mouse IL-5	416	625.00	655.61	655.61	
Mouse IL-6	259	625.00	609.80	609.80	
Mouse IFN-γ	326	625.00	621.69	621.69	
Mouse TNF	276	625.00	605.89	605.89	

Quantitative Sample Results for Std009

Analyte Name	Event Count	Nominal CC	Fitted CC	Final CC	Message
Mouse IL-10	281	1,250.00	1,230.58	1,230.58	
Mouse IL-12p70	286	1,250.00	1,279.11	1,279.11	
Mouse IL-21	310	1,250.00	1,414.41	1,414.41	
Mouse IL-2	268	1,250.00	1,363.20	1,363.20	
Mouse IL-4	231	1,250.00	1,241.86	1,241.86	
Mouse IL-5	300	1,250.00	1,324.10	1,324.10	
Mouse IL-6	247	1,250.00	1,219.54	1,219.54	
Mouse IFN-γ	294	1,250.00	1,266.32	1,266.32	

Quantitative Sample Results for Std009

Analyte Name	Event Count	Nominal CC	Fitted CC	Final CC	Message
Mouse TNF	290	1,250.00	1,244.43	1,244.43	

Quantitative Sample Results for Std010

Analyte Name	Event Count	Nominal CC	Fitted CC	Final CC	Message
Mouse IL-10	295	2,500.00	2,534.96	2,534.96	
Mouse IL-12p70	284	2,500.00	2,494.00	2,494.00	
Mouse IL-21	265	2,500.00	1,978.12	1,978.12	
Mouse IL-2	291	2,500.00	2,238.24	2,238.24	
Mouse IL-4	283	2,500.00	2,386.61	2,386.61	
Mouse IL-5	293	2,500.00	2,289.69	2,289.69	
Mouse IL-6	270	2,500.00	2,538.58	2,538.58	
Mouse IFN-γ	300	2,500.00	2,458.61	2,458.61	
Mouse TNF	306	2,500.00	2,540.28	2,540.28	

Quantitative Sample Results for Test001

Analyte Name	Event Count	Nominal CC	Fitted CC	Final CC	Message
Mouse IL-10	284	N/A	1.56	1.56	
Mouse IL-12p70	284	N/A	0.00	0.00	
Mouse IL-21	275	N/A	0.00	0.00	
Mouse IL-2	258	N/A	0.15	0.15	
Mouse IL-4	287	N/A	0.00	0.00	
Mouse IL-5	273	N/A	0.00	0.00	
Mouse IL-6	286	N/A	0.75	0.75	
Mouse IFN-γ	289	N/A	0.00	0.00	
Mouse TNF	304	N/A	0.82	0.82	

Quantitative Sample Results for Test002

Analyte Name	Event Count	Nominal CC	Fitted CC	Final CC	Message
Mouse IL-10	319	N/A	0.00	0.00	
Mouse IL-12p70	272	N/A	2.00	2.00	

Quantitative Sample Results for Test002

Analyte Name	Event Count	Nominal CC	Fitted CC	Final CC	Message
Mouse IL-21	281	N/A	0.74	0.74	
Mouse IL-2	253	N/A	0.00	0.00	
Mouse IL-4	268	N/A	0.00	0.00	
Mouse IL-5	285	N/A	0.00	0.00	
Mouse IL-6	289	N/A	0.74	0.74	
Mouse IFN-γ	263	N/A	0.00	0.00	
Mouse TNF	311	N/A	3.70	3.70	

Quantitative Sample Results for Test003

Analyte Name	Event Count	Nominal CC	Fitted CC	Final CC	Message
Mouse IL-10	306	N/A	0.96	0.96	
Mouse IL-12p70	274	N/A	0.00	0.00	
Mouse IL-21	265	N/A	1.60	1.60	
Mouse IL-2	278	N/A	0.12	0.12	
Mouse IL-4	267	N/A	0.00	0.00	
Mouse IL-5	295	N/A	0.45	0.45	
Mouse IL-6	278	N/A	2.32	2.32	
Mouse IFN-γ	303	N/A	0.28	0.28	
Mouse TNF	276	N/A	6.57	6.57	

Quantitative Sample Results for Test004

Analyte Name	Event Count	Nominal CC	Fitted CC	Final CC	Message
Mouse IL-10	279	N/A	0.00	0.00	
Mouse IL-12p70	264	N/A	0.00	0.00	
Mouse IL-21	248	N/A	0.00	0.00	
Mouse IL-2	273	N/A	0.07	0.07	
Mouse IL-4	263	N/A	0.00	0.00	
Mouse IL-5	285	N/A	0.00	0.00	
Mouse IL-6	305	N/A	1.87	1.87	
Mouse IFN-γ	311	N/A	0.00	0.00	

Quantitative Sample Results for Test004

Analyte Name	Event Count	Nominal CC	Fitted CC	Final CC	Message
Mouse TNF	324	N/A	2.16	2.16	

Quantitative Sample Results for Test005

Analyte Name	Event Count	Nominal CC	Fitted CC	Final CC	Message
Mouse IL-10	326	N/A	3.15	3.15	
Mouse IL-12p70	279	N/A	3.60	3.60	
Mouse IL-21	261	N/A	0.98	0.98	
Mouse IL-2	256	N/A	0.23	0.23	
Mouse IL-4	250	N/A	0.00	0.00	
Mouse IL-5	257	N/A	0.00	0.00	
Mouse IL-6	278	N/A	2.62	2.62	
Mouse IFN-γ	287	N/A	0.00	0.00	
Mouse TNF	331	N/A	2.05	2.05	

Quantitative Sample Results for Test006

Analyte Name	Event Count	Nominal CC	Fitted CC	Final CC	Message
Mouse IL-10	292	N/A	2.92	2.92	
Mouse IL-12p70	286	N/A	2.52	2.52	
Mouse IL-21	299	N/A	0.92	0.92	
Mouse IL-2	273	N/A	0.17	0.17	
Mouse IL-4	264	N/A	0.00	0.00	
Mouse IL-5	269	N/A	0.00	0.00	
Mouse IL-6	303	N/A	3.60	3.60	
Mouse IFN-γ	272	N/A	0.00	0.00	
Mouse TNF	286	N/A	5.45	5.45	

Quantitative Sample Results for Test007

Analyte Name	Event Count	Nominal CC	Fitted CC	Final CC	Message
Mouse IL-10	299	N/A	0.00	0.00	
Mouse IL-12p70	266	N/A	4.58	4.58	

Quantitative Sample Results for Test007

Analyte Name	Event Count	Nominal CC	Fitted CC	Final CC	Message
Mouse IL-21	258	N/A	0.64	0.64	
Mouse IL-2	268	N/A	0.00	0.00	
Mouse IL-4	256	N/A	0.00	0.00	
Mouse IL-5	299	N/A	0.00	0.00	
Mouse IL-6	321	N/A	1.65	1.65	
Mouse IFN-γ	324	N/A	0.00	0.00	
Mouse TNF	290	N/A	0.05	0.05	

Quantitative Sample Results for Test008

Analyte Name	Event Count	Nominal CC	Fitted CC	Final CC	Message
Mouse IL-10	357	N/A	0.00	0.00	
Mouse IL-12p70	295	N/A	3.07	3.07	
Mouse IL-21	275	N/A	0.64	0.64	
Mouse IL-2	236	N/A	0.00	0.00	
Mouse IL-4	257	N/A	0.00	0.00	
Mouse IL-5	302	N/A	0.00	0.00	
Mouse IL-6	271	N/A	3.30	3.30	
Mouse IFN-γ	264	N/A	0.03	0.03	
Mouse TNF	311	N/A	5.82	5.82	

Quantitative Sample Results for Test009

Analyte Name	Event Count	Nominal CC	Fitted CC	Final CC	Message
Mouse IL-10	371	N/A	1.16	1.16	
Mouse IL-12p70	264	N/A	4.23	4.23	
Mouse IL-21	186	N/A	0.93	0.93	
Mouse IL-2	291	N/A	0.06	0.06	
Mouse IL-4	247	N/A	0.00	0.00	
Mouse IL-5	288	N/A	0.00	0.00	
Mouse IL-6	318	N/A	2.30	2.30	
Mouse IFN-γ	265	N/A	0.00	0.00	

Quantitative Sample Results for Test009

Analyte Name	Event Count	Nominal CC	Fitted CC	Final CC	Message
Mouse TNF	279	N/A	5.01	5.01	

Quantitative Sample Results for Test010

Analyte Name	Event Count	Nominal CC	Fitted CC	Final CC	Message
Mouse IL-10	374	N/A	4.43	4.43	
Mouse IL-12p70	270	N/A	4.41	4.41	
Mouse IL-21	228	N/A	3.58	3.58	
Mouse IL-2	271	N/A	0.09	0.09	
Mouse IL-4	272	N/A	0.96	0.96	
Mouse IL-5	291	N/A	0.00	0.00	
Mouse IL-6	306	N/A	2.74	2.74	
Mouse IFN-γ	258	N/A	0.21	0.21	
Mouse TNF	315	N/A	5.42	5.42	

Quantitative Sample Results for Test011

Analyte Name	Event Count	Nominal CC	Fitted CC	Final CC	Message
Mouse IL-10	344	N/A	0.60	0.60	
Mouse IL-12p70	264	N/A	3.54	3.54	
Mouse IL-21	254	N/A	1.50	1.50	
Mouse IL-2	262	N/A	0.15	0.15	
Mouse IL-4	209	N/A	0.00	0.00	
Mouse IL-5	364	N/A	0.00	0.00	
Mouse IL-6	310	N/A	2.43	2.43	
Mouse IFN-γ	297	N/A	0.06	0.06	
Mouse TNF	314	N/A	3.66	3.66	

Quantitative Sample Results for Test012

Analyte Name	Event Count	Nominal CC	Fitted CC	Final CC	Message
Mouse IL-10	383	N/A	2.05	2.05	
Mouse IL-12p70	232	N/A	3.54	3.54	

Quantitative Sample Results for Test012

Analyte Name	Event Count	Nominal CC	Fitted CC	Final CC	Message
Mouse IL-21	237	N/A	1.75	1.75	
Mouse IL-2	293	N/A	0.04	0.04	
Mouse IL-4	247	N/A	0.00	0.00	
Mouse IL-5	344	N/A	0.00	0.00	
Mouse IL-6	281	N/A	2.41	2.41	
Mouse IFN-γ	286	N/A	0.00	0.00	
Mouse TNF	319	N/A	5.16	5.16	

Quantitative Sample Results for Test013

Analyte Name	Event Count	Nominal CC	Fitted CC	Final CC	Message
Mouse IL-10	343	N/A	2.37	2.37	
Mouse IL-12p70	241	N/A	1.12	1.12	
Mouse IL-21	250	N/A	1.74	1.74	
Mouse IL-2	289	N/A	0.24	0.24	
Mouse IL-4	252	N/A	0.00	0.00	
Mouse IL-5	283	N/A	0.00	0.00	
Mouse IL-6	278	N/A	4.46	4.46	
Mouse IFN-γ	285	N/A	0.19	0.19	
Mouse TNF	283	N/A	9.78	9.78	

Quantitative Sample Results for Test014

Analyte Name	Event Count	Nominal CC	Fitted CC	Final CC	Message
Mouse IL-10	333	N/A	0.00	0.00	
Mouse IL-12p70	274	N/A	4.69	4.69	
Mouse IL-21	234	N/A	1.22	1.22	
Mouse IL-2	272	N/A	0.02	0.02	
Mouse IL-4	227	N/A	0.00	0.00	
Mouse IL-5	320	N/A	0.00	0.00	
Mouse IL-6	294	N/A	2.52	2.52	
Mouse IFN-γ	285	N/A	0.01	0.01	

Quantitative Sample Results for Test014

Analyte Name	Event Count	Nominal CC	Fitted CC	Final CC	Message
Mouse TNF	305	N/A	4.64	4.64	

Quantitative Sample Results for Test015

Analyte Name	Event Count	Nominal CC	Fitted CC	Final CC	Message
Mouse IL-10	344	N/A	5.34	5.34	
Mouse IL-12p70	268	N/A	1.59	1.59	
Mouse IL-21	265	N/A	2.70	2.70	
Mouse IL-2	273	N/A	0.00	0.00	
Mouse IL-4	227	N/A	0.00	0.00	
Mouse IL-5	299	N/A	0.00	0.00	
Mouse IL-6	283	N/A	1.67	1.67	
Mouse IFN-γ	278	N/A	0.13	0.13	
Mouse TNF	323	N/A	4.83	4.83	

Quantitative Sample Results for Test016

Analyte Name	Event Count	Nominal CC	Fitted CC	Final CC	Message
Mouse IL-10	318	N/A	0.96	0.96	
Mouse IL-12p70	281	N/A	3.18	3.18	
Mouse IL-21	253	N/A	1.11	1.11	
Mouse IL-2	288	N/A	0.00	0.00	
Mouse IL-4	248	N/A	0.00	0.00	
Mouse IL-5	288	N/A	0.00	0.00	
Mouse IL-6	311	N/A	1.82	1.82	
Mouse IFN-γ	287	N/A	0.00	0.00	
Mouse TNF	296	N/A	2.05	2.05	

Quantitative Sample Results for Test017

Analyte Name	Event Count	Nominal CC	Fitted CC	Final CC	Message
Mouse IL-10	355	N/A	0.00	0.00	
Mouse IL-12p70	233	N/A	0.00	0.00	

Quantitative Sample Results for Test017

Analyte Name	Event Count	Nominal CC	Fitted CC	Final CC	Message
Mouse IL-21	287	N/A	1.82	1.82	
Mouse IL-2	271	N/A	0.00	0.00	
Mouse IL-4	277	N/A	0.00	0.00	
Mouse IL-5	297	N/A	0.00	0.00	
Mouse IL-6	271	N/A	2.02	2.02	
Mouse IFN-γ	281	N/A	0.00	0.00	
Mouse TNF	291	N/A	1.94	1.94	

Quantitative Sample Results for Test018

Analyte Name	Event Count	Nominal CC	Fitted CC	Final CC	Message
Mouse IL-10	374	N/A	2.37	2.37	
Mouse IL-12p70	225	N/A	0.00	0.00	
Mouse IL-21	204	N/A	0.33	0.33	
Mouse IL-2	250	N/A	0.00	0.00	
Mouse IL-4	270	N/A	0.00	0.00	
Mouse IL-5	274	N/A	0.00	0.00	
Mouse IL-6	307	N/A	1.71	1.71	
Mouse IFN-γ	294	N/A	0.00	0.00	
Mouse TNF	286	N/A	2.51	2.51	

Quantitative Sample Results for Test019

Analyte Name	Event Count	Nominal CC	Fitted CC	Final CC	Message
Mouse IL-10	311	N/A	0.00	0.00	
Mouse IL-12p70	253	N/A	0.94	0.94	
Mouse IL-21	264	N/A	1.30	1.30	
Mouse IL-2	267	N/A	0.08	0.08	
Mouse IL-4	234	N/A	0.78	0.78	
Mouse IL-5	307	N/A	0.00	0.00	
Mouse IL-6	309	N/A	2.03	2.03	
Mouse IFN-γ	294	N/A	0.00	0.00	

Quantitative Sample Results for Test019

Analyte Name	Event Count	Nominal CC	Fitted CC	Final CC	Message
Mouse TNF	321	N/A	2.16	2.16	

Quantitative Sample Results for Test020

Analyte Name	Event Count	Nominal CC	Fitted CC	Final CC	Message
Mouse IL-10	364	N/A	3.94	3.94	
Mouse IL-12p70	262	N/A	0.00	0.00	
Mouse IL-21	279	N/A	0.85	0.85	
Mouse IL-2	273	N/A	0.07	0.07	
Mouse IL-4	272	N/A	0.00	0.00	
Mouse IL-5	293	N/A	0.00	0.00	
Mouse IL-6	283	N/A	2.15	2.15	
Mouse IFN-γ	277	N/A	0.00	0.00	
Mouse TNF	284	N/A	1.87	1.87	

Quantitative Sample Results for Test021

Analyte Name	Event Count	Nominal CC	Fitted CC	Final CC	Message
Mouse IL-10	376	N/A	0.96	0.96	
Mouse IL-12p70	220	N/A	0.00	0.00	
Mouse IL-21	268	N/A	0.87	0.87	
Mouse IL-2	255	N/A	0.00	0.00	
Mouse IL-4	255	N/A	0.00	0.00	
Mouse IL-5	293	N/A	0.00	0.00	
Mouse IL-6	291	N/A	1.73	1.73	
Mouse IFN-γ	279	N/A	0.00	0.00	
Mouse TNF	329	N/A	3.55	3.55	

Quantitative Sample Results for Test022

Analyte Name	Event Count	Nominal CC	Fitted CC	Final CC	Message
Mouse IL-10	261	N/A	0.00	0.00	
Mouse IL-12p70	223	N/A	0.00	0.00	

Quantitative Sample Results for Test022

Analyte Name	Event Count	Nominal CC	Fitted CC	Final CC	Message
Mouse IL-21	265	N/A	0.31	0.31	
Mouse IL-2	232	N/A	0.00	0.00	
Mouse IL-4	295	N/A	0.00	0.00	
Mouse IL-5	249	N/A	0.00	0.00	
Mouse IL-6	318	N/A	0.44	0.44	
Mouse IFN-γ	264	N/A	0.00	0.00	
Mouse TNF	288	N/A	0.00	0.00	

Quantitative Sample Results for Test023

Analyte Name	Event Count	Nominal CC	Fitted CC	Final CC	Message
Mouse IL-10	291	N/A	0.00	0.00	
Mouse IL-12p70	210	N/A	0.00	0.00	
Mouse IL-21	263	N/A	0.28	0.28	
Mouse IL-2	273	N/A	0.00	0.00	
Mouse IL-4	304	N/A	0.00	0.00	
Mouse IL-5	290	N/A	0.00	0.00	
Mouse IL-6	296	N/A	0.78	0.78	
Mouse IFN-γ	268	N/A	0.00	0.00	
Mouse TNF	258	N/A	0.00	0.00	

Quantitative Sample Results for Test024

Analyte Name	Event Count	Nominal CC	Fitted CC	Final CC	Message
Mouse IL-10	282	N/A	0.00	0.00	
Mouse IL-12p70	249	N/A	0.00	0.00	
Mouse IL-21	253	N/A	0.00	0.00	
Mouse IL-2	259	N/A	0.00	0.00	
Mouse IL-4	291	N/A	0.00	0.00	
Mouse IL-5	279	N/A	0.00	0.00	
Mouse IL-6	300	N/A	0.82	0.82	
Mouse IFN-γ	262	N/A	0.00	0.00	

Quantitative Sample Results for Test024

Analyte Name	Event Count	Nominal CC	Fitted CC	Final CC	Message
Mouse TNF	257	N/A	0.08	0.08	

Quantitative Sample Results for Test025

Analyte Name	Event Count	Nominal CC	Fitted CC	Final CC	Message
Mouse IL-10	274	N/A	0.00	0.00	
Mouse IL-12p70	213	N/A	2.58	2.58	
Mouse IL-21	256	N/A	0.00	0.00	
Mouse IL-2	247	N/A	0.00	0.00	
Mouse IL-4	319	N/A	0.00	0.00	
Mouse IL-5	277	N/A	0.00	0.00	
Mouse IL-6	286	N/A	0.77	0.77	
Mouse IFN-γ	259	N/A	0.00	0.00	
Mouse TNF	259	N/A	0.00	0.00	

Quantitative Sample Results for Test026

Analyte Name	Event Count	Nominal CC	Fitted CC	Final CC	Message
Mouse IL-10	275	N/A	0.00	0.00	
Mouse IL-12p70	235	N/A	0.00	0.00	
Mouse IL-21	248	N/A	0.00	0.00	
Mouse IL-2	265	N/A	0.00	0.00	
Mouse IL-4	283	N/A	0.00	0.00	
Mouse IL-5	271	N/A	0.00	0.00	
Mouse IL-6	306	N/A	0.36	0.36	
Mouse IFN-γ	272	N/A	0.00	0.00	
Mouse TNF	264	N/A	0.00	0.00	

Quantitative Sample Results for Test027

Analyte Name	Event Count	Nominal CC	Fitted CC	Final CC	Message
Mouse IL-10	263	N/A	0.00	0.00	
Mouse IL-12p70	217	N/A	0.00	0.00	

Quantitative Sample Results for Test027

Analyte Name	Event Count	Nominal CC	Fitted CC	Final CC	Message
Mouse IL-21	276	N/A	0.00	0.00	
Mouse IL-2	265	N/A	0.00	0.00	
Mouse IL-4	298	N/A	0.00	0.00	
Mouse IL-5	320	N/A	0.00	0.00	
Mouse IL-6	279	N/A	0.91	0.91	
Mouse IFN-γ	242	N/A	0.00	0.00	
Mouse TNF	256	N/A	0.00	0.00	

Quantitative Sample Results for Test028

Analyte Name	Event Count	Nominal CC	Fitted CC	Final CC	Message
Mouse IL-10	311	N/A	0.00	0.00	
Mouse IL-12p70	250	N/A	0.00	0.00	
Mouse IL-21	233	N/A	0.19	0.19	
Mouse IL-2	228	N/A	0.00	0.00	
Mouse IL-4	332	N/A	0.00	0.00	
Mouse IL-5	273	N/A	0.00	0.00	
Mouse IL-6	325	N/A	0.14	0.14	
Mouse IFN-γ	247	N/A	0.00	0.00	
Mouse TNF	252	N/A	0.00	0.00	

Quantitative Sample Results for Test029

Analyte Name	Event Count	Nominal CC	Fitted CC	Final CC	Message
Mouse IL-10	270	N/A	0.00	0.00	
Mouse IL-12p70	252	N/A	0.00	0.00	
Mouse IL-21	276	N/A	0.14	0.14	
Mouse IL-2	240	N/A	0.00	0.00	
Mouse IL-4	300	N/A	0.00	0.00	
Mouse IL-5	272	N/A	0.00	0.00	
Mouse IL-6	297	N/A	0.29	0.29	
Mouse IFN-γ	261	N/A	0.00	0.00	

Quantitative Sample Results for Test029

Analyte Name	Event Count	Nominal CC	Fitted CC	Final CC	Message
Mouse TNF	291	N/A	0.00	0.00	

Quantitative Sample Results for Test030

Analyte Name	Event Count	Nominal CC	Fitted CC	Final CC	Message
Mouse IL-10	253	N/A	0.00	0.00	
Mouse IL-12p70	248	N/A	0.00	0.00	
Mouse IL-21	297	N/A	0.00	0.00	
Mouse IL-2	251	N/A	0.00	0.00	
Mouse IL-4	324	N/A	0.00	0.00	
Mouse IL-5	262	N/A	0.00	0.00	
Mouse IL-6	287	N/A	1.09	1.09	
Mouse IFN-γ	268	N/A	0.00	0.00	
Mouse TNF	261	N/A	0.00	0.00	

Quantitative Sample Results for Test031

Analyte Name	Event Count	Nominal CC	Fitted CC	Final CC	Message
Mouse IL-10	295	N/A	0.00	0.00	
Mouse IL-12p70	241	N/A	0.00	0.00	
Mouse IL-21	245	N/A	0.00	0.00	
Mouse IL-2	238	N/A	0.00	0.00	
Mouse IL-4	296	N/A	0.00	0.00	
Mouse IL-5	274	N/A	0.00	0.00	
Mouse IL-6	319	N/A	1.39	1.39	
Mouse IFN-γ	255	N/A	0.00	0.00	
Mouse TNF	286	N/A	0.00	0.00	

Quantitative Sample Results for Test032

Analyte Name	Event Count	Nominal CC	Fitted CC	Final CC	Message
Mouse IL-10	282	N/A	0.00	0.00	
Mouse IL-12p70	234	N/A	0.00	0.00	

Quantitative Sample Results for Test032

Analyte Name	Event Count	Nominal CC	Fitted CC	Final CC	Message
Mouse IL-21	300	N/A	0.24	0.24	
Mouse IL-2	291	N/A	0.00	0.00	
Mouse IL-4	266	N/A	0.00	0.00	
Mouse IL-5	275	N/A	0.00	0.00	
Mouse IL-6	281	N/A	1.03	1.03	
Mouse IFN-γ	262	N/A	0.00	0.00	
Mouse TNF	280	N/A	0.26	0.26	

Quantitative Sample Results for Test033

Analyte Name	Event Count	Nominal CC	Fitted CC	Final CC	Message
Mouse IL-10	271	N/A	0.00	0.00	
Mouse IL-12p70	283	N/A	0.00	0.00	
Mouse IL-21	236	N/A	0.00	0.00	
Mouse IL-2	250	N/A	0.00	0.00	
Mouse IL-4	301	N/A	0.00	0.00	
Mouse IL-5	287	N/A	0.00	0.00	
Mouse IL-6	286	N/A	0.62	0.62	
Mouse IFN-γ	275	N/A	0.00	0.00	
Mouse TNF	262	N/A	0.00	0.00	

Quantitative Sample Results for Test034

Analyte Name	Event Count	Nominal CC	Fitted CC	Final CC	Message
Mouse IL-10	279	N/A	0.00	0.00	
Mouse IL-12p70	281	N/A	0.00	0.00	
Mouse IL-21	259	N/A	0.24	0.24	
Mouse IL-2	241	N/A	0.00	0.00	
Mouse IL-4	322	N/A	0.00	0.00	
Mouse IL-5	241	N/A	0.00	0.00	
Mouse IL-6	268	N/A	0.33	0.33	
Mouse IFN-γ	268	N/A	0.00	0.00	

Quantitative Sample Results for Test034

Analyte Name	Event Count	Nominal CC	Fitted CC	Final CC	Message
Mouse TNF	279	N/A	0.00	0.00	

Quantitative Sample Results for Test035

Analyte Name	Event Count	Nominal CC	Fitted CC	Final CC	Message
Mouse IL-10	308	N/A	0.00	0.00	
Mouse IL-12p70	175	N/A	0.00	0.00	
Mouse IL-21	266	N/A	0.00	0.00	
Mouse IL-2	225	N/A	0.00	0.00	
Mouse IL-4	291	N/A	0.00	0.00	
Mouse IL-5	294	N/A	0.00	0.00	
Mouse IL-6	317	N/A	1.34	1.34	
Mouse IFN-γ	305	N/A	0.00	0.00	
Mouse TNF	263	N/A	1.07	1.07	

Quantitative Sample Results for Test036

Analyte Name	Event Count	Nominal CC	Fitted CC	Final CC	Message
Mouse IL-10	253	N/A	0.00	0.00	
Mouse IL-12p70	223	N/A	0.00	0.00	
Mouse IL-21	258	N/A	0.00	0.00	
Mouse IL-2	269	N/A	0.00	0.00	
Mouse IL-4	313	N/A	0.00	0.00	
Mouse IL-5	394	N/A	0.00	0.00	
Mouse IL-6	306	N/A	0.78	0.78	
Mouse IFN-γ	267	N/A	0.00	0.00	
Mouse TNF	298	N/A	0.00	0.00	

Quantitative Sample Results for Test037

Analyte Name	Event Count	Nominal CC	Fitted CC	Final CC	Message
Mouse IL-10	296	N/A	0.00	0.00	
Mouse IL-12p70	195	N/A	0.00	0.00	

Quantitative Sample Results for Test037

Analyte Name	Event Count	Nominal CC	Fitted CC	Final CC	Message
Mouse IL-21	275	N/A	0.00	0.00	
Mouse IL-2	253	N/A	0.00	0.00	
Mouse IL-4	276	N/A	0.00	0.00	
Mouse IL-5	286	N/A	0.00	0.00	
Mouse IL-6	286	N/A	0.35	0.35	
Mouse IFN-γ	300	N/A	0.00	0.00	
Mouse TNF	270	N/A	0.00	0.00	

Quantitative Sample Results for Test038

Analyte Name	Event Count	Nominal CC	Fitted CC	Final CC	Message
Mouse IL-10	262	N/A	0.00	0.00	
Mouse IL-12p70	228	N/A	0.00	0.00	
Mouse IL-21	287	N/A	0.00	0.00	
Mouse IL-2	256	N/A	0.00	0.00	
Mouse IL-4	303	N/A	0.00	0.00	
Mouse IL-5	280	N/A	0.00	0.00	
Mouse IL-6	287	N/A	0.53	0.53	
Mouse IFN-γ	256	N/A	0.00	0.00	
Mouse TNF	276	N/A	0.30	0.30	

Quantitative Sample Results for Test039

Analyte Name	Event Count	Nominal CC	Fitted CC	Final CC	Message
Mouse IL-10	264	N/A	0.00	0.00	
Mouse IL-12p70	236	N/A	0.00	0.00	
Mouse IL-21	278	N/A	0.00	0.00	
Mouse IL-2	271	N/A	0.00	0.00	
Mouse IL-4	302	N/A	0.00	0.00	
Mouse IL-5	286	N/A	0.00	0.00	
Mouse IL-6	276	N/A	0.76	0.76	
Mouse IFN-γ	224	N/A	0.00	0.00	

Quantitative Sample Results for Test039

Analyte Name	Event Count	Nominal CC	Fitted CC	Final CC	Message
Mouse TNF	268	N/A	0.00	0.00	

Quantitative Sample Results for Test040

Analyte Name	Event Count	Nominal CC	Fitted CC	Final CC	Message
Mouse IL-10	285	N/A	0.00	0.00	
Mouse IL-12p70	217	N/A	0.00	0.00	
Mouse IL-21	239	N/A	0.08	0.08	
Mouse IL-2	286	N/A	0.00	0.00	
Mouse IL-4	266	N/A	0.00	0.00	
Mouse IL-5	280	N/A	0.00	0.00	
Mouse IL-6	326	N/A	1.32	1.32	
Mouse IFN-γ	280	N/A	0.00	0.00	
Mouse TNF	281	N/A	0.33	0.33	

Quantitative Sample Results for Test041

Analyte Name	Event Count	Nominal CC	Fitted CC	Final CC	Message
Mouse IL-10	299	N/A	0.00	0.00	
Mouse IL-12p70	244	N/A	0.00	0.00	
Mouse IL-21	266	N/A	0.00	0.00	
Mouse IL-2	257	N/A	0.00	0.00	
Mouse IL-4	285	N/A	0.00	0.00	
Mouse IL-5	270	N/A	0.00	0.00	
Mouse IL-6	304	N/A	0.87	0.87	
Mouse IFN-γ	274	N/A	0.00	0.00	
Mouse TNF	258	N/A	0.00	0.00	

Quantitative Sample Results for Test042

Analyte Name	Event Count	Nominal CC	Fitted CC	Final CC	Message
Mouse IL-10	267	N/A	0.00	0.00	
Mouse IL-12p70	245	N/A	0.00	0.00	

Quantitative Sample Results for Test042

Analyte Name	Event Count	Nominal CC	Fitted CC	Final CC	Message
Mouse IL-21	250	N/A	0.00	0.00	
Mouse IL-2	256	N/A	0.00	0.00	
Mouse IL-4	329	N/A	0.00	0.00	
Mouse IL-5	245	N/A	0.00	0.00	
Mouse IL-6	282	N/A	0.62	0.62	
Mouse IFN-γ	273	N/A	0.00	0.00	
Mouse TNF	263	N/A	0.00	0.00	

Quantitative Sample Results for Test044

Analyte Name	Event Count	Nominal CC	Fitted CC	Final CC	Message
Mouse IL-10	299	N/A	0.00	0.00	
Mouse IL-12p70	237	N/A	0.00	0.00	
Mouse IL-21	270	N/A	0.00	0.00	
Mouse IL-2	266	N/A	0.00	0.00	
Mouse IL-4	300	N/A	0.00	0.00	
Mouse IL-5	298	N/A	0.00	0.00	
Mouse IL-6	276	N/A	0.25	0.25	
Mouse IFN-γ	260	N/A	0.00	0.00	
Mouse TNF	268	N/A	0.00	0.00	

Sample File Assignment

Sample Name	File Name
Std001	B01 0 pg_mL.fcs
Std002	B02 1_256.fcs
Std003	B03 1_128.fcs
Std004	B04 1_64.fcs
Std005	B05 1_32.fcs
Std006	B06 1_16.fcs
Std007	B07 1_8.fcs
Std008	B08 1_4.fcs
Std009	B09 1_2.fcs
Std010	B10 Top Standard.fcs
Test001	C01 Untreated_1.fcs
Test002	C02 68A_24h.fcs
Test003	C03 68V_24h.fcs
Test004	C04 139A_24h.fcs
Test005	C05 164D_24h.fcs
Test006	C06 224D_24h.fcs
Test007	D01 Untreated_2.fcs
Test008	E01 Untreated_3.fcs
Test009	D02 68A_48h.fcs
Test010	D03 68V_48h.fcs
Test011	D04 139A_48h.fcs
Test012	D05 164D_48h.fcs
Test013	D06 224D_48h.fcs
Test014	E02 wild type_24h_1.fcs
Test015	E03 G12A_24h_1.fcs
Test016	E04 G12V_24h_1.fcs
Test017	E05 G13D_24h_1.fcs
Test018	F02 wild type_48h_1.fcs
Test019	F03 G12A_48h_1.fcs
Test020	F04 G12V_48h_1.fcs
Test021	F05 G13D_48h_1.fcs
Test022	H01 WildType_24H_2.fcs
Test023	H02 G12A_24H_2.fcs
Test024	H03 G12V_24H_2.fcs
Test025	H04 G13D_24H_2.fcs
Test026	H05 WildType_48H_2.fcs
Test027	H06 G12A_48H_2.fcs
Test028	H07 G12V_48H_2.fcs
Test029	H08 G13D_48H_2.fcs
Test030	G01 Untreated_0h.fcs
Test031	G02 Untreated_24h.fcs
Test032	G03 Untreated_48h.fcs
Test033	D08 Untreated_0h.fcs
Test034	D09 Untreated_24h.fcs

Sample File Assignment

Sample Name	File Name
Test035	D10 Untreated_48h.fcs
Test036	E08 WildType_24H_3.fcs
Test037	E09 G12A_24H_3.fcs
Test038	E10 G12V_24H_3.fcs
Test039	E11 G13D_24H_3.fcs
Test040	F08 WildType_48H_3.fcs
Test041	F09 G12A_48H_3.fcs
Test042	F10 G12V_48H_3.fcs
Test044	F11 G13D_48H_3.fcs
