# Supplementary material for: In silico-guided sequence modifications of K-ras epitopes improve immunological outcome against G12V and G13D mutant KRAS antigens
Source: PeerJ. 2018 Jul 20;6:e5056. doi: 10.7717/peerj.5056 (PMC6055689; doi:10.7717/peerj.5056)
Supplement: Supplemental Information 13 — A file generated by BD Bioscience reporting mouse IL-2, IL-4, IL-5, IL-6, IL-10, IL12p70, IL-21, TNF-alpha and IFN-gamma components on PBMC samples treated with synthetic peptide mimotopes 68-V, 164-D and 224-D. PBMC sampled treated with synthetic peptide mimotopes wild-type KRAS, G12V and G13D were included as controls. Untreated PBMC samples and standard curve of all cytokine components were also included. The final single replicate data are reported in number of events, median fluorescence intensity (MFI), nominal concentration (pg/mL), fitted concentration (%), and percentage of recovery (%). [file peerj-06-5056-s013.pdf]

**Plex Name:**	New Plex
**Created by:**	Administrator
**Creation Date:**	6/15/2017
**Intrument:**	BD Accuri C6 Plus

Instrument

Instrument Name:	BD Accuri C6 Plus
Scatter Parameter:	FSC-A
Number of Scatter Peaks:	1
Clustering Parameter(s):	FL4-A, FL3-A
Reporter Parameter(s):	PE-A

Debris Filter is active!

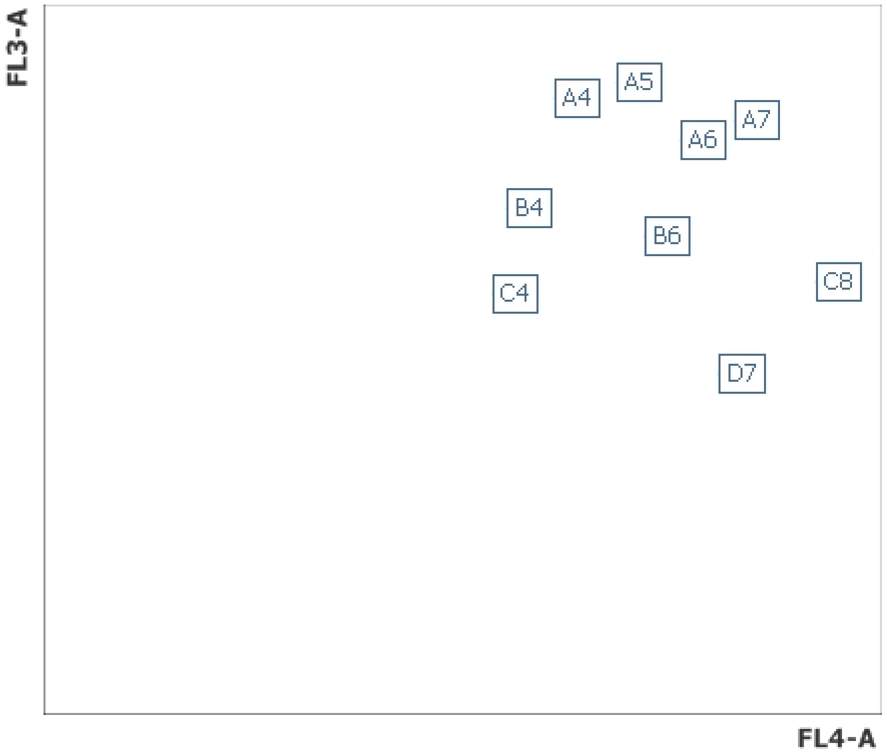

Layout 1

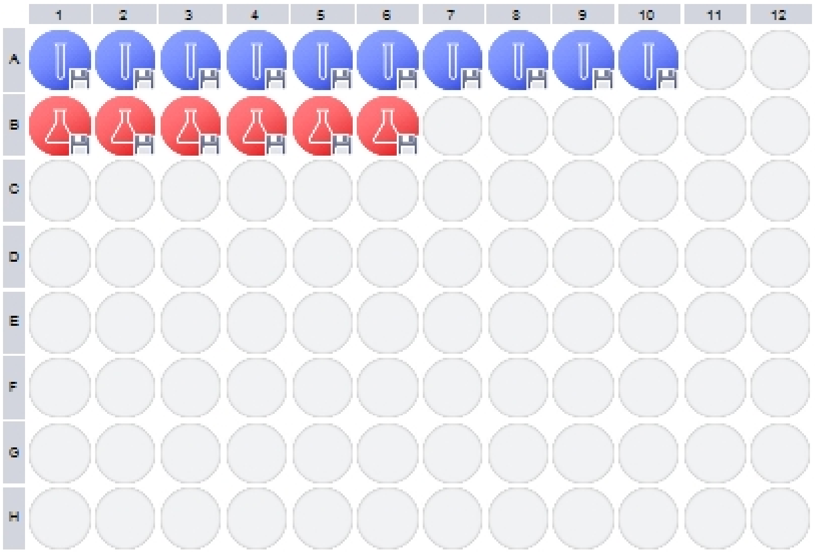

Plex Components

		Analyte		
Name	Lot Number	Name	Model	2nd Reporter
C4		Mouse IL-10	Quantitative	No
D7		Mouse IL-12p70	Quantitative	No
B6		Mouse IL-21	Quantitative	No
A5		Mouse IL-2	Quantitative	No
A7		Mouse IL-4	Quantitative	No
A6		Mouse IL-5	Quantitative	No
B4		Mouse IL-6	Quantitative	No
A4		Mouse IFN-γ	Quantitative	No
C8		Mouse TNF	Quantitative	No

Standard Samples of Quantitative Analysis

Reporter Parameter 1	
Sample Name	Concentration
Std001	0.00 pg/mL
Std002	10.00 pg/mL

Standard Samples of Quantitative Analysis

Reporter Parameter 1	
Sample Name	Concentration
Std003	20.00 pg/mL
Std005	40.00 pg/mL
Std004	80.00 pg/mL
Std006	156.00 pg/mL
Std007	312.50 pg/mL
Std008	625.00 pg/mL
Std009	1,250.00 pg/mL
Std010	2,500.00 pg/mL

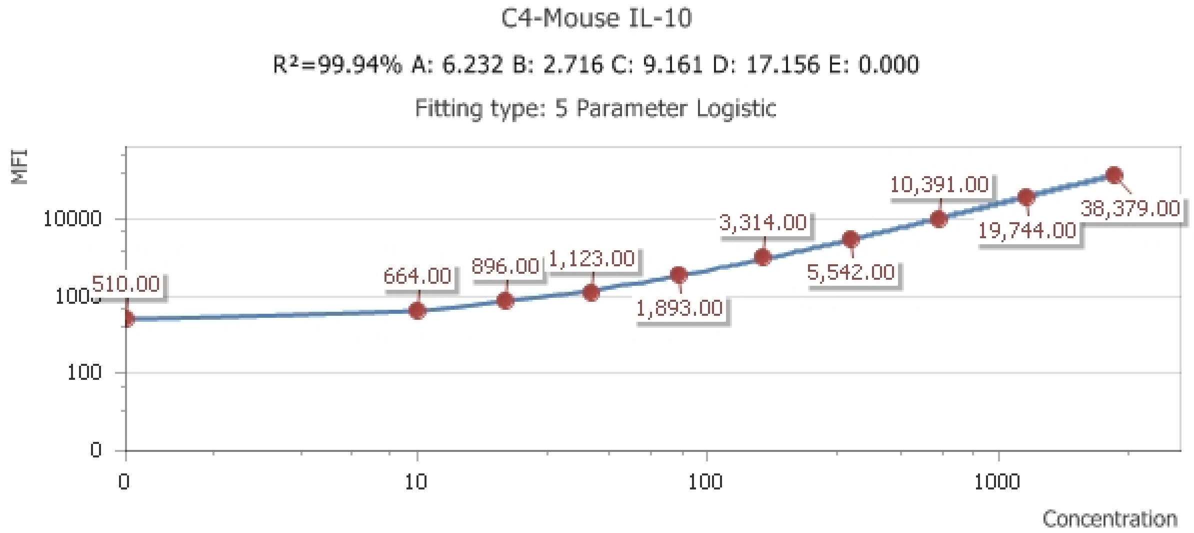

Name	Event #	MFI	SD	CV% (MFI)	Nominal CC pg/mL	Fitted CC pg/mL	Recovery %
Std001	303	510.00	495.93	72.94 %	0.00	0.46	0.00 %
Std002	305	664.00	542.26	62.58 %	10.00	9.52	95.24 %
Std003	323	896.00	570.06	56.32 %	20.00	22.11	110.57 %
Std005	710	1,123.00	255.19	21.43 %	40.00	34.98	87.45 %
Std004	313	1,893.00	432.18	23.57 %	80.00	80.68	100.85 %
Std006	316	3,314.00	686.63	20.41 %	156.00	167.99	107.69 %
Std007	299	5,542.00	863.61	14.36 %	312.50	307.30	98.34 %
Std008	298	10,391.00	1,984.27	17.47 %	625.00	615.72	98.51 %
Std009	281	19,744.00	3,660.17	15.77 %	1,250.00	1,230.20	98.42 %
Std010	295	38,379.00	7,883.73	17.81 %	2,500.00	2,536.37	101.45 %

D7-Mouse IL-12p70

R^2=99.94% A: 5.929 B: 2.529 C: 9.459 D: 17.534 E: 0.000

Fitting type: 5 Parameter Logistic

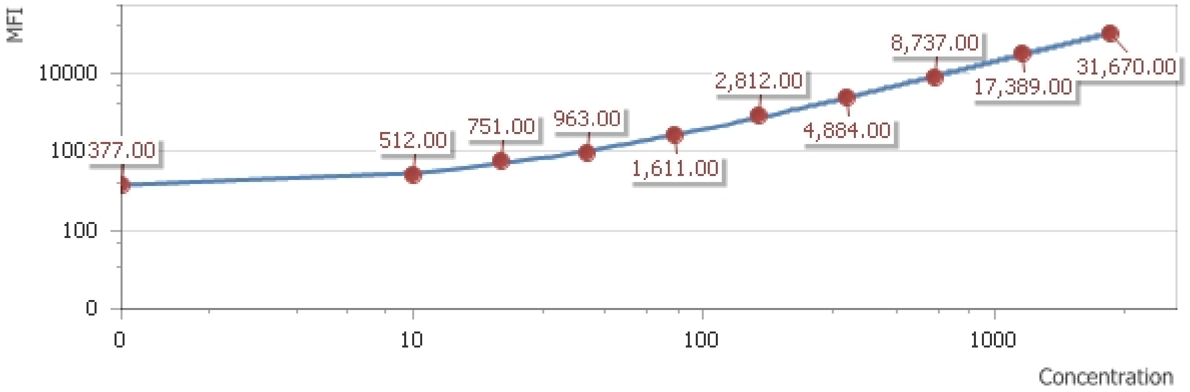

Name	Event #	MFI	SD	CV% (MFI)	Nominal CC pg/mL	Fitted CC pg/mL	Recovery %
Std001	250	377.00	467.39	80.22 %	0.00	0.46	0.00 %
Std002	270	512.00	577.29	75.20 %	10.00	8.79	87.89 %
Std003	294	751.00	527.62	59.01 %	20.00	23.04	115.22 %
Std005	115	963.00	252.04	24.78 %	40.00	36.44	91.10 %
Std004	273	1,611.00	393.26	21.88 %	80.00	79.60	99.51 %
Std006	264	2,812.00	497.41	17.94 %	156.00	163.36	104.72 %
Std007	267	4,884.00	843.60	13.51 %	312.50	312.67	100.05 %
Std008	288	8,737.00	1,159.39	11.78 %	625.00	599.71	95.95 %
Std009	286	17,389.00	3,275.06	14.15 %	1,250.00	1,279.11	102.33 %
Std010	284	31,670.00	5,326.24	13.63 %	2,500.00	2,494.00	99.76 %

B6-Mouse IL-21

R^2=99.51% A: 5.982 B: 2.093 C: 6.806 D: 14.920 E: -0.209

Fitting type: 5 Parameter Logistic

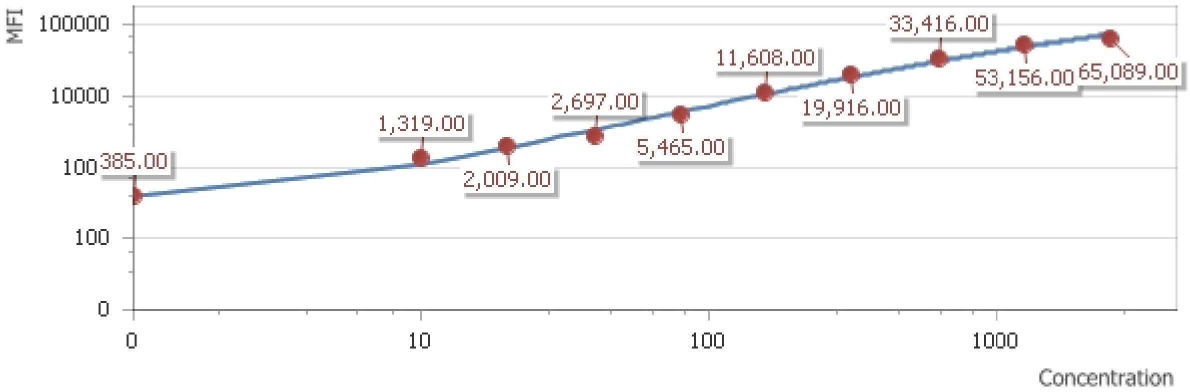

Name	Event #	MFI	SD	CV% (MFI)	Nominal CC pg/mL	Fitted CC pg/mL	Recovery %
Std001	257	385.00	433.66	80.82 %	0.00	0.00	0.00 %
Std002	259	1,319.00	565.61	37.15 %	10.00	12.31	123.05 %
Std003	255	2,009.00	624.17	27.46 %	20.00	21.35	106.73 %
Std005	284	2,697.00	514.28	18.82 %	40.00	30.59	76.47 %
Std004	278	5,465.00	1,175.33	21.70 %	80.00	70.20	87.75 %
Std006	279	11,608.00	2,268.38	19.23 %	156.00	172.27	110.43 %
Std007	290	19,916.00	4,451.14	20.01 %	312.50	341.58	109.31 %
Std008	269	33,416.00	6,753.24	19.99 %	625.00	696.82	111.49 %
Std009	310	53,156.00	8,737.89	16.15 %	1,250.00	1,414.41	113.15 %
Std010	265	65,089.00	10,310.74	16.28 %	2,500.00	1,978.12	79.12 %

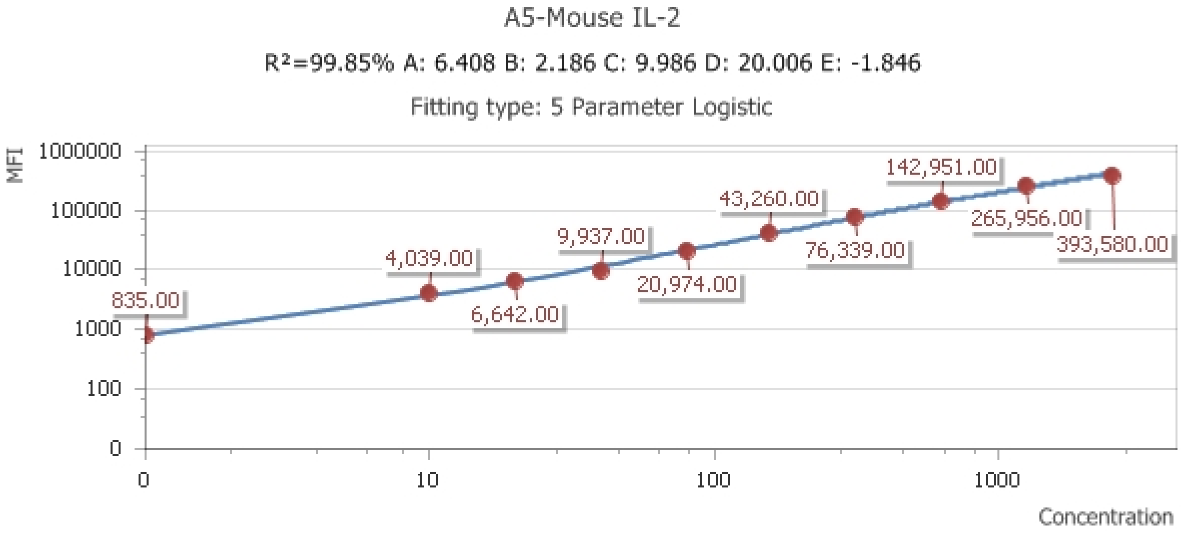

Name	Event #	MFI	SD	CV% (MFI)	Nominal CC pg/mL	Fitted CC pg/mL	Recovery %
Std001	274	835.00	524.10	47.41 %	0.00	0.00	0.00 %
Std002	237	4,039.00	795.04	16.81 %	10.00	11.14	111.36 %
Std003	242	6,642.00	1,327.67	17.74 %	20.00	20.79	103.97 %
Std005	374	9,937.00	1,805.99	15.94 %	40.00	33.28	83.20 %
Std004	257	20,974.00	4,224.67	18.03 %	80.00	76.49	95.61 %
Std006	251	43,260.00	7,249.91	16.80 %	156.00	168.84	108.23 %
Std007	280	76,339.00	16,047.85	17.93 %	312.50	316.69	101.34 %
Std008	280	142,951.00	27,722.77	17.67 %	625.00	648.22	103.72 %
Std009	271	265,956.00	44,028.03	15.27 %	1,250.00	1,364.41	109.15 %
Std010	291	393,580.00	57,123.84	12.43 %	2,500.00	2,237.01	89.48 %

A7-Mouse IL-4

R^2=99.82% A: 6.570 B: 2.333 C: 7.448 D: 17.955 E: -0.153

Fitting type: 5 Parameter Logistic

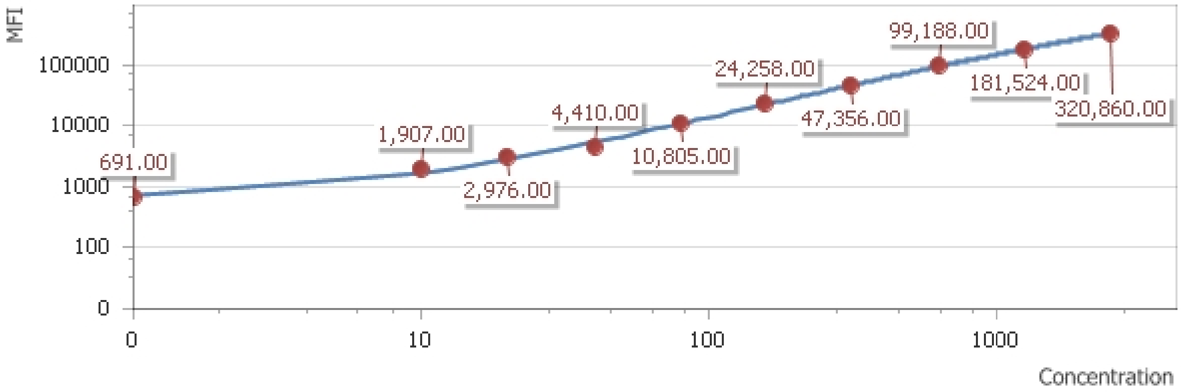

Name	Event #	MFI	SD	CV% (MFI)	Nominal CC pg/mL	Fitted CC pg/mL	Recovery %
Std001	238	691.00	505.01	60.65 %	0.00	0.00	0.00 %
Std002	266	1,907.00	550.79	27.81 %	10.00	11.89	118.93 %
Std003	242	2,976.00	880.48	26.49 %	20.00	20.92	104.58 %
Std005	262	4,410.00	961.65	20.79 %	40.00	32.31	80.78 %
Std004	276	10,805.00	2,680.17	24.43 %	80.00	78.64	98.30 %
Std006	263	24,258.00	5,094.95	20.44 %	156.00	168.02	107.71 %
Std007	284	47,356.00	12,477.38	26.84 %	312.50	316.23	101.19 %
Std008	258	99,188.00	24,156.56	23.96 %	625.00	655.85	104.94 %
Std009	231	181,524.00	40,974.62	20.29 %	1,250.00	1,241.86	99.35 %
Std010	283	320,860.00	59,687.99	17.34 %	2,500.00	2,386.61	95.46 %

A6-Mouse IL-5

R^2=99.86% A: 6.459 B: 2.271 C: 7.491 D: 18.010 E: -0.150

Fitting type: 5 Parameter Logistic

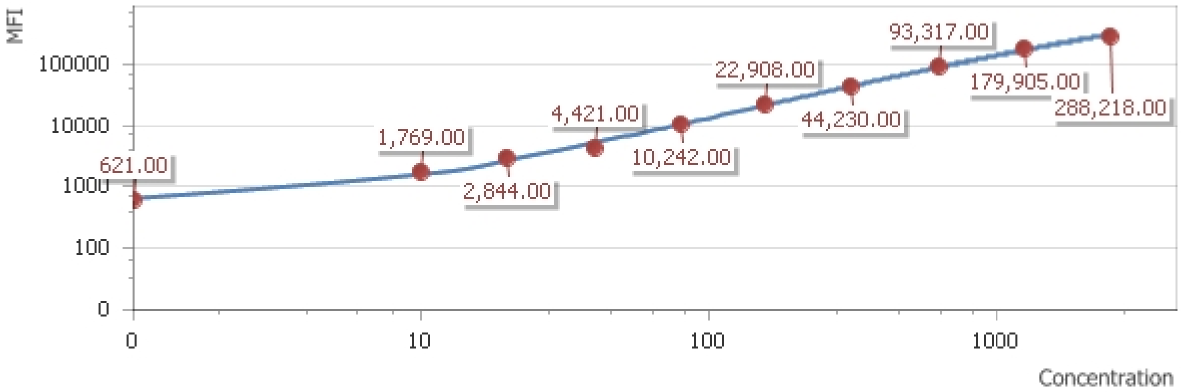

Name	Event #	MFI	SD	CV% (MFI)	Nominal CC pg/mL	Fitted CC pg/mL	Recovery %
Std001	281	621.00	472.58	63.82 %	0.00	0.00	0.00 %
Std002	298	1,769.00	507.42	26.39 %	10.00	11.55	115.48 %
Std003	321	2,844.00	717.21	25.29 %	20.00	20.89	104.44 %
Std005	486	4,421.00	1,018.18	22.19 %	40.00	33.77	84.41 %
Std004	299	10,242.00	2,418.12	22.00 %	80.00	77.49	96.86 %
Std006	280	22,908.00	5,302.33	21.39 %	156.00	165.80	106.28 %
Std007	306	44,230.00	11,191.22	22.59 %	312.50	310.73	99.43 %
Std008	417	93,317.00	23,600.40	23.64 %	625.00	654.69	104.75 %
Std009	300	179,905.00	35,816.84	18.00 %	1,250.00	1,324.66	105.97 %
Std010	293	288,218.00	53,491.10	15.56 %	2,500.00	2,290.38	91.62 %

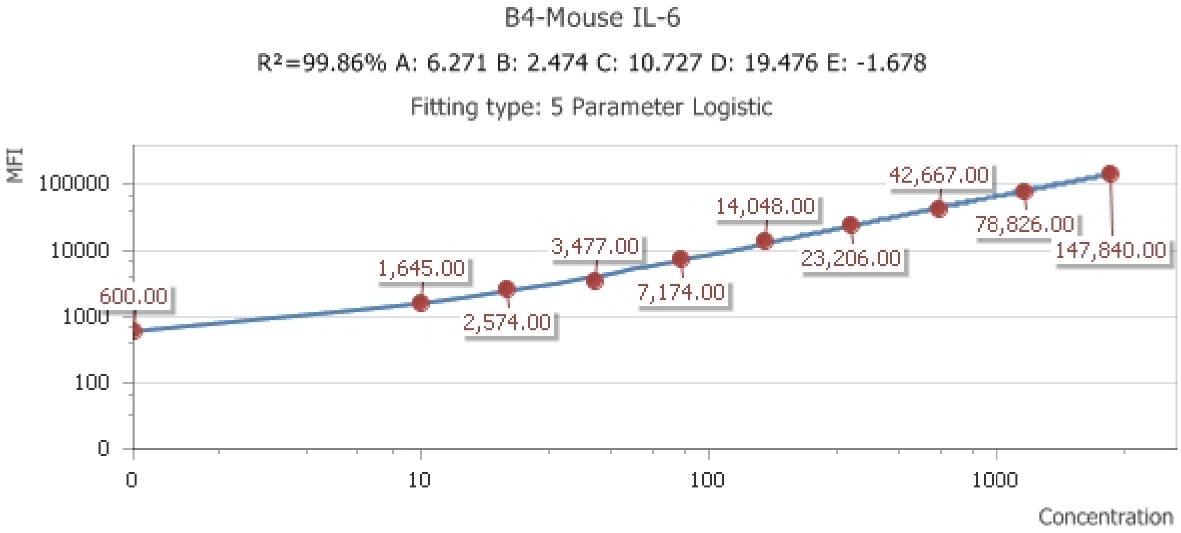

Name	Event #	MFI	SD	CV% (MFI)	Nominal CC pg/mL	Fitted CC pg/mL	Recovery %
Std001	277	600.00	447.00	62.25 %	0.00	0.00	0.00 %
Std002	288	1,645.00	543.93	29.82 %	10.00	10.55	105.46 %
Std003	264	2,574.00	621.21	23.84 %	20.00	21.48	107.39 %
Std005	250	3,477.00	701.83	19.51 %	40.00	32.66	81.66 %
Std004	295	7,174.00	1,404.02	22.95 %	80.00	81.04	101.30 %
Std006	270	14,048.00	2,855.12	18.29 %	156.00	176.23	112.97 %
Std007	259	23,206.00	4,725.79	18.93 %	312.50	309.34	98.99 %
Std008	259	42,667.00	9,503.47	18.94 %	625.00	609.80	97.57 %
Std009	247	78,826.00	16,086.95	19.27 %	1,250.00	1,219.54	97.56 %
Std010	270	147,840.00	28,752.62	17.97 %	2,500.00	2,538.58	101.54 %

A4-Mouse IFN-γ

R^2=99.88% A: 6.273 B: 2.972 C: 10.465 D: 18.253 E: -3.002

Fitting type: 5 Parameter Logistic

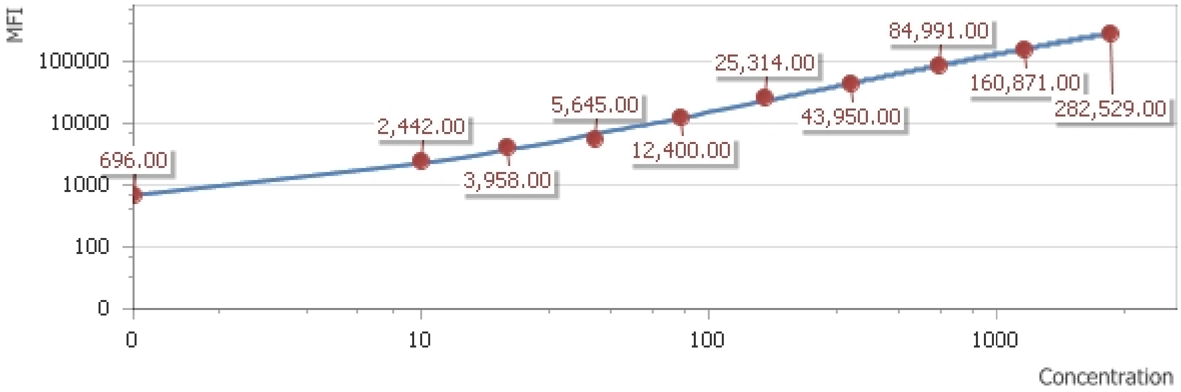

Name	Event #	MFI	SD	CV% (MFI)	Nominal CC pg/mL	Fitted CC pg/mL	Recovery %
Std001	293	696.00	484.44	56.71 %	0.00	0.00	0.00 %
Std002	288	2,442.00	588.78	21.89 %	10.00	10.90	108.97 %
Std003	313	3,958.00	755.01	17.98 %	20.00	21.27	106.36 %
Std005	409	5,645.00	1,209.43	19.40 %	40.00	32.99	82.46 %
Std004	268	12,400.00	2,366.23	17.59 %	80.00	80.28	100.35 %
Std006	290	25,314.00	4,210.21	15.55 %	156.00	171.78	110.12 %
Std007	306	43,950.00	8,085.17	17.15 %	312.50	307.24	98.32 %
Std008	326	84,991.00	17,389.60	17.57 %	625.00	622.20	99.55 %
Std009	295	160,871.00	31,057.50	19.35 %	1,250.00	1,265.72	101.26 %
Std010	300	282,529.00	49,223.43	16.00 %	2,500.00	2,457.87	98.31 %

C8-Mouse TNF

R^2=99.92% A: 6.013 B: 4.702 C: 9.798 D: 13.777 E: -3.217

Fitting type: 5 Parameter Logistic

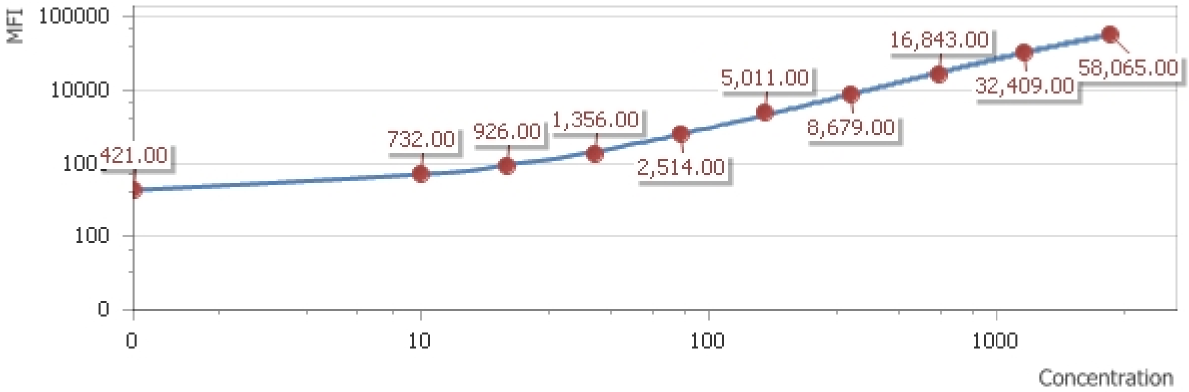

Name	Event #	MFI	SD	CV% (MFI)	Nominal CC pg/mL	Fitted CC pg/mL	Recovery %
Std001	281	421.00	497.41	82.00 %	0.00	0.00	0.00 %
Std002	296	732.00	557.46	61.14 %	10.00	11.52	115.22 %
Std003	328	926.00	557.27	53.56 %	20.00	19.10	95.48 %
Std005	366	1,356.00	292.81	20.09 %	40.00	35.94	89.85 %
Std004	295	2,514.00	550.04	20.26 %	80.00	80.26	100.33 %
Std006	296	5,011.00	888.26	17.23 %	156.00	172.07	110.30 %
Std007	296	8,679.00	1,757.62	19.05 %	312.50	304.29	97.37 %
Std008	276	16,843.00	3,277.66	17.31 %	625.00	605.77	96.92 %
Std009	291	32,409.00	6,722.85	21.14 %	1,250.00	1,243.95	99.52 %
Std010	306	58,065.00	11,822.07	18.46 %	2,500.00	2,541.52	101.66 %

Results Statistics for C4 - Mouse IL-10

Name	Plate Pos.	Clust.	Event #	MFI	SD	CV	Dilution
Std001	1 - A1	Auto	303	510.00	495.93	72.94 %	1.00
Std002	1 - A2	Auto	305	664.00	542.26	62.58 %	1.00
Std003	1 - A3	Auto	323	896.00	570.06	56.32 %	1.00
Std005	1 - A4	Auto	710	1,123.00	255.19	21.43 %	1.00
Std004	1 - A5	Auto	313	1,893.00	432.18	23.57 %	1.00
Std006	1 - A6	Auto	316	3,314.00	686.63	20.41 %	1.00
Std007	1 - A7	Auto	299	5,542.00	863.61	14.36 %	1.00
Std008	1 - A8	Auto	298	10,391.00	1,984.27	17.47 %	1.00
Std009	1 - A9	Auto	281	19,744.00	3,660.17	15.77 %	1.00
Std010	1 - A10	Auto	295	38,379.00	7,883.73	17.81 %	1.00
Test001	1 - B1	Auto	299	1,035.00	988.89	65.23 %	1.00
Test002	1 - B2	Auto	271	687.00	1,329.89	102.06 %	1.00
Test003	1 - B3	Auto	303	899.00	1,321.74	88.80 %	1.00
Test004	1 - B4	Auto	287	723.00	1,339.53	97.59 %	1.00
Test005	1 - B5	Auto	253	516.00	1,120.10	102.56 %	1.00
Test006	1 - B6	Manual	341	929.00	1,183.49	84.57 %	1.00

Quantitative Analyte Results for C4 - Mouse IL-10

Name	Plate Position	Nominal CC	Fitted CC	Final CC	Message
Std001	1 - A1	0.00	0.46	0.46	Fitting: Below standard range
Std002	1 - A2	10.00	9.52	9.52	
Std003	1 - A3	20.00	22.11	22.11	
Std005	1 - A4	40.00	34.98	34.98	
Std004	1 - A5	80.00	80.68	80.68	
Std006	1 - A6	156.00	167.99	167.99	
Std007	1 - A7	312.50	307.30	307.30	
Std008	1 - A8	625.00	615.72	615.72	
Std009	1 - A9	1,250.00	1,230.20	1,230.20	
Std010	1 - A10	2,500.00	2,536.37	2,536.37	
Test001	1 - B1	N/A	29.94	29.94	

Quantitative Analyte Results for C4 - Mouse IL-10

Name	Plate Position	Nominal CC	Fitted CC	Final CC	Message
Test002	1 - B2	N/A	10.74	10.74	
Test003	1 - B3	N/A	22.28	22.28	
Test004	1 - B4	N/A	12.66	12.66	
Test005	1 - B5	N/A	1.19	1.19	
Test006	1 - B6	N/A	23.96	23.96	

Results Statistics for D7 - Mouse IL-12p70

Name	Plate Pos.	Clust.	Event #	MFI	SD	CV	Dilution
Std001	1 - A1	Auto	250	377.00	467.39	80.22 %	1.00
Std002	1 - A2	Auto	270	512.00	577.29	75.20 %	1.00
Std003	1 - A3	Auto	294	751.00	527.62	59.01 %	1.00
Std005	1 - A4	Auto	115	963.00	252.04	24.78 %	1.00
Std004	1 - A5	Auto	273	1,611.00	393.26	21.88 %	1.00
Std006	1 - A6	Auto	264	2,812.00	497.41	17.94 %	1.00
Std007	1 - A7	Auto	267	4,884.00	843.60	13.51 %	1.00
Std008	1 - A8	Auto	288	8,737.00	1,159.39	11.78 %	1.00
Std009	1 - A9	Auto	286	17,389.00	3,275.06	14.15 %	1.00
Std010	1 - A10	Auto	284	31,670.00	5,326.24	13.63 %	1.00
Test001	1 - B1	Auto	325	765.00	871.03	77.20 %	1.00
Test002	1 - B2	Auto	196	0.00	190.14	173.29 %	1.00
Test003	1 - B3	Auto	259	472.00	936.26	102.19 %	1.00
Test004	1 - B4	Auto	243	496.00	994.08	104.85 %	1.00
Test005	1 - B5	Auto	273	440.00	1,034.85	107.37 %	1.00
Test006	1 - B6	Manual	254	549.00	1,023.55	96.11 %	1.00

Quantitative Analyte Results for D7 - Mouse IL-12p70

Name	Plate Position	Nominal CC	Fitted CC	Final CC	Message
Std001	1 - A1	0.00	0.46	0.46	Fitting: Below standard range
Std002	1 - A2	10.00	8.79	8.79	
Std003	1 - A3	20.00	23.04	23.04	
Std005	1 - A4	40.00	36.44	36.44	
Std004	1 - A5	80.00	79.60	79.60	
Std006	1 - A6	156.00	163.36	163.36	
Std007	1 - A7	312.50	312.67	312.67	
Std008	1 - A8	625.00	599.71	599.71	
Std009	1 - A9	1,250.00	1,279.11	1,279.11	
Std010	1 - A10	2,500.00	2,494.00	2,494.00	
Test001	1 - B1	N/A	23.91	23.91	
Test002	1 - B2	N/A	0.00	0.00	Fitting: Below standard range and out of invertable range
Test003	1 - B3	N/A	6.51	6.51	
Test004	1 - B4	N/A	7.88	7.88	

Quantitative Analyte Results for D7 - Mouse IL-12p70

Name	Plate Position	Nominal CC	Fitted CC	Final CC	Message
Test005	1 - B5	N/A	4.69	4.69	
Test006	1 - B6	N/A	10.92	10.92	

Results Statistics for B6 - Mouse IL-21

Name	Plate Pos.	Clust.	Event #	MFI	SD	CV	Dilution
Std001	1 - A1	Auto	257	385.00	433.66	80.82 %	1.00
Std002	1 - A2	Auto	259	1,319.00	565.61	37.15 %	1.00
Std003	1 - A3	Auto	255	2,009.00	624.17	27.46 %	1.00
Std005	1 - A4	Auto	284	2,697.00	514.28	18.82 %	1.00
Std004	1 - A5	Auto	278	5,465.00	1,175.33	21.70 %	1.00
Std006	1 - A6	Auto	279	11,608.00	2,268.38	19.23 %	1.00
Std007	1 - A7	Auto	290	19,916.00	4,451.14	20.01 %	1.00
Std008	1 - A8	Auto	269	33,416.00	6,753.24	19.99 %	1.00
Std009	1 - A9	Auto	310	53,156.00	8,737.89	16.15 %	1.00
Std010	1 - A10	Auto	265	65,089.00	10,310.74	16.28 %	1.00
Test001	1 - B1	Auto	259	1,036.00	951.09	66.91 %	1.00
Test002	1 - B2	Auto	212	235.00	740.37	120.18 %	1.00
Test003	1 - B3	Auto	282	683.00	1,240.75	95.83 %	1.00
Test004	1 - B4	Auto	266	576.00	1,142.16	103.74 %	1.00
Test005	1 - B5	Auto	267	822.00	1,446.28	93.25 %	1.00
Test006	1 - B6	Manual	290	973.00	1,316.36	82.62 %	1.00

Quantitative Analyte Results for B6 - Mouse IL-21

Name	Plate Position	Nominal CC	Fitted CC	Final CC	Message
Std001	1 - A1	0.00	0.00	0.00	Fitting: Below standard range and out of invertable range
Std002	1 - A2	10.00	12.31	12.31	Recovery: Out of range
Std003	1 - A3	20.00	21.35	21.35	
Std005	1 - A4	40.00	30.59	30.59	Recovery: Out of range
Std004	1 - A5	80.00	70.20	70.20	
Std006	1 - A6	156.00	172.27	172.27	
Std007	1 - A7	312.50	341.58	341.58	
Std008	1 - A8	625.00	696.82	696.82	
Std009	1 - A9	1,250.00	1,414.41	1,414.41	
Std010	1 - A10	2,500.00	1,978.12	1,978.12	Recovery: Out of range
Test001	1 - B1	N/A	8.65	8.65	
Test002	1 - B2	N/A	0.00	0.00	Fitting: Below standard range and out of invertable range
Test003	1 - B3	N/A	4.12	4.12	
Test004	1 - B4	N/A	2.73	2.73	
Test005	1 - B5	N/A	5.91	5.91	
Test006	1 - B6	N/A	7.85	7.85	

Results Statistics for A5 - Mouse IL-2

Name	Plate Pos.	Clust.	Event #	MFI	SD	CV	Dilution
Std001	1 - A1	Auto	274	835.00	524.10	47.41 %	1.00
Std002	1 - A2	Auto	237	4,039.00	795.04	16.81 %	1.00
Std003	1 - A3	Auto	242	6,642.00	1,327.67	17.74 %	1.00
Std005	1 - A4	Auto	374	9,937.00	1,805.99	15.94 %	1.00
Std004	1 - A5	Auto	257	20,974.00	4,224.67	18.03 %	1.00
Std006	1 - A6	Auto	251	43,260.00	7,249.91	16.80 %	1.00
Std007	1 - A7	Auto	280	76,339.00	16,047.85	17.93 %	1.00
Std008	1 - A8	Auto	280	142,951.00	27,722.77	17.67 %	1.00
Std009	1 - A9	Auto	271	265,956.00	44,028.03	15.27 %	1.00
Std010	1 - A10	Auto	291	393,580.00	57,123.84	12.43 %	1.00
Test001	1 - B1	Auto	221	2,225.00	969.99	38.24 %	1.00
Test002	1 - B2	Auto	254	1,631.00	1,505.58	69.76 %	1.00
Test003	1 - B3	Auto	221	1,489.00	992.60	59.84 %	1.00
Test004	1 - B4	Auto	260	1,772.00	1,342.68	58.04 %	1.00
Test005	1 - B5	Auto	238	1,595.00	1,422.37	59.53 %	1.00
Test006	1 - B6	Manual	246	1,863.00	1,125.85	48.98 %	1.00

Quantitative Analyte Results for A5 - Mouse IL-2

Name	Plate Position	Nominal CC	Fitted CC	Final CC	Message
Std001	1 - A1	0.00	0.00	0.00	Fitting: Below standard range
Std002	1 - A2	10.00	11.14	11.14	
Std003	1 - A3	20.00	20.79	20.79	
Std005	1 - A4	40.00	33.28	33.28	
Std004	1 - A5	80.00	76.49	76.49	
Std006	1 - A6	156.00	168.84	168.84	
Std007	1 - A7	312.50	316.69	316.69	
Std008	1 - A8	625.00	648.22	648.22	
Std009	1 - A9	1,250.00	1,364.41	1,364.41	
Std010	1 - A10	2,500.00	2,237.01	2,237.01	
Test001	1 - B1	N/A	4.61	4.61	
Test002	1 - B2	N/A	2.56	2.56	
Test003	1 - B3	N/A	2.08	2.08	
Test004	1 - B4	N/A	3.04	3.04	
Test005	1 - B5	N/A	2.44	2.44	
Test006	1 - B6	N/A	3.35	3.35	

Results Statistics for A7 - Mouse IL-4

Name	Plate Pos.	Clust.	Event #	MFI	SD	CV	Dilution
Std001	1 - A1	Auto	238	691.00	505.01	60.65 %	1.00
Std002	1 - A2	Auto	266	1,907.00	550.79	27.81 %	1.00
Std003	1 - A3	Auto	242	2,976.00	880.48	26.49 %	1.00

Results Statistics for A7 - Mouse IL-4

Name	Plate Pos.	Clust.	Event #	MFI	SD	CV	Dilution
Std005	1 - A4	Auto	262	4,410.00	961.65	20.79 %	1.00
Std004	1 - A5	Auto	276	10,805.00	2,680.17	24.43 %	1.00
Std006	1 - A6	Auto	263	24,258.00	5,094.95	20.44 %	1.00
Std007	1 - A7	Auto	284	47,356.00	12,477.38	26.84 %	1.00
Std008	1 - A8	Auto	258	99,188.00	24,156.56	23.96 %	1.00
Std009	1 - A9	Auto	231	181,524.00	40,974.62	20.29 %	1.00
Std010	1 - A10	Auto	283	320,860.00	59,687.99	17.34 %	1.00
Test001	1 - B1	Auto	182	1,369.00	831.55	49.64 %	1.00
Test002	1 - B2	Auto	185	770.00	1,121.22	92.05 %	1.00
Test003	1 - B3	Auto	176	1,062.00	1,232.97	80.11 %	1.00
Test004	1 - B4	Auto	190	1,410.00	1,611.40	78.53 %	1.00
Test005	1 - B5	Auto	194	1,236.00	1,301.35	75.21 %	1.00
Test006	1 - B6	Manual	159	1,520.00	1,280.23	62.31 %	1.00

Quantitative Analyte Results for A7 - Mouse IL-4

Name	Plate Position	Nominal CC	Fitted CC	Final CC	Message
Std001	1 - A1	0.00	0.00	0.00	Fitting: Below standard range and out of invertable range
Std002	1 - A2	10.00	11.89	11.89	
Std003	1 - A3	20.00	20.92	20.92	
Std005	1 - A4	40.00	32.31	32.31	
Std004	1 - A5	80.00	78.64	78.64	
Std006	1 - A6	156.00	168.02	168.02	
Std007	1 - A7	312.50	316.23	316.23	
Std008	1 - A8	625.00	655.85	655.85	
Std009	1 - A9	1,250.00	1,241.86	1,241.86	
Std010	1 - A10	2,500.00	2,386.61	2,386.61	
Test001	1 - B1	N/A	7.07	7.07	
Test002	1 - B2	N/A	1.06	1.06	
Test003	1 - B3	N/A	4.17	4.17	
Test004	1 - B4	N/A	7.45	7.45	
Test005	1 - B5	N/A	5.83	5.83	
Test006	1 - B6	N/A	8.45	8.45	

Results Statistics for A6 - Mouse IL-5

Name	Plate Pos.	Clust.	Event #	MFI	SD	CV	Dilution
Std001	1 - A1	Auto	281	621.00	472.58	63.82 %	1.00
Std002	1 - A2	Auto	298	1,769.00	507.42	26.39 %	1.00
Std003	1 - A3	Auto	321	2,844.00	717.21	25.29 %	1.00
Std005	1 - A4	Auto	486	4,421.00	1,018.18	22.19 %	1.00
Std004	1 - A5	Auto	299	10,242.00	2,418.12	22.00 %	1.00
Std006	1 - A6	Auto	280	22,908.00	5,302.33	21.39 %	1.00

Results Statistics for A6 - Mouse IL-5

Name	Plate Pos.	Clust.	Event #	MFI	SD	CV	Dilution
Std007	1 - A7	Auto	306	44,230.00	11,191.22	22.59 %	1.00
Std008	1 - A8	Auto	417	93,317.00	23,600.40	23.64 %	1.00
Std009	1 - A9	Auto	300	179,905.00	35,816.84	18.00 %	1.00
Std010	1 - A10	Auto	293	288,218.00	53,491.10	15.56 %	1.00
Test001	1 - B1	Auto	357	1,268.00	835.07	55.69 %	1.00
Test002	1 - B2	Auto	284	646.00	1,224.81	97.47 %	1.00
Test003	1 - B3	Auto	301	1,140.00	1,536.71	81.36 %	1.00
Test004	1 - B4	Auto	276	1,163.00	1,746.87	86.86 %	1.00
Test005	1 - B5	Auto	345	1,194.00	1,702.40	83.99 %	1.00
Test006	1 - B6	Manual	265	930.00	1,084.15	80.60 %	1.00

Quantitative Analyte Results for A6 - Mouse IL-5

Name	Plate Position	Nominal CC	Fitted CC	Final CC	Message
Std001	1 - A1	0.00	0.00	0.00	Fitting: Below standard range and out of invertable range
Std002	1 - A2	10.00	11.55	11.55	
Std003	1 - A3	20.00	20.89	20.89	
Std005	1 - A4	40.00	33.77	33.77	
Std004	1 - A5	80.00	77.49	77.49	
Std006	1 - A6	156.00	165.80	165.80	
Std007	1 - A7	312.50	310.73	310.73	
Std008	1 - A8	625.00	654.69	654.69	
Std009	1 - A9	1,250.00	1,324.66	1,324.66	
Std010	1 - A10	2,500.00	2,290.38	2,290.38	
Test001	1 - B1	N/A	6.93	6.93	
Test002	1 - B2	N/A	0.24	0.24	
Test003	1 - B3	N/A	5.71	5.71	
Test004	1 - B4	N/A	5.93	5.93	
Test005	1 - B5	N/A	6.23	6.23	
Test006	1 - B6	N/A	3.63	3.63	

Results Statistics for B4 - Mouse IL-6

Name	Plate Pos.	Clust.	Event #	MFI	SD	CV	Dilution
Std001	1 - A1	Auto	277	600.00	447.00	62.25 %	1.00
Std002	1 - A2	Auto	288	1,645.00	543.93	29.82 %	1.00
Std003	1 - A3	Auto	264	2,574.00	621.21	23.84 %	1.00
Std005	1 - A4	Auto	250	3,477.00	701.83	19.51 %	1.00
Std004	1 - A5	Auto	295	7,174.00	1,404.02	22.95 %	1.00
Std006	1 - A6	Auto	270	14,048.00	2,855.12	18.29 %	1.00
Std007	1 - A7	Auto	259	23,206.00	4,725.79	18.93 %	1.00
Std008	1 - A8	Auto	259	42,667.00	9,503.47	18.94 %	1.00
Std009	1 - A9	Auto	247	78,826.00	16,086.95	19.27 %	1.00

Results Statistics for B4 - Mouse IL-6

Name	Plate Pos.	Clust.	Event #	MFI	SD	CV	Dilution
Std010	1 - A10	Auto	270	147,840.00	28,752.62	17.97 %	1.00
Test001	1 - B1	Auto	418	1,391.00	920.88	52.89 %	1.00
Test002	1 - B2	Auto	336	768.00	1,366.03	96.30 %	1.00
Test003	1 - B3	Auto	384	1,226.00	1,336.75	77.36 %	1.00
Test004	1 - B4	Auto	378	1,250.00	1,457.40	78.65 %	1.00
Test005	1 - B5	Auto	404	1,617.00	1,633.83	75.86 %	1.00
Test006	1 - B6	Manual	392	1,267.00	1,107.87	66.27 %	1.00

Quantitative Analyte Results for B4 - Mouse IL-6

Name	Plate Position	Nominal CC	Fitted CC	Final CC	Message
Std001	1 - A1	0.00	0.00	0.00	Fitting: Below standard range
Std002	1 - A2	10.00	10.55	10.55	
Std003	1 - A3	20.00	21.48	21.48	
Std005	1 - A4	40.00	32.66	32.66	
Std004	1 - A5	80.00	81.04	81.04	
Std006	1 - A6	156.00	176.23	176.23	
Std007	1 - A7	312.50	309.34	309.34	
Std008	1 - A8	625.00	609.80	609.80	
Std009	1 - A9	1,250.00	1,219.54	1,219.54	
Std010	1 - A10	2,500.00	2,538.58	2,538.58	
Test001	1 - B1	N/A	7.73	7.73	
Test002	1 - B2	N/A	1.43	1.43	
Test003	1 - B3	N/A	5.96	5.96	
Test004	1 - B4	N/A	6.21	6.21	
Test005	1 - B5	N/A	10.23	10.23	
Test006	1 - B6	N/A	6.39	6.39	

Results Statistics for A4 - Mouse IFN-γ

Name	Plate Pos.	Clust.	Event #	MFI	SD	CV	Dilution
Std001	1 - A1	Auto	293	696.00	484.44	56.71 %	1.00
Std002	1 - A2	Auto	288	2,442.00	588.78	21.89 %	1.00
Std003	1 - A3	Auto	313	3,958.00	755.01	17.98 %	1.00
Std005	1 - A4	Auto	409	5,645.00	1,209.43	19.40 %	1.00
Std004	1 - A5	Auto	268	12,400.00	2,366.23	17.59 %	1.00
Std006	1 - A6	Auto	290	25,314.00	4,210.21	15.55 %	1.00
Std007	1 - A7	Auto	306	43,950.00	8,085.17	17.15 %	1.00
Std008	1 - A8	Auto	326	84,991.00	17,389.60	17.57 %	1.00
Std009	1 - A9	Auto	295	160,871.00	31,057.50	19.35 %	1.00
Std010	1 - A10	Auto	300	282,529.00	49,223.43	16.00 %	1.00
Test001	1 - B1	Auto	230	1,560.00	844.53	44.58 %	1.00
Test002	1 - B2	Auto	216	974.00	1,346.20	90.49 %	1.00

Results Statistics for A4 - Mouse IFN-γ

Name	Plate Pos.	Clust.	Event #	MFI	SD	CV	Dilution
Test003	1 - B3	Auto	211	1,383.00	1,326.19	70.96 %	1.00
Test004	1 - B4	Auto	241	1,320.00	1,500.76	79.22 %	1.00
Test005	1 - B5	Auto	252	1,319.00	1,478.89	72.83 %	1.00
Test006	1 - B6	Manual	232	1,433.00	1,405.88	67.12 %	1.00

Quantitative Analyte Results for A4 - Mouse IFN-γ

Name	Plate Position	Nominal CC	Fitted CC	Final CC	Message
Std001	1 - A1	0.00	0.00	0.00	Fitting: Below standard range
Std002	1 - A2	10.00	10.90	10.90	
Std003	1 - A3	20.00	21.27	21.27	
Std005	1 - A4	40.00	32.99	32.99	
Std004	1 - A5	80.00	80.28	80.28	
Std006	1 - A6	156.00	171.78	171.78	
Std007	1 - A7	312.50	307.24	307.24	
Std008	1 - A8	625.00	622.20	622.20	
Std009	1 - A9	1,250.00	1,265.72	1,265.72	
Std010	1 - A10	2,500.00	2,457.87	2,457.87	
Test001	1 - B1	N/A	5.07	5.07	
Test002	1 - B2	N/A	1.47	1.47	
Test003	1 - B3	N/A	3.95	3.95	
Test004	1 - B4	N/A	3.55	3.55	
Test005	1 - B5	N/A	3.55	3.55	
Test006	1 - B6	N/A	4.26	4.26	

Results Statistics for C8 - Mouse TNF

Name	Plate Pos.	Clust.	Event #	MFI	SD	CV	Dilution
Std001	1 - A1	Auto	281	421.00	497.41	82.00 %	1.00
Std002	1 - A2	Auto	296	732.00	557.46	61.14 %	1.00
Std003	1 - A3	Auto	328	926.00	557.27	53.56 %	1.00
Std005	1 - A4	Auto	366	1,356.00	292.81	20.09 %	1.00
Std004	1 - A5	Auto	295	2,514.00	550.04	20.26 %	1.00
Std006	1 - A6	Auto	296	5,011.00	888.26	17.23 %	1.00
Std007	1 - A7	Auto	296	8,679.00	1,757.62	19.05 %	1.00
Std008	1 - A8	Auto	276	16,843.00	3,277.66	17.31 %	1.00
Std009	1 - A9	Auto	291	32,409.00	6,722.85	21.14 %	1.00
Std010	1 - A10	Auto	306	58,065.00	11,822.07	18.46 %	1.00
Test001	1 - B1	Auto	218	796.00	917.17	74.93 %	1.00
Test002	1 - B2	Auto	168	0.00	547.82	141.99 %	1.00
Test003	1 - B3	Auto	182	474.00	1,034.67	106.92 %	1.00
Test004	1 - B4	Auto	178	106.00	459.61	127.69 %	1.00
Test005	1 - B5	Auto	148	135.00	719.80	126.72 %	1.00

Results Statistics for C8 - Mouse TNF

Name	Plate Pos.	Clust.	Event #	MFI	SD	CV	Dilution
Test006	1 - B6	Manual	188	336.00	803.75	109.81 %	1.00

Quantitative Analyte Results for C8 - Mouse TNF

Name	Plate Position	Nominal CC	Fitted CC	Final CC	Message
Std001	1 - A1	0.00	0.00	0.00	Fitting: Below standard range
Std002	1 - A2	10.00	11.52	11.52	
Std003	1 - A3	20.00	19.10	19.10	
Std005	1 - A4	40.00	35.94	35.94	
Std004	1 - A5	80.00	80.26	80.26	
Std006	1 - A6	156.00	172.07	172.07	
Std007	1 - A7	312.50	304.29	304.29	
Std008	1 - A8	625.00	605.77	605.77	
Std009	1 - A9	1,250.00	1,243.95	1,243.95	
Std010	1 - A10	2,500.00	2,541.52	2,541.52	
Test001	1 - B1	N/A	14.01	14.01	
Test002	1 - B2	N/A	0.00	0.00	Fitting: Below standard range and out of invertable range
Test003	1 - B3	N/A	1.78	1.78	
Test004	1 - B4	N/A	0.00	0.00	Fitting: Below standard range and out of invertable range
Test005	1 - B5	N/A	0.00	0.00	Fitting: Below standard range and out of invertable range
Test006	1 - B6	N/A	0.00	0.00	Fitting: Below standard range and out of invertable range

Sample File Assignment

Sample Name	File Name
Std001	B01 0 pg_mL.fcs
Std002	B02 1_256.fcs
Std003	B03 1_128.fcs
Std005	B04 1_64.fcs
Std004	B05 1_32.fcs
Std006	B06 1_16.fcs
Std007	B07 1_8.fcs
Std008	B08 1_4.fcs
Std009	B09 1_2.fcs
Std010	B10 Top Standard.fcs
Test001	A01 Untreated_24H.fcs
Test002	A02 Untreated_48H.fcs
Test003	A03 139A_24H_2.fcs
Test004	A04 139A_48H_2.fcs
Test005	A05 224D_24H_2.fcs
Test006	A06 224D_48H_2.fcs
